# Avasopasem manganese treatment for severe oral mucositis from chemoradiotherapy for locally advanced head and neck cancer: phase 3 randomized controlled trial (ROMAN)

**DOI:** 10.1016/j.eclinm.2025.103539

**Published:** 2025-10-08

**Authors:** Carryn Anderson, Christopher M. Lee, Joseph Randall Kelley, Gary V. Walker, Neal E. Dunlap, Voichita C. Bar-Ad, Douglas A. Miller, Vernon J. King, Abhinand V. Peddada, Douglas F. Ciuba, Francois Vincent, Brian C. Muzyka, Amanda Lynn Gillespie-Twardy, Stephen T. Sonis, Jon Holmlund, Robert A. Beardsley, Eugene P. Kennedy, Deborah Saunders

**Affiliations:** a University of Iowa Health Care, Iowa City, IA, USA; b Cancer Care Northwest, Spokane, WA, USA; c University of Tennessee Medical Center, Memphis, TN, USA; Currently at GenesisCare-Asheville, NC, USA; d Banner MD Anderson Cancer Center, Gilbert, AZ, USA; e University of Louisville/James Graham Brown Cancer Center, Louisville, KY, USA; f Thomas Jefferson University Hospital, Bodine Center for Cancer Treatment, Philadelphia, PA, USA; g Hackensack Meridian Health, Jersey Shore University Medical Center Hope Tower, Neptune, NJ, USA; h St. Mary's Hospital Regional Medical Center, Grand Junction, CO, USA; i William N. Pennington Cancer Institute, Renown Regional Medical Center, Reno, NV, USA; j John B. Amos Cancer Center, IACT Health, Columbus, GA, USA; k Centre Intégré Universitaire de Sante et Services Sociaux, Mauricie-Centre-du Quebec Site CHAUR, Trois-Rivières, Canada; l School of Dental Medicine at East Carolina University, Greenville, NC, USA; m Oncology and Hematology Associates of Southwest Virginia, Inc., DBA Blue Ridge Cancer Care, Roanoke, VA, USA; n Primary Endpoint Solutions, Boston, MA, USA; o Dana-Farber/Brigham and Women's Cancer Center, Boston, MA, USA; p Harvard School of Dental Medicine, Boston, MA, USA; q Galera Therapeutics, Inc, Malvern, PA, USA; r Northeast Cancer Centre of Health Sciences North, Health Sciences North, Northern Ontario School of Medicine, Sudbury, Canada

**Keywords:** Avasopasem, Oral mucositis, Head and neck cancer, Radiation therapy, Cisplatin

## Abstract

**Background:**

Of patients who receive standard concomitant chemoradiation (CRT; intensity-modulated radiation therapy [IMRT] plus cisplatin) for locally advanced head and neck cancer (HNC), approximately two-thirds will develop severe oral mucositis (SOM), limiting their ability to eat solids (WHO grade 3) or drink liquids (WHO grade 4). In a randomized, double-blind phase 2b trial, avasopasem manganese substantially reduced duration and incidence of SOM versus placebo. This phase 3 trial further assessed avasopasem's reduction of SOM due to CRT.

**Methods:**

In this double-blind, placebo-controlled trial (Clinicaltrials.gov: NCT03689712), patients receiving 60–72 Gy IMRT (>50 Gy to ≥2 oral mucosal sites) plus cisplatin (Q3W or QW) were randomized 3:2 to avasopasem 90 mg or placebo before each RT fraction. SOM was assessed twice weekly during IMRT, then weekly for 2 weeks. First subject was enrolled 03 October 2018 and last subject completed OM follow-up 13 September 2021. Last subject completed long-term follow-up 30 August 2021. Primary endpoint was SOM incidence through end of IMRT. Secondary endpoints included SOM duration, time to onset, grade 4 incidence and duration, tumor outcomes, and renal function.

**Findings:**

455 patients were randomized; 407 (241 avasopasem/166 placebo) were included in the primary analysis population. Statistically significant reductions in SOM incidence (54% vs 64%; relative risk = 0·84, *p* = 0·045, 95% CI 0·71, 1·00) and SOM duration (*p* = 0·002; median, 8 vs 18 days) were observed. SOM onset was nominally delayed (*p* = 0·002; median, 49 vs 38 days). Grade 4 OM incidence and days were not significantly reduced by avasopasem, 27% (*p* = 0·052) and 24% (*p* = 0·143), respectively. Adverse event frequencies were comparable between treatments. After 1 year, tumor outcomes were maintained and two-year overall survival showed: avasopasem 89% (95% CI: 84–93) versus placebo 93% (95% CI: 88–96).

**Interpretation:**

The primary endpoint of incidence was not as improved as predicted by the Phase 2b trial and avasopasem's contribution to adverse events could not be excluded. For these reasons and others discussed, ROMAN did not provide a sufficiently favorable benefit-risk determination for FDA approval. A confirmatory phase 3 trial was requested.

**Funding:**

Provided by Galera Therapeutics, Inc.

**Research in context**

**Evidence before this study**

There are no United States Food and Drug Administration (FDA)-approved drugs to mitigate chemoradiation-induced severe oral mucositis (SOM) in solid tumors and other recent phase 2b/3 trial drugs failed to meet their primary endpoints. Avasopasem manganese (AVA) decreased duration, incidence, severity and delayed time to onset of SOM in a randomized phase 2b trial of 30 mg and 90 mg versus placebo. Retrospective analysis of kidney function revealed AVA may mitigate cisplatin-induced nephrotoxicity.

**Added value of this study**

The ROMAN phase 3 trial showed efficacy of AVA 90 mg in reducing incidence, duration, and severity as well as delaying time to onset of SOM, and 1-year chronic kidney disease rates were cut in half with AVA. However, the primary endpoint of incidence of SOM was not as improved as predicted by the Phase 2b trial and AVA's contribution to adverse events could not be excluded. Lessons learned from ROMAN may guide future trial design.

**Implications of all the available evidence**

Treatment of chemoradiation-induced SOM remains an unmet medical need. Future trial design may benefit from inclusion of a multiparametric endpoint, patient-reported outcomes, and resource management analysis. This novel class of drugs has the potential to mitigate radiation and cisplatin injury and warrants further study.

## Introduction

Intensity-modulated radiation therapy (IMRT) plus cisplatin is standard for locoregionally advanced head and neck cancer (HNC).^1^, ^2^, ^3^ Of patients receiving chemoradiotherapy (CRT), 65%–70% will suffer severe oral mucositis (SOM),^4^^,^^5^ defined as World Health Organization (WHO) grade 3 or 4, limiting their ability to eat solids (grade 3) or drink liquids (grade 4, often necessitating feeding tubes). SOM is associated with considerable pain, quality of life impairment, infection potential, hospitalization, and treatment breaks that can decrease oncologic efficacy.^6^ Current management focuses on symptoms after injury.^7^ There are no United States Food and Drug Administration (FDA)-approved drugs to reduce SOM in HNC.

Radiation therapy (RT)-induced bursts of superoxide are critical in initiation of OM.^8^ Avasopasem manganese (avasopasem), an investigational selective dismutase mimetic, rapidly converts superoxide to hydrogen peroxide, protecting normal cells from RT damage.^9^^,^^10^ In a randomized, double-blind phase 2b trial, avasopasem 90 mg substantially reduced duration and incidence of SOM due to CRT in patients with HNC versus placebo, with an acceptable safety profile.^11^ Two-year tumor outcomes and overall survival were maintained.^12^ These data secured avasopasem Breakthrough Therapy Designation from the FDA. Post hoc analysis of renal function in a subset of patients also suggested avasopasem preserved renal function. Reduction in Oral Mucositis with Avasopasem Manganese (ROMAN) trial further assessed safety and efficacy of avasopasem to reduce SOM and preserve renal function in this same population.

## Methods

### Study design

ROMAN was a randomized, double-blind, placebo-controlled phase 3 trial (Clinicaltrials.gov: NCT03689712), sponsored and financially supported by Galera Therapeutics.

### Participants

Patients had locally advanced, nonmetastatic oral cavity (OC) or oropharynx (OP) squamous carcinoma and planned treatment with standard fractionation IMRT (2–2·2 Gy/fraction, total dose 60–72 Gy) and concurrent cisplatin (100 mg/m^2^ Q3W x 3 or 40 mg/m^2^ QW x 6–7) administered definitively or after surgical resection. Radiation plans included ≥2 oral mucosal sites within the cumulative 50-Gy isodose line and were centrally reviewed by an independent radiation oncologist blinded to treatment assignment. ECOG performance status ≤2, and adequate marrow, renal, and hepatic functions were required. Prophylactic percutaneous endoscopic gastrostomy tube placement was allowed, but patients were required to be able to eat soft solids at enrollment.

### Ethics

ROMAN was approved by each institution's ethics committee, with the lead center's approval number being 20181417 from Western Institutional Review Board (see Supplementary for additional approval references) and was conducted according to Good Clinical Practice and the Declaration of Helsinki. The trial was activated at 97 centers in the United States and Canada. All patients provided written informed consent. Data were anonymized to protect patients' identities.

### Randomization and masking

Patients were randomized 3:2 to avasopasem 90 mg or placebo using interactive response technology (ALMAC, Craigavon, United Kingdom). Enrollment was stratified by cisplatin schedule (QW vs Q3W) and treatment setting (postoperative vs definitive treatment). Trial was double-blinded. Personnel who enrolled and those who assigned participants to interventions did not have access to the random allocation sequence.

### Procedures

Study infusion was administered intravenously in 250 mL normal saline over 60 min, ending within 60 min before each IMRT session. Oral rinses, limited to saline, sodium bicarbonate, sodium bicarbonate/saline, lidocaine, and antifungal agents, were permitted. Other concurrent pharmaceuticals, devices, and low-level laser therapy to reduce OM were excluded. Supportive care per ASCO and MASCC guidelines, including for nausea and vomiting, was employed.

Oral mucositis was assessed by trained evaluators (physicians, dentists, registered nurses, physician assistants and dental hygienists).^13^ Assessment training and central review of WHO assessments were performed by Primary Endpoint Solutions (Waltham, MA) to ensure (1) assessments were performed consistently using standardized questions, oral cavity examination technique and order, and data collection; and (2) WHO grade scoring was correctly assigned per findings. Evaluators completed a 7-module online training program (including competency testing for each module). Refresher training was mandated if >4 weeks elapsed between training completion and first patient evaluation.

Evaluators assessed OM twice per week during IMRT and weekly for 2 weeks thereafter using WHO criteria: grade 0, no mucositis; grade 1, pain and erythema; grade 2, ulceration, able to eat solid food; grade 3, ulceration, able to only drink liquids; and grade 4, ulceration, inability to eat or drink, requiring tube or parenteral feeding. A modified WHO scoring system used in published phase 1b/2a and 2b studies of avasopasem was utilized.^11^^,^^14^ In this modification, evaluators elucidated whether oral pain caused dietary compromise. If instead the cause was a confounding factor (e.g., dysgeusia, edentulism, nausea, thick mucus, throat pain, functional dysphagia), the score was based on what the patient said they could eat absent these factors. Accuracy and consistency of WHO scores were monitored in a blinded fashion, and inconsistencies were queried before final WHO scores were determined. Additional assessments included opioid use and reasons for use, whether and when gastrostomy tubes were inserted and used, and renal function.

Adverse events (AEs) were assessed using National Cancer Institute Common Terminology Criteria for Adverse Events, version 5·0. Dose reductions of 25% from initial 90-mg dose were required for grade 2 or greater hypotension occurring within 2 h after infusion start or for grade 3 to 4 AEs judged likely attributable to study infusion. Two dose reductions (50% cumulative) were permitted, with discontinuation of future infusions if still unable to tolerate treatment.

### Outcomes

The primary efficacy endpoint was SOM incidence from IMRT start through end of IMRT. Secondary efficacy endpoints included SOM duration (days) and grade 4 duration from IMRT start through 2 weeks post-IMRT, and grade 4 OM incidence thru end of IMRT. SOM duration was defined as number of days from first SOM occurrence until first grade 0–2 OM score with no subsequent SOM. If SOM never developed in a patient, a duration of zero was assigned. Grade 4 OM duration was defined similarly: days from first grade 4 occurrence until first grade 0–3 OM score with no subsequent grade 4 OM. If grade 4 OM never developed, a duration of zero was assigned. Safety and tumor outcomes were also collected as secondary outcomes.

Exploratory efficacy endpoints included days to SOM onset, SOM incidence at progressive RT/time landmarks, and SOM duration in the subset of patients that developed SOM. The effect of treatment assignment on kidney function throughout the first year post- IMRT was collected as secondary outcome. Narcotics use and gastrostomy tube placement and use were also collected as secondary outcomes.

Following treatment, patients entered follow-up in which tumor progression, survival, and renal function were assessed every 3 months during the first year, and survival was assessed every 4 months during the second year.

### Statistics

The planned primary analysis population size was 400 patients, with 95% power to detect a 34% relative reduction of SOM incidence through end of IMRT. After consultation with the FDA, the intent-to-treat (ITT) primary analysis population was defined as all randomized patients receiving at least one dose of avasopasem or placebo, but excluding those whose dosing was prematurely terminated for administrative reasons while investigating a drug product issue involving appearance of fine visible particulate. Efficacy and safety were assessed in the ITT population, stratified per above.

Patients prematurely lost to OM assessment had values for primary and secondary endpoints imputed using a multiple imputation process designed in discussion with the FDA. For each such patient, other patients were randomly selected from an “imputation set” of patients in the same treatment arm who had completed OM assessment and at least as much IMRT as the patient lost to follow-up. This process was repeated 50 times, generating 50 sets of observed and imputed values, from which a final result was generated for each patient lost.

Severe oral mucositis and grade 4 OM incidences were analyzed by Cochran-Mantel-Haenszel (CMH) testing stratified by the two factors used in randomization (e.g., cisplatin schedule, treatment setting). Inferential statistics for SOM and grade 4 OM durations were analyzed by ANOVA using least squares mean (LSM) values; median values were also reported.

The primary endpoint was tested at a two-sided type 1 error rate of 0·05. To control overall type 1 error at 0·05, the primary endpoint was first tested at a two-sided 0·05 level, then Holm-Bonferroni multiplicity testing was conducted on the three secondary endpoints applying familywise two-sided alpha of 0·05. For Table 1 and Appendix Table A1, Chi-squared tests or Fisher's exact tests, where appropriate, were used to compare categorical and two-sample t-tests were used to compare numeric characteristics between avasopasem and placebo groups. All statistical testing was two-sided and assessed for significance at the 0·05 level. Statistical analyses were conducted with SAS® (version 9·2 or higher; SAS Institute, Cary, NC).

**Table 1:** Baseline patient and disease characteristics.

Characteristic	AVA 90 mg (n = 241)	PBO (n = 166)	All patients (N = 407)
Sex, n (%)			
Male	202 (84)	149 (90)	351 (86)
Female	39 (16)	17 (10)	56 (14)
Age, years			
Mean (SD)	60·4 (9·37)	61·2 (8·64)	60·8 (9·08)
Median (range)	61 (32–81)	61 (34–84)	61 (32–84)
ECOG PS, n (%)^a^^,^^b^			
0	186 (77)	115 (69)	301 (74)
1	54 (22)	50 (30)	104 (26)
2	1 (<1)	1 (1)	2 (<1)
Tumor site, n (%)			
Oropharyngeal	194 (80)	141 (85)	335 (82)
Oral cavity	38 (16)	21 (13)	59 (14)
Unknown	9 (4)	4 (2)	13 (3)
Treatment type, n (%)			
Definitive	195 (81)	134 (81)	329 (81)
Postoperative treatment	46 (19)	32 (19)	78 (19)
Overall stage, n (%)^b^^,^^c^			
I	79 (33)	42 (25)	121 (30)
II	72 (30)	67 (40)	139 (34)
III	53 (22)	34 (20)	87 (21)
IVa	28 (12)	18 (11)	46 (11)
IVb	8 (3)	5 (3)	13 (3)
Unknown/not reported	1	0	1
Tumor HPV status, n (%)^b^			
Positive	193 (81)	134 (83)	327 (82)
Negative	44 (19)	27 (17)	71 (18)
Unknown	4	5	9
Cisplatin schedule, n (%)			
Every 3 weeks	102 (42)	74 (45)	176 (43)
Weekly	139 (58)	92 (55)	231 (57)
Planned sites to receive ≥50 Gy, n (%)^b^			
1	0 (0)	0 (0)	0 (0)
2	11 (5)	5 (3)	16 (4)
3	40 (17)	27 (16)	67 (16)
4	76 (32)	48 (29)	124 (30)
5	47 (20)	45 (27)	92 (23)
>5	67 (28)	41 (25)	108 (27)
Tobacco use, n (%)^b^			
Never used	94 (39)	50 (30)	144 (35)
Past	105 (44)	85 (51)	190 (47)
Current	42 (17)	31 (19)	73 (18)
Current alcohol use, n (%)			
Yes	119 (49)	97 (59)	216 (53)
No	122 (51)	68 (41)	190 (47)
Not reported	0	1	1

AVA, avasopasem Mn; ECOG PS, Eastern Cooperative Oncology Group performance status; Gy, gray; HPV, human papillomavirus; PBO, placebo; SD, standard deviation; TNM, tumor, node, metastasis.

a ECOG: 0 = Fully active; 1 = Restricted in activity; 2 = Ambulatory 50% of waking hours.

b Because of rounding, percentages may not total 100.

c According to American Joint Committee on Cancer, 8th Edition.

### Role of the funding source

Galera Therapeutics was responsible for study design, though Dr. Anderson and Dr. Saunders provided feedback (uncompensated) based on their experience with the phase 2b trial. Galera paid an independent Clinical Research Organization and Primary Endpoint Solutions for data collection and to query data for clarification. PIs had final control over submitted data and attributions for adverse events. Galera paid for independent statistical analysis of the data. Galera co-authors participated in interpretation of the data but all co-authors independently reviewed the data analysis and provided the consensus interpretation here-in. Co-authors employed by Galera Therapeutics provided data and edited the manuscript for clarity but not content.

## Results

**Fig. 1:**
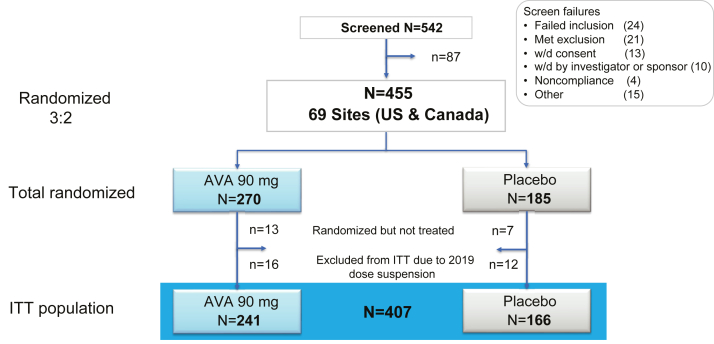
**Consort diagram**. AVA, avasopasem Mn; ITT, intent to treat; w/d, withdrawn.

Four hundred fifty-five patients were randomized between October 3, 2018 and August 13, 2021 at 69 of 97 activated US and Canadian sites. Twenty patients were randomized but never received avasopasem or placebo, and 28 were excluded per discussion with FDA because of administrative suspension of dosing, leaving a primary ITT safety/efficacy population of 407 (241 avasopasem/166 placebo, Fig. 1). Baseline patient characteristics were balanced between arms, with slight differences in uncontrolled characteristics (ex. TNM stage) not expected to impact SOM results (Table 1, Appendix Table A1). Importantly, number of oral cavity sites receiving 50+ Gy between arms was substantially balanced. Radiation plan adherence to protocol was confirmed in all cases. Treatment delivery is summarized in Table 2. Thirteen avasopasem patients and one placebo patient withdrew consent before receiving at least 60 Gy of standard IMRT on protocol, impacting reported radiation therapy and cisplatin delivery in the avasopasem arm. This does not mean that patients stopped cisplatin/IMRT prematurely, we just did not have consent to collect their treatment delivery data after consent was withdrawn.

**Table 2:** Treatment delivery.

Variable	AVA 90 mg (N = 241)	PBO (N = 166)
Total IMRT dose, Gy		
Mean (SD)	64·4 (14·18)	68·2 (5·29)
Median (range)	70 (8–70)	70 (13–71)
RT treatment breaks ≥5 consecutive fractions, n (%)	19 (8)	16 (10)
Cisplatin Median RDI, %		
Q3week	90	88
Weekly	99	97
Planned AVA/PBO doses received		
Mean, % (SD)	87·0 (26·02)	96·1 (9·90)
Median (range)	100 (2·9, 103·3)	100 (18·8, 110·0)
25% dose reductions, (n%)		
One	23 (10)	9 (5)
Two	14 (6)	2 (1)

AVA, avasopasem Mn; Gy, gray; IMRT, intensity-modulated radiation therapy; PBO, placebo; RT, radiation therapy; RDI, relative dose intensity; SD, standard deviation.

The initially assigned WHO score was consistent with source data in 6820 (94·9%) of 7185 OM assessments; inconsistencies were queried and resolved to ensure 100% scoring accuracy.

### SOM efficacy

**Fig. 2:**
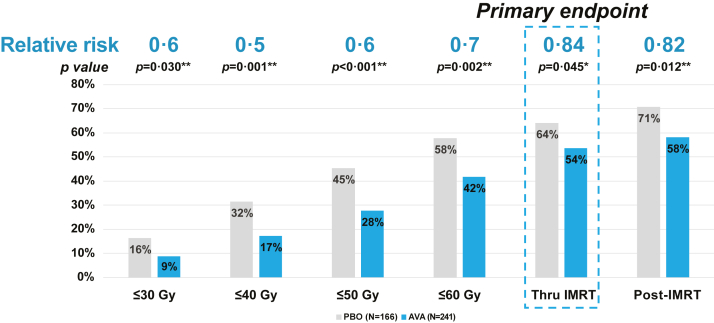
**SOM incidence (ITT Population)**. AVA, avasopasem Mn; Gy, gray; IMRT, intensity-modulated radiation therapy; ITT, intent to treat; PBO, placebo; SAP, statistical analysis plan; SOM, severe oral mucositis. ∗Statistically significant. ∗∗Exploratory endpoints, not formally tested as part of SAP.

Severe oral mucositis incidence through IMRT was significantly reduced by avasopasem compared to placebo 54% vs 64% [AVA, 54%, n = 130/241, (95% CI 47·1, 60·6); placebo, 64%, n = 106/166, (95% CI 56·7, 71·4); relative risk = 0·84, *p* = 0·045, 95% CI 0·71, 1·00; Table 3]. SOM incidence through IMRT in patients who did not require imputation for missing data (211/241 avasopasem, 161/166 placebo) was similar: 56% versus 65%. Incidences at prespecified RT landmarks showed significant benefit to avasopasem throughout IMRT, including 2 weeks post-IMRT (58% versus 71% [AVA, 58%, n = 140/241, (95% CI 51·8, 70·7); placebo, 71%, n = 117/166, (95% CI 63·7, 77·7); relative risk = 0·82, *p* = 0·012, 95% CI 0·71, 0·96], with late onset (occurring ∼ Day 43 during IMRT) SOM in the AVA arm narrowing the difference at the end of IMRT (Fig. 2, Appendix Figure A1).

**Table 3:** Efficacy results.

	AVA	PBO	AVA vs PBO	
	(N = 241)	(N = 166)	Relative Δ	*p* value
**Primary endpoint**^c^				
SOM incidence through IMRT (95% CI)	(N = 130) 54% (47·1, 60·6)	(N = 106) 64% (56·7, 71·4)	(0·84) 16% (0·707, 0·996)	0·045^a^
**Secondary and exploratory endpoints**^c^				
SOM days^d^ through f/u				
Median (range)	8 (0, 54)	18 (0, 67)		
LS Mean (SE)	13·7 (1·29)	18·7 (1·42)	56%	0·002^a^
Grade 4 OM incidence through IMRT (95% CI)	(N = 58) 24% (18·2, 29·5)	(N = 55) 33% (25·5, 40·0)	27%	0·052
Grade 4 OM days^d^ through f/u				
Median (range)	0 (0, 51)	0 (0, 61)		
LS Mean (SE)	5·5 (0·91)	7·2 (1·01)	24%	0·143
SOM onset, median days (95% CI)	49 (43, 54)	38 (35, 44)	29%	0·002^b^
SOM days^d^ through f/u in subset that experienced SOM				
Median (range)	22·5 (1, 54)	29 (3, 67)		
LS mean (SE)	23·3 (1·41)	26·0 (1·45)		
SOM incidence through 30 Gy (95% CI)	(N = 22) 9% (5·4, 12·7)	(N = 27) 16% (10·7, 22·0)	40%	0·030^b^
SOM incidence through 40 Gy (95% CI)	(N = 41) 17% (12·3, 22·1)	(N = 53) 32% (24·4, 38·6)	50%	0·001^b^
SOM incidence through 50 Gy (95% CI)	(N = 67) 28% (22·0, 33·6)	(N = 75) 45% (37·8, 53·0)	40%	<0·001^b^
SOM incidence through 60 Gy (95% CI)	(N = 101) 42% (35·2, 48·1)	(N = 96) 58% (50·3, 65·5)	30%	0·002^b^
SOM incidence through f/u (95% CI)	(N = 140) 58% (51·8, 64·9)	(N = 118) 71% (63·7, 77·7)	18%	0·012^b^

AVA, avasopasem Mn; CI, confidence interval; f/u, follow-up; Gy, gray; IMRT, intensity-modulated radiation therapy; LS, least square; OM, oral mucositis; PBO, placebo; SE, standard error; SOM, severe OM.

a Statistically significant.

b Exploratory endpoints, not formally tested as part of SAP.

c Endpoints estimated using stratification factors at randomization—planned cisplatin schedule (weekly vs every 3 weeks) and treatment setting (postoperative vs definitive).

d Days (duration) of SOM was calculated using the full ITT population. Patients who did not develop SOM (or grade 4 SOM) were assigned a value of 0 days.

There was significant reduction in SOM duration (56% reduction in median, AVA 8 vs placebo 18 days, median difference = 10 days; n = 241 and n = 166; 27% in LSM, 13·7 (95% CI 11·1, 16·2) vs 18·7 days (95% CI 16·0, 21·5), LSM difference = 5·1 days, *p* = 0·002, 95% CI 1·8, 8·4*)*. Grade 4 OM incidence (24% vs 33%, *p* = 0·052), and duration (LSM 5·5 vs 7·2 days, *p* = 0·143) were not significantly reduced with avasopasem.

Nominal improvement was observed with avasopasem on multiple exploratory SOM endpoints (Table 3): delaying time to SOM onset [AVA median 49 (95% CI 43·0, 54·0) vs placebo 38 days (95% CI 35·0, 44·0); n = 241 and n = 166, *p* = 0·002; Appendix Figure A1]; and reducing SOM duration in only those patients who experienced SOM (median 22·5 vs 29 days, n = 130 and n = 106) rather than the entire ITT population. SOM incidence and duration by cisplatin schedule and treatment setting, and primary tumor type subpopulations suggest similar benefit, except that postoperative patients may not have benefited as much as definitive patients for SOM Incidence (post hoc analysis, *p* = 0·04, Appendix Figure A2).

### Safety

**Fig. 3:**
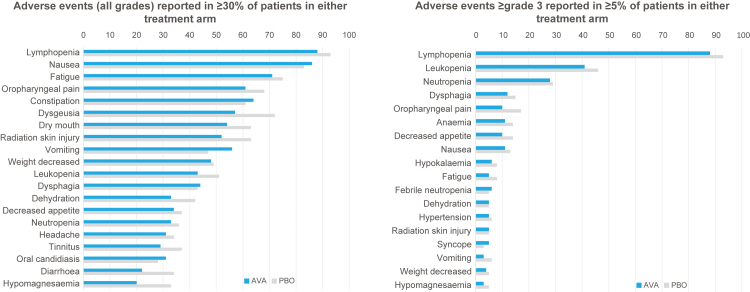
**Most frequent adverse events**. AVA, avasopasem Mn; ITT, intent to treat; PBO, placebo.

All-grade, grade 3+, and serious AEs occurred with similar or lower frequencies for avasopasem versus placebo (Fig. 3; Appendix Tables A2–A4) except as noted below. Observed AEs appeared primarily attributable to the underlying IMRT/cisplatin regimen and known comorbidities in the HNC population.

Avasopasem transiently potentiates nitric oxide (NO), and this can manifest as vasodilatory events such as hypotension or dizziness (orthostatic symptoms). However, such effects appeared limited (Appendix Tables A2–A4). Incidences of all-grade hypotension (23% vs 19%, avasopasem vs placebo) and dizziness (28% vs 23%) were increased, but grade 3+ hypotension (3% vs 4%) was similar and grade 3+ dizziness occurred rarely. End-of-infusion decreases in systolic blood pressure greater than 20 mm Hg vs pre-infusion were more common with avasopasem (22% vs 9% day 1; 15% vs 5% day 22), but generally not associated with concurrent AEs. All-grade nausea and vomiting were slightly more common, but grade 3+ nausea and vomiting were less frequent, with avasopasem. Grade 3+ syncope (5% vs 3%) and pulmonary embolism (3% vs 1%) were slightly increased with avasopasem.

Decreased incidences of several AEs attributable to RT were noted, with radiation skin injury (52% vs 63%), dry mouth (54% vs 63%), and dysgeusia (57% vs 72%) less frequent for avasopasem. Certain cisplatin-associated AEs also appeared decreased. Specifically, acute kidney injury (2% vs 8%; grade 3 + 1% vs 4%), hypomagnesemia (20% vs 33%; grade 3 + 3·3% vs 5·4%), and increased creatinine (16% vs 21%; grade 3 + 1·2% vs 2·4%) were less frequent for avasopasem, as was tinnitus (29% vs 37%).

### Additional exploratory endpoints

Overall, 34% of patients had a gastrostomy tube placed prior to IMRT start. After IMRT start, gastrostomy tube placement was less common with avasopasem (21% vs 26%), as was usage overall (48% vs 61%) and usage starting after IMRT day 1 (44% vs 55%). Fewer avasopasem patients used narcotics at any time (81% vs 86%), or after IMRT start (48% vs 55%); OM (35% vs 40%) or other mouth/throat pain (38% vs 42%) were stated reasons for narcotic use less frequently for avasopasem than placebo.

At one-year, renal function appeared better preserved in avasopasem patients and chronic kidney disease (CKD) (eGFR<60 mL/min/1·73 m^2^) was reduced (14% vs 28%; nominal *p* = 0·009).

One-year tumor outcomes were comparable for avasopasem and placebo, with confidence intervals overlapping for all outcomes. Overall survival at one year was 95% (95% CI: 91–97) in the avasopasem arm vs 97% (95% CI: 93–99) in the placebo arm. Progression-free survival (PFS), locoregional control (LRC), and distant disease control (DDC) were 88% (95% CI: 85–93) for avasopasem vs 93% (95% CI: 89–97) for placebo, 94% (95% CI: 91–98) vs 98% (95% CI: 95–100), and 96% (95% CI: 92–98) vs 97% (95% CI: 93–99), respectively. Follow-up for overall survival through 2 years showed statistically similar outcomes between arms 89% (95% CI: 84–93) vs 93% (95% CI: 88–96). There were 9 excess deaths in the avasopasem arm: 5 deaths were clustered at one enrolment site. Four deaths occurred during chemoRT on the avasopasem arm (3 received weekly 40 mg/m^2^ and 1 received 100 mg/m2; RT dose at death: 48–60 Gy). All 4 of these deaths were considered unrelated to study drug by local Principal Investigators: 2 cardiac arrest, 1 pulmonary embolism and 1 undetermined natural causes. Four deaths on the avasopasem arm occurred 18–21 months post randomization (1 secondary cancer, 1 progression and 2 unknown). Unplanned secondary subset analysis of 2-year overall survival with avasopasem vs placebo in patients with HPV + OP, AJCC v7 Stage III/IVa/IVb, OC location, HPV- OP and treatment with definitive chemoRT showed statistically similar outcomes (overlapping 95% CI).

## Discussion

Severe oral mucositis is a common debilitating side effect of RT for HNC patients, and its mitigation is a substantial unmet medical need. With no FDA-approved drug to limit SOM development, MASCC/ISOO Clinical Practice guidelines currently focus on symptom management strategies.^7^ Unlike palliation, avasopasem acts to block a critical underlying mechanism driving SOM. Unlike low-light laser therapy, AVA's systemic delivery impacts mucositis development beyond where instruments can reach and extends to other organs, like the kidneys.

For consistency with the Phase 1b/2a and Phase 2b trials, the same WHO scoring criteria was utilized to measure OM in ROMAN. FDA regulators preferred WHO, as unlike RTOG and CTCAEv5·0, WHO clarifies impact of OM on patient diet (solids, liquids, nothing by mouth). However, RTOG or CTCAEv5·0 could have been utilized in addition to the WHO scoring criteria for comparison. Villa et al. evaluated concordance of WHO, RTOG, and CTCAE scoring in a similar population of two hundred patients with OP/OC cancer receiving chemoradiation enrolled in a double-blind, randomized, placebo-controlled trial.^15^ Discordance was seen with patients exhibiting mild to moderate OM or most severe (grade 4) OM. When patients had severe OM (WHO score ≥3), 99·6% of CTCAE and 97·7% of RTOG scores were concordant.

**Fig. 4:**
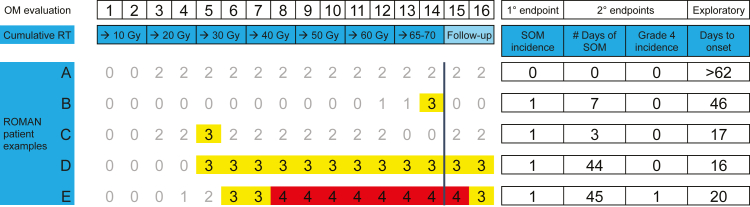
**Patient examples with different SOM burdens**. Gy, Gray; OM, oral mucositis; RT, radiation therapy; SOM, severe OM.

Assessment of patient burden and efficacy of an agent in reducing SOM requires considering multiple parameters in a “holistic” approach, rather than simply a single endpoint such as cumulative incidence through IMRT (Fig. 4). For example, in ROMAN, avasopasem SOM incidence bumped up around day 43. Post-hoc analysis suggests this late bump was mostly driven by patients who had an early dose reduction of avasopasem due to toxicity attributed to the study infusion. Per protocol, a 25% dose reduction of avasopasem/placebo was required for Grade 2+ hypotension within 2 h after the start of avasopasem/placebo infusion. Dose may also be reduced by 25% for Grade 3-4 adverse events judged by the Investigator to be likely attributable to the study infusion. Ten percent of avasopasem patients had one 25% dose reduction and 6% had two dose reductions (total 50% dose reduction), vs 5% and 1% in the placebo arm, respectively (Table 2). Grade 2+ hypotension was the most common reason for the first dose reduction and syncope was the most common grade 3 toxicity associated with dose reduction, overall occurring in 5% of the avasopasem arm vs 3% of placebo arm (Fig. 3, Appendix Table A3). Drug dose-reductions were more frequent in phase 3 than phase 2b. Further studies are required to assess AE management and appropriate avasopasem dose.

The limited sensitivity of incidence as a primary measure of efficacy potentially negates the true benefit of an intervention in that it fails to recognize that effective mitigation of SOM duration markedly impacts patients’ tolerance of their cancer treatment and its overall burden of illness. To equate efficacy between patients who had a single day of SOM vs those with weeks of OM is a fallacious comparison. Nonetheless, this trial was designed to assess efficacy based on a 34% difference in SOM incidence at the end of IMRT. For the reasons described above, however, we only observed 16% difference (*p* = 0·045). The design estimate was based on the greatest incidence reduction seen in the phase 2b–at the end of IMRT landmark. Importantly, comparing phase 2b vs ROMAN SOM incidence at other landmark doses of radiation delivery (30, 40, 50 Gy and 2 weeks following), the relative reductions were larger in ROMAN than in the phase 2b^11^ and supports a conclusion that avasopasem is beneficial throughout the IMRT course as well as its relevance in an era exploring de-escalation of elective radiation dose.^16^^,^^17^

Recognizing the holistic impact of SOM, an unplanned secondary analysis using a multiparametric statistical method called generalized pairwise comparisons (GPC) was performed to allow for simultaneous analysis of prioritized key clinically relevant outcomes from ROMAN: 1) WHO grade 4 OM incidence, 2) SOM incidence, 3) days of SOM, 4) days to SOM onset. Avasopasem showed statistically significant net benefit on all four key outcomes with a 53·9% probability that avasopasem would benefit patients vs a 35·0% probability that placebo would, translating to a net treatment benefit (NTB) of 18·9%, (95% CI 0·075, 0·303, *p* = 0·0012) and an avasopasem number needed to treat of 5·3 patients.^18^ The NTB reflects the net probability that a treated patient has a better outcome than a control patient, based on the predefined outcome hierarchy. While the FDA requires a primary endpoint such as incidence for drug approval, secondary analyses such an AUC or multiparametric analysis like GPC provides a more impactful and holistic outcome for patients than incidence, and future analysis and trial design should take this into consideration.

As in the phase 2b, avasopasem appeared well tolerated, with an AE profile similar to placebo. Superoxide reduction by avasopasem was anticipated to cause transient potentiation of NO, increasing AEs such as hypotension and episodes of syncope, as was evident in prior clinical trials.^19^, ^20^, ^21^ In ROMAN, grade 3+ hypotension was not increased with avasopasem, and the grade 1 or 2 hypotension or dizziness observed was of limited clinical consequence. Also possibly related to NO potentiation, a slight increase in grade 1 or 2, but not grade 3, nausea or vomiting was observed. This appeared limited and easily managed clinically. There was a small numerical imbalance in incidence of pulmonary embolism, which was not seen in phase 2b, and the incidence in both arms fell within published rates in this population.^11^^,^^22^ Phase 3 was conducted during COVID and we hypothesize this may have been a contributor. Further, nonclinical *in vivo* studies describe avasopasem as antithrombotic.^23^ We also hypothesize that COVID may have been a contributor to excess deaths on the avasopasem arm not attributed to study drug and not previously seen in Phase 2b, especially because several of the deaths were clustered at a known COVID hotspot and two of the deaths of unknown cause were 18–21 months after administration of the drug. However, 4 deaths occurred during the chemoRT and avasopasem infusion period. Principal Investigators with intimate knowledge of those 4 deaths did not attribute them to avasopasem. Available data cannot exclude avasopasem as a factor.

Importantly, adverse events associated with cisplatin appeared reduced, including multiple kidney injury markers. This trend to reduced acute nephrotoxicity was mirrored in prospectively defined renal function follow-up through 12 months showing avasopasem markedly reduced CKD. Nonclinical studies have shown superoxide's key role in cisplatin-induced kidney damage, as well as avasopasem's ability to prevent it, and retrospective assessments among subsets of patients in the phase 2b corroborated those animal findings.^24^, ^25^, ^26^ The results from ROMAN show avasopasem may be relevant to the care of patients with other cancers treated with cisplatin. Preliminary exploration of phase 2b data did not show benefit of avasopasem for tinnitus, xerostomia or trismus but the trial was not rigorously designed to assess these endpoints.^12^ It is hypothesis-generating that in ROMAN incidences of common RT-related side effects like dermatitis, xerostomia, dysgeusia, and tinnitus were nominally less frequent with avasopasem (Appendix Table A2).

This trial did not include a detailed assessment of resource utilization, but the data collected suggest that avasopasem may be associated with a reduced need for narcotics and gastrostomy tubes. While administration of avasopasem prior to RT involved logistical coordination and planning, delivery of avasopasem was achieved at nearly 70 study sites representing both academic and community centers. The increased resources and cost of avasopasem infusion prior to each RT fraction should be studied in relationship to patient burden and recovery in a future cost-effectiveness analysis. The drug's mechanism precludes administration by mouth or by subcutaneous injection. Recent bench research suggests avasopasem may persist in the mitochondria for several days, thus less frequent scheduling of administration of the drug is an area for further research.

Nonclinical studies support mechanism-based expectations that avasopasem does not reduce CRT antitumor effect, and show that, at higher fraction doses, avasopasem enhances RT efficacy.^27^^,^^28^ Reinforcing this, results from a placebo-controlled phase 1b/2 study demonstrated that avasopasem markedly improved tumor outcomes in locally advanced or borderline resectable pancreatic cancer treated with SBRT.^29^ Further, published 2-year tumor outcomes from the phase 2b HNC trial showed comparable Kaplan–Meier (K-M) estimates of 1- and 2-year OS, PFS, LRC, and DMFS between treatment arms and no statistically significant differences observed.^12^ In ROMAN, there were numerical differences in tumor outcomes at 1 year and overall survival at 2 years that appeared to favor placebo; however, CIs were all overlapping and outcomes in both arms (especially placebo) exceeded historical expectations. Additionally, the ROMAN population was a mixed prognosis combination of oral cavity and oropharynx patients, yet tumor outcomes and overall survival were on par with estimates from K-M curves for the HPV + oropharynx-only cisplatin arm of RTOG 1016 (1-year OS 97%, PFS 91%, LRC 95%, 2-year OS 92%).^30^ Since there was no previous statistical or experimental evidence of tumor-sparing with avasopasem on Phase Ib/2a nor Phase 2b trials, the Phase 3 ROMAN trial was not designed to stratify patients by tumor staging or HPV status. Additional stratification by AJCC stage and/or HPV status could be considered in future trial design.

Limitations to ROMAN, in addition to others discussed above, include absence of a separate patient-reported outcome (PRO) instrument. While PROs were considered during phase 3 design, the multitude of confounding factors impacting quality of life during HNC CRT drove the final decision. Even if avasopasem significantly decreased incidence and duration of SOM, individual patients lack a frame of reference and could still rate various quality of life metrics as the worst they have ever experienced in their life, not knowing how bad it could be in the placebo setting. This is evidenced by Oral Mucositis Daily Questionnaire (OMDQ) data collected for phase 2b, which did not show any discernible impact of 30 mg or 90 mg of avasopasem compared to placebo.^11^ Moreover, it should be noted that the ROMAN OM assessment was based on patient-reported inputs collected in a quality-controlled process, providing robust/consistent evaluation. However, lack of PRO was a lost opportunity to capture patient-level impact of the time required for daily 60-min IV infusion, drug-related side effects, and potential for earlier return to normal diet/improved quality of life at 1- and 3-month follow-up.

Use of the modified WHO scoring system also may confound comparison with other published results, although the trajectory of SOM noted in the control arm is consistent with those reported in numerous publications This system has been utilized and reported consistently through phase 1b/2a and 2b trials of avasopasem, with the technique designed to more clearly report avasopasem impact on SOM in the presence of confounding factors, and in a randomized trial, this modification should not bias the comparison between arms. Ideally, tumor outcomes (LRC, PFS and DDC) would have been collected through 2 years. ROMAN was also not designed to assess other long-term complications of HNC RT—notably, xerostomia and trismus. Finally, extending OM follow-up more than two weeks after RT would have provided more detail about SOM duration and resolution.

The COVID pandemic effected OM assessment and data collection on this trial due to concern for potential disease transmission during close examination of the mouth in the setting of limited initial understanding of COVID transmission and limited availability of personal protective equipment. COVID may also have been a contributing factor to cardiac and thromboembolic events and non-oncologic deaths not previously seen in phase 2b, as several of these patients were clustered at a COVID hotspot at the onset of the pandemic. While unlikely based on previous data, we cannot exclude the possibility that avasopasem was a contributing factor to these adverse events.

In August 2023, the Food and Drug Administration (FDA) concluded its review of avasopasem and ultimately requested a confirmatory phase 3 trial for several reasons including lower than predicted reduction in incidence of SOM at the primary endpoint and missing follow-up data that decreased the strength of the duration endpoint analysis. The distribution of early-stage/better prognosis patients was slightly higher in the avasopasem arm (Appendix Table A1), yet tumor outcomes were numerically (though not statistically) better in the placebo arm. There was also a numerically higher incidence of thromboembolic events, major adverse cardiac events, and deaths observed in the avasopasem arm. Therefore, ROMAN did not provide a sufficiently favorable benefit-risk determination for approval.

In the ROMAN phase 3 study, avasopasem produced significant improvement in SOM vs placebo that was consistent across multiple measures, however the primary endpoint of incidence was not as improved as predicted by the Phase 2b trial and avasopasem's contribution to adverse events could not be excluded. For these reasons and others discussed, ROMAN did not provide a sufficiently favorable benefit-risk determination for FDA approval. A confirmatory phase 3 trial was requested.

## Contributors

All authors read and approved the final version of the manuscript. Carryn Anderson, Stephen T. Sonis, Jon Holmlund, Robert A. Beardsley, Eugene P Kennedy, and Deborah Saunders had access to and verified the underlying data.

**Concept and design:** CA, JH, RAB, DS.

**Acquisition of the data:** CA, JK, GW, ND, VBA, DM, VK, AP, DC, FV, BM, AGT, STS, DS.

**Data analysis and interpretation:** CA, VK, FV, CML, STS, JH, RA B, EPK, DS.

**Writing:** CA, JH, RAB, EPK, STS.

**Provided critical review and final approval to submit:** All co-authors.

**Accountable for all aspects of the work:** All co-authors.

## Data sharing statement

The institutional review board approvals for this study did not include a data sharing plan and therefore data from the study will not be shared publicly.

## Declaration of interests

**Carryn Anderson** was an uncompensated consultant for Galera October 2013–August 2024; from June 30, 2023 to August 10, 2023 she received consulting fees from Galera for contributions to development of provider educational materials; her institution received research funding from Galera to conduct the study and the institution was reimbursed travel/attendance costs to present ROMAN data at meetings; Galera provided study materials, paid for editorial support for the manuscript through AOIC (see acknowledgements), assisted in data analysis, data interpretation, editing the manuscript and paid article processing charges; her institution received research funding from Soligenix; **Christopher M. Lee** provided consultancy for Bayer, Myriad Genetics and Elekta; received honoraria from Eli Lilly, Bayer, Merck, and Bristol-Myers Squibb; participated in speaker bureaus for Eli Lilly, Bayer, Merck, Myriad Genetics and Bristol-Myers Squibb; his institution received research funding from Galera to conduct this study; he holds patents or receives royalties from Kobold Medical LLC, Axcend LLC, and Gamma Knife Spokane; he has received travel, accommodations, and expenses from Eli Lilly, Bayer, Merck, Elekta, Myriad Genetics and Bristol-Myers Squibb; and he holds equity in Kobold Medical LLC and Axcend LLC.

**Joseph Kelley's** institution received research funding from Galera to conduct this study.

**Gary Walker's** institution received research funding from Galera to conduct the study and is a past advisory board member of Galera.

**Neal Dunlap's** institution received research funding from Galera to conduct the study.

**Voichita Bar-Ad's** institution received research funding from Galera to conduct the study.

**Douglas Miller's** institution received research funding from Galera to conduct this study.

**Vernon King's** institution received research funding from Galera to conduct the study and he holds equity in Galera, Novocure, and Ensign Group.

**Abhinand Peddada's** institution received research funding from Galera to conduct this study and he previously held stock in Galera.

**Douglas Ciuba**'s institution received research funding from Galera to conduct the study.

**Francois Vincent** provided consultancy for Bayer; his institution received research funding from Bayer, Sanofi, Merck and Galera to conduct this study; and he holds equity in CVS Health, Merck, AstraZeneca, Smith & Nephew, United Health Group, and Medtronic.

**Brian Muzyka**'s institution received research funding from Galera to conduct this study.

**Amanda Gillespie-Twardy**'s institution received research funding from Galera to conduct the study.

**Stephen T. Sonis** Dr. Sonis is a full-time employee of Biomodels, LLC and Primary Endpoint Solutions, LLC. As an employee of Biomodels and PES, he is involved in assisting industry (including Galera Therapeutics), government and academics in the study and enablement of drugs, biologicals and devices to treat patients for a broad range of indications including cancer regimen-related toxicities such oral mucositis. He does not have equity or receive payment from clients of either company.

**Jon Holmlund** was an employee of Galera during the primary conduct and analysis of this trial, receiving cash and stock compensation. He received travel and support for attending meetings as an employee. He previously held stock/stock options in Galera at the time of study.

**Robert A. Beardsley** was an employee and consultant of Galera during the primary conduct and analysis of this trial, is an inventor on patents related to avasopasem and use in the setting of this study and holds stock in Galera.

**Eugene P. Kennedy** was an employee of Galera during the primary conduct and analysis of this trial and held stock/stock options in Galera at the time of study.

**Deborah Saunders**’ institution received research funding from Galera to conduct the study and helds stock in Galera during the time of study but no long holds stock.

## Appendix A Supplementary data

Supplementary Figures and Tables

Redacted Protocol
