# Supplementary material for: Avasopasem manganese treatment for severe oral mucositis from chemoradiotherapy for locally advanced head and neck cancer: phase 3 randomized controlled trial (ROMAN)
Source: eClinicalMedicine. 2025 Oct 8;89:103539. doi: 10.1016/j.eclinm.2025.103539 (PMC12538942; doi:10.1016/j.eclinm.2025.103539)
Supplement: Supplementary Figures and Tables [file mmc1.docx]

Appendix, supplementary material

Contents: Page:

Figure A1 1

Figure A2 2

Table A1 3

Table A2 4

Table A3 6

Table A4 9

Participating Institutions with Ethics Approval Numbers 12

**Figure A1. Time to SOM Onset**


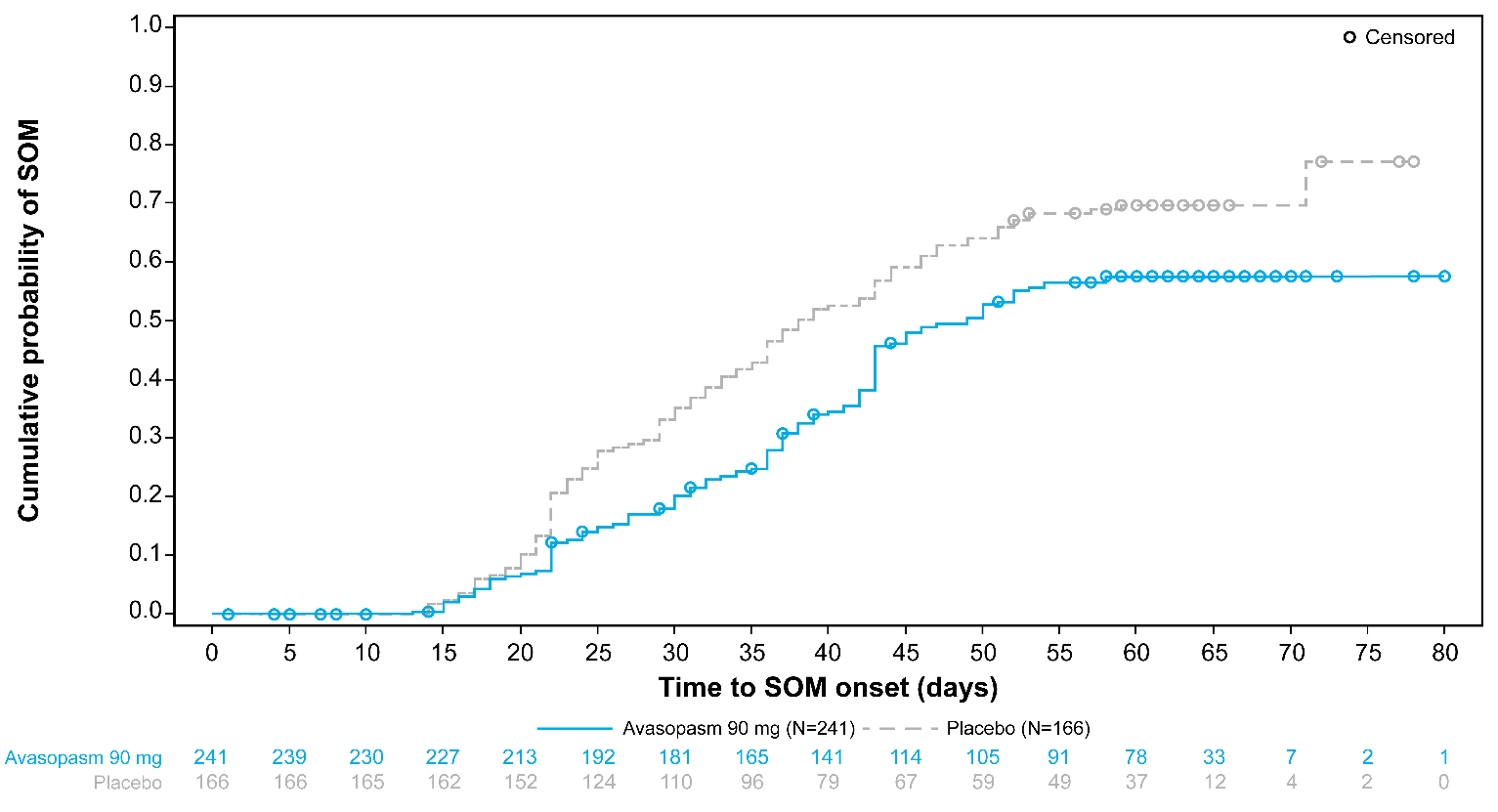


SOM, severe oral mucositis.

**Figure A2. SOM Incidence and Duration by Subpopulation. Figure A2A: Relative Risk of SOM Incidence Through End of IMRT; Figure A2B: Least Square Means SOM Duration Through 2 Weeks Post-IMRT.**


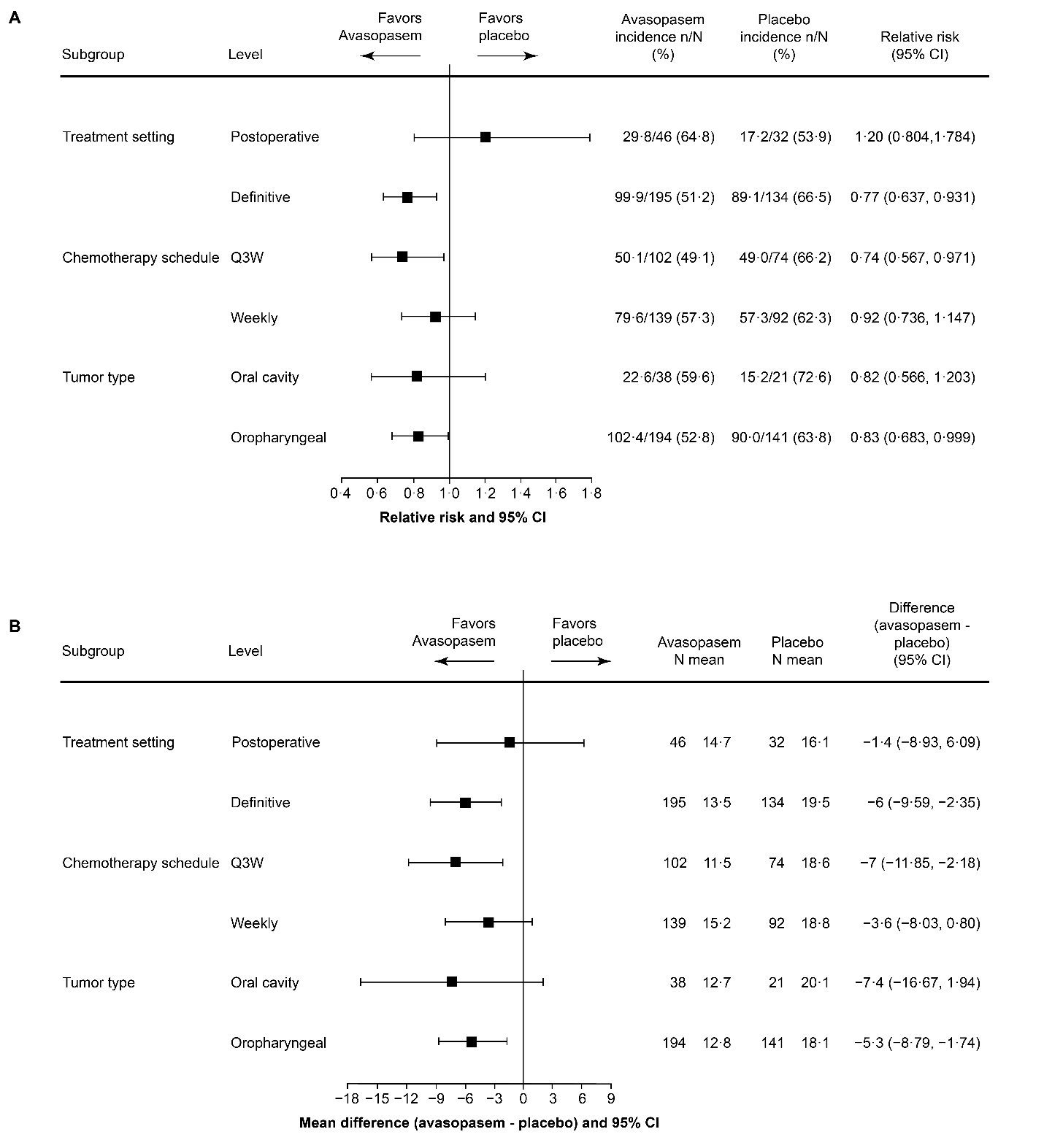


CI, confidence interval; IMRT, intensity-modulated radiation therapy; Q3W, every 3 weeks; SOM, severe oral mucositis. There were no preplanned tests of interaction. As a post hoc analysis, there is evidence of a significant treatment by Setting (post operative/definitive) for the incidence of SOM (p=0·04, Breslow-Day test of homogeneity of odds ratios from the CMH Chi-square test stratified by Setting using the average incidence of all 50 imputations).

SOM Duration interaction was assessed by an ANOVA Model with terms for Treatment, Setting, Chemo Schedule and the Treatment x Setting interaction. Each of the 50 separate analyses on the 50 separate imputation datasets had P>0·1 for the test of interaction. The average interaction of all N=50 tests was p=0·27. In addition to being unplanned, the study was not powered to test for interactions and therefore lack of significant interaction (in this case defined post hoc to be at the 0·1 level) can be due to low power.

Table A1. TNM Stage by Tumor Site^ab^

|  | **AVA 90 mg**  **(n=241)** | **PBO**  **(n=166)** | **All patients**  **(N=407)** | **P-Value** |
| --- | --- | --- | --- | --- |
| Tumor site, n (%) |  |  |  |  |
| Oropharyngeal | 194 (80) | 141 (85) | 335 (82) |  |
| HPV status |  |  |  |  |
| Positive | 175 (92) | 123 (89) | 298 (91) | 0·45 |
| Negative | 16 (8) | 15 (11) | 31 (9) |  |
| Unknown | 3 | 3 | 6 |  |
| HPV p16+ staging, n (%) | | | |  |
| I | 76 (43) | 37 (30) | 113 (38) | **0·04** |
| II | 64 (37) | 61 (50) | 125 (42) |  |
| III | 35 (20) | 25 (20) | 60 (20) |  |
| HPV p16− staging, n (%) | | | |  |
| II | 1 (7) | 1 (7) | 2 (6) | 1·00 |
| III | 4 (27) | 3 (20) | 7 (23) |  |
| IVA | 8 (53) | 9 (60) | 17 (55) |  |
| IVB | 2 (13) | 2 (13) | 4 (13) |  |
| Unknown | 1 | 0 | 1 |  |
| Unknown HPV type staging, n (%) | | | |  |
| I | 1 (33) | 1 (33) | 2 (33) |  |
| III | 2 (67) | 1 (33) | 3 (50) |  |
| IVA | 0 | 1 (33) | 1 (17) |  |
| Oral cavity | 38 (16) | 21 (13) | 59 (14) |  |
| HPV status, n (%) | | | |  |
| Positive | 13 (34) | 8 (42) | 21 (37) | 0·56 |
| Negative | 25 (66) | 11 (58) | 36 (63) |  |
| Unknown | 0 | 2 | 2 |  |
| TNM stage, n (%) | | | |  |
| I | 2 (5) | 4 (19) | 6 (10) | 0·60 |
| II | 5 (13) | 3 (14) | 8 (14) |  |
| III | 9 (24) | 4 (19) | 13 (22) |  |
| IVA | 16 (42) | 7 (33) | 23 (39) |  |
| IVB | 6 (16) | 3 (14) | 9 (15) |  |
| Unknown primary site | 9 (4) | 4 (2) | 13 (3) |  |
| HPV status, n (%) | | | |  |
| Positive | 5 (63) | 3 (75) | 8 (67) | 1·00 |
| Negative | 3 (37) | 1 (25) | 4 (33) |  |
| Unknown | 1 | 0 | 1 |  |
| TNM stage, n (%) | | | |  |
| II | 2 (22) | 2 (50) | 4 (31) | 0·78 |
| III | 3 (33) | 1 (25) | 4 (31) |  |
| IVA | 4 (44) | 1 (25) | 5 (38) |  |

AVA, avasopasem Mn; HPV, human papillomavirus; PBO, placebo; TNM, tumor, node, metastasis.

^a^Because of rounding, percentages may not total 100.

^b^According to American Joint Committee on Cancer, 8^th^ Edition.

Table A2. Adverse Events Experienced by ≥10% of Patients in Either Arm

| **Adverse events, n, %** | **AVA 90 mg**  **(N=241)** | **PBO**  **(N=166)** |
| --- | --- | --- |
| Total No. Of TEAEs | 6779 | 4937 |
| Any TEAEs | 240 (>99) | 166 (100) |
| TEAE by preferred term |  |  |
| Lymphopenia | 212 (88) | 155 (93) |
| Nausea | 207 (86) | 137 (83) |
| Fatigue | 170 (71) | 124 (75) |
| Oropharyngeal pain | 146 (61) | 113 (68) |
| Constipation | 155 (64) | 102 (61) |
| Dysgeusia | 137 (57) | 119 (72) |
| Dry mouth | 129 (54) | 104 (63) |
| Radiation skin injury | 126 (52) | 104 (63) |
| Vomiting | 136 (56) | 78 (47) |
| Weight decreased | 116 (48) | 81 (49) |
| Leukopenia | 103 (43) | 85 (51) |
| Dysphagia | 107 (44) | 71 (43) |
| Dehydration | 79 (33) | 70 (42) |
| Decreased appetite | 81 (34) | 61 (37) |
| Neutropenia | 79 (33) | 60 (36) |
| Headache | 74 (31) | 57 (34) |
| Tinnitus | 69 (29) | 62 (37) |
| Oral candidiasis | 74 (31) | 46 (28) |
| Diarrhea | 52 (22) | 57 (34) |
| Dizziness | 68 (28) | 39 (23) |
| Hiccups | 70 (29) | 37 (22) |
| Hypomagnesemia | 48 (20) | 54 (33) |
| Hypokalemia | 63 (26) | 32 (19) |
| Dyspepsia | 54 (22) | 35 (21) |
| Hypotension | 56 (23) | 32 (19) |
| Dysphonia | 47 (20) | 40 (24) |
| Anemia | 41 (17) | 36 (22) |
| Blood creatinine increased | 39 (16) | 35 (21) |
| Insomnia | 39 (16) | 34 (20) |
| Cough | 36 (15) | 36 (22) |
| Pyrexia | 36 (15) | 28 (17) |
| Candida infection | 35 (15) | 27 (16) |
| Gastroesophageal reflux disease | 40 (17) | 20 (12) |
| Odynophagia | 36 (15) | 22 (13) |
| Saliva altered | 32 (13) | 22 (13) |
| Thrombocytopenia | 27 (11) | 25 (15) |
| Paresthesia | 36 (15) | 15 (9) |
| Chills | 16 (7) | 23 (14) |
| Hypertension | 21 (9) | 18 (11) |
| Tachycardia | 26 (11) | 9 (5) |
| Flushing | 25 (10) | 9 (5) |
| Pruritus | 17 (7) | 16 (10) |
| Dry skin | 15 (6) | 17 (10) |
| Productive cough | 12 (5) | 16 (10) |
| Rash maculo-papular | 10 (4) | 16 (10) |

AVA, avasopasem Mn; PBO, placebo; TEAE, treatment-emergent adverse event.

Table A3. Adverse Events of Grade 3 or Greater Experienced by Three or More Patients (≥1%)

| **Adverse events, n %** |  | **AVA 90 mg**  **(N=241)** | **PBO**  **(N=166)** |
| --- | --- | --- | --- |
| Total No. of grade ≥3 TEAEs |  | 1160 | 920 |
| Any grade ≥3 TEAE |  | 225 (93) | 163 (98) |
| TEAE by preferred term |  |  |  |
| Lymphopenia |  | 212 (88) | 155 (93) |
| Leukopenia |  | 99 (41) | 77 (46) |
| Neutropenia |  | 67 (28) | 48 (29) |
| Dysphagia |  | 29 (12) | 25 (15) |
| Oropharyngeal pain |  | 24 (10) | 28 (17) |
| Anemia |  | 26 (11) | 24 (14) |
| Decreased appetite |  | 25 (10) | 24 (14) |
| Nausea |  | 26 (11) | 21 (13) |
| Hypokalemia |  | 14 (6) | 14 (8) |
| Fatigue |  | 12 (5) | 14 (8) |
| Febrile neutropenia |  | 14 (6) | 8 (5) |
| Dehydration |  | 12 (5) | 9 (5) |
| Hypertension |  | 11 (5) | 10 (6) |
| Radiation skin injury |  | 13 (5) | 8 (5) |
| Syncope |  | 13 (5) | 5 (3) |
| Vomiting |  | 8 (3) | 10 (6) |
| Weight decreased |  | 10 (4) | 8 (5) |
| Hypomagnesemia |  | 8 (3) | 9 (5) |
| Alanine aminotransferase increased |  | 8 (3) | 6 (4) |
| Hypotension |  | 8 (3) | 6 (4) |
| Thrombocytopenia |  | 8 (3) | 6 (4) |
| Dry mouth |  | 7 (3) | 5 (3) |
| Pulmonary embolism |  | 8 (3) | 2 (1) |
| Sepsis |  | 6 (2) | 4 (2) |
| Failure to thrive |  | 3 (1) | 6 (4) |
| Acute kidney injury |  | 2 (1) | 6 (4) |
| Hypocalcemia |  | 3 (1) | 5 (3) |
| Hyponatremia |  | 5 (2) | 3 (2) |
| Hypoxia |  | 5 (2) | 3 (2) |
| Esophagitis |  | 4 (2) | 4 (2) |
| Pneumonia |  | 6 (2) | 2 (1) |
| Blood creatinine increased |  | 3 (1) | 4 (2) |
| Hypoglycemia |  | 2 (1) | 4 (2) |
| Hypophosphatemia |  | 5 (2) | 1 (1) |
| Saliva altered |  | 5 (2) | 1 (1) |
| Atrial fibrillation |  | 2 (1) | 3 (2) |
| Constipation |  | 3 (1) | 2 (1) |
| Diarrhea |  | 3 (1) | 2 (1) |
| Aspartate aminotransferase increased |  | 2 (1) | 2 (1) |
| Deep vein thrombosis |  | 3 (1) | 1 (1) |
| Dysphonia |  | 1 (<1) | 3 (2) |
| Hyperkalemia |  | 2 (1) | 2 (1) |
| Lung infection |  | 2 (1) | 2 (1) |
| Malnutrition |  | 3 (1) | 1 (1) |
| Odynophagia |  | 1 (<1) | 3 (2) |
| Pneumonia aspiration |  | 2 (1) | 2 (1) |
| Tinnitus |  | 2 (1) | 2 (1) |
| Acute respiratory failure |  | 1 (<1) | 2 (1) |
| Asthenia |  | 1 (<1) | 2 (1) |
| Cardiac failure congestive |  | 2 (1) | 1 (1) |
| Chronic kidney disease |  | 0 (0) | 3 (2) |
| Device malfunction |  | 3 (1) | 0 (0) |
| Device-related infection |  | 2 (1) | 1 (1) |
| Dizziness |  | 3 (1) | 0 (0) |
| Dyspnea |  | 3 (1) | 0 (0) |
| Embolism |  | 2 (1) | 1 (1) |
| Glossodynia |  | 1 (<1) | 2 (1) |
| Hypernatremia |  | 3 (1) | 0 (0) |
| Leukocytosis |  | 2 (1) | 1 (1) |
| Noncardiac chest pain |  | 1 (<1) | 2 (1) |
| Pyrexia |  | 2 (1) | 1 (1) |
| Salivary duct inflammation |  | 3 (1) | 0 (0) |
| Urinary tract infection |  | 2 (1) | 1 (1) |

AVA, avasopasem Mn; PBO, placebo; TEAE, treatment-emergent adverse event.

Table A4. Serious Adverse Events

| **Serious adverse events, n, %** | **AVA 90 mg**  **(N=241)** | **PBO**  **(N=166)** |
| --- | --- | --- |
| Total No. of SAEs | 124 | 85 |
| Patients with any SAE | 81 (34) | 52 (31) |
| SAE by preferred term |  |  |
| Febrile neutropenia | 15 (6) | 8 (5) |
| Dehydration | 7 (3) | 3 (2) |
| Vomiting | 4 (2) | 6 (4) |
| Failure to thrive | 3 (1) | 6 (4) |
| Pulmonary embolism | 7 (3) | 2 (1) |
| Sepsis | 4 (2) | 4 (2) |
| Dysphagia | 3 (1) | 4 (2) |
| Nausea | 5 (2) | 2 (1) |
| Acute kidney injury | 1 (<1) | 5 (3) |
| Pyrexia | 6 (2) | 0 (0) |
| Lung infection | 2 (1) | 2 (1) |
| Pneumonia | 3 (1) | 1 (1) |
| Asthenia | 1 (<1) | 2 (1) |
| Atrial fibrillation | 1 (<1) | 2 (1) |
| Decreased appetite | 2 (1) | 1 (1) |
| Deep vein thrombosis | 2 (1) | 1 (1) |
| Device malfunction | 3 (1) | 0 (0) |
| Hypotension | 3 (1) | 0 (0) |
| Hypoxia | 2 (1) | 1 (1) |
| Malnutrition | 2 (1) | 1 (1) |
| Pancreatitis | 1 (<1) | 2 (1) |
| Pneumonia aspiration | 2 (1) | 1 (1) |
| Cardiac failure congestive | 2 (1) | 0 (0) |
| Constipation | 2 (1) | 0 (0) |
| Hepatitis viral | 2 (1) | 0 (0) |
| Hypokalemia | 0 (0) | 2 (1) |
| Noncardiac chest pain | 1 (<1) | 1 (1) |
| Odynophagia | 1 (<1) | 1 (1) |
| Weight decreased | 0 (0) | 2 (1) |
| Abdominal wall abscess | 1 (<1) | 0 (0) |
| Accidental overdose | 1 (<1) | 0 (0) |
| Acute myocardial infarction | 1 (<1) | 0 (0) |
| Acute respiratory failure | 0 (0) | 1 (1) |
| Bacteremia | 0 (0) | 1 (1) |
| Bronchitis | 1 (<1) | 0 (0) |
| Cardiac arrest | 1 (<1) | 0 (0) |
| Cardio-respiratory arrest | 1 (<1) | 0 (0) |
| Cerebrovascular accident | 1 (<1) | 0 (0) |
| Cholecystitis acute | 0 (0) | 1 (1) |
| Corona virus infection | 0 (0) | 1 (1) |
| Death | 1 (<1) | 0 (0) |
| Delirium | 1 (<1) | 0 (0) |
| Depression | 1 (<1) | 0 (0) |
| Device occlusion | 1 (<1) | 0 (0) |
| Device-related infection | 0 (0) | 1 (1) |
| Diarrhea | 1 (<1) | 0 (0) |
| Euglycemic diabetic ketoacidosis | 0 (0) | 1 (1) |
| Gastrostomy tube removal | 1 (<1) | 0 (0) |
| Gastrostomy tube site complication | 1 (<1) | 0 (0) |
| Hematemesis | 0 (0) | 1 (1) |
| Hemorrhoidal hemorrhage | 1 (<1) | 0 (0) |
| Hypercoagulation | 0 (0) | 1 (1) |
| Hypernatremia | 1 (<1) | 0 (0) |
| Hypoglycemia | 1 (<1) | 0 (0) |
| Hypomagnesemia | 0 (0) | 1 (1) |
| Hyponatremia | 0 (0) | 1 (1) |
| Hypovolemia | 1 (<1) | 0 (0) |
| Laryngeal edema | 0 (0) | 1 (1) |
| Lipase increased | 1 (<1) | 0 (0) |
| Metastases to peritoneum | 1 (<1) | 0 (0) |
| Muscle hemorrhage | 0 (0) | 1 (1) |
| Nephrolithiasis | 1 (<1) | 0 (0) |
| Neutropenic sepsis | 1 (<1) | 0 (0) |
| Oropharyngeal candidiasis | 1 (<1) | 0 (0) |
| Parotitis | 1 (<1) | 0 (0) |
| Presyncope | 1 (<1) | 0 (0) |
| Sinus bradycardia | 1 (<1) | 0 (0) |
| Skin ulcer | 1 (<1) | 0 (0) |
| Staphylococcal sepsis | 0 (0) | 1 (1) |
| Stoma site infection | 1 (<1) | 0 (0) |
| Syncope | 0 (0) | 1 (1) |
| Tremor | 0 (0) | 1 (1) |
| Tumor hemorrhage | 1 (<1) | 0 (0) |
| Upper gastrointestinal hemorrhage | 0 (0) | 1 (1) |
| Urinary tract infection | 0 (0) | 1 (1) |

AVA, avasopasem Mn; PBO, placebo; SAE, serious adverse event.

**Participating Institutions with Approval Numbers from Ethics Committees**

| **Site#** | **IRB Tracking #** | **Principal Investigator** | **Site** | **Ethics Committee** |
| --- | --- | --- | --- | --- |
| 002 | 20181417 | William Wisbeck, MD | Providence Regional Cancer Center | WIRB |
| 003 | 20181417 | Amarinthia Curtis, MD | Spartanburg Regional Medical Center | WIRB |
| 004 | 00002126 | Madhavi L. Venigalla, MD (Haider) | Lakeland Regional Health System | Lakeland Regional Health IRB |
| 006 | 20181417 | James H Wheeler, MD, PhD | Indiana University Health Goshen Center for Cancer Care | WIRB |
| 010 | SLUHN 2018-97 | Sanjiv Agarwala, M. D. (Lu) | St. Luke's University Health | St. Luke’s University Health Network IRB |
| 011 | 20181417 | Carryn M. Anderson, MD | University of Iowa Healthcare | WIRB |
| 014 | IRB00053295 | Mercedes Porosnicu, MD | Wake Forest Baptist Health | Wake Forest University Health Services IRB |
| 015 | 20181417 | Christopher Lee, MD and Robert Fairbanks, MD | Cancer Care Northwest | WIRB |
| 017 | 20181417 | Voichita Bar-Ad, MD | Sidney Kimmel Cancer Center | WIRB |
| 018 | 20181417 | Joseph Kelley, MD and Neil Faulkner, MD | University of TN Medical Center | WIRB |
| 019 | IRB00012211 | Francis Worden, MD | University of Michigan Medical School | IRBMED |
| 023 | 1752 Ref#000411 | Steve P Lee, MD | VA Long Beach Healthcare | VA Long Beach Healthcare System IRB |
| 024 | 2019-037 | Kala Seetharaman, MD | MetroWest Medical Center | MetroWest Medical Center IRB |
| 026 | 0818.19f | Kyle T. Colvett, MD | East Tennessee State University | E. Tennessee State University IRB |
| 028 | 20181417 | Michael Trendle, MD and Puja Nistala, MD | University of Missouri | WIRB |
| 033 | 20181417 | Philip Kovoor, MD | Texas Oncology - Plano West | WIRB |
| 044 | 18.1098 | Neal E. Dunlap, MD | University of Louisville | University of Louisville IRB |
| 046 | 20181417 | Mauricio Gamez, MD | The Ohio State University | WIRB |
| 047 | 04541 | Daniel Clayburgh, MD | VA Portland Health Care System | VA Portland |
| 054 | 20181417 | Kevin Basil Collins, MD, JD | Fowler Family Center for Cancer Care | WIRB |
| 057 | 20181417 | Vernon King, MD | St. Mary's Hospital and Medical Center | WIRB |
| 058 | STU00209193 | Mark Agulnik, MD and Yanis Boumber, MD | Northwestern University | Northwestern University IRB |
| 059 | 20181417 | Patrick Wayne Cobb, MD | Vincent Healthcare | WIRB |
| 060 | 20181417 | Waqas Rehman, MD | Hunterdon Hematology-Oncology LLC | WIRB |
| 062 | 20181417 | Douglas Miller, MD | Jersey Shore University Medical | WIRB |
| 064 | 20181417 | Leander Cannick, MD | AnMed Health | WIRB |
| 066 | 20181417 | Abhinand Peddada, MD and Garrett Green, MD | Renown Regional Medical Center | WIRB |
| 067 | 20181417 | Ganesh Kudva, MD | Henry Ford Allegiance Health | WIRB |
| 068 | 18-067 | Deborah Saunders, DDS | Health Science North | Health Sciences North IRB |
| 070 | 20181417 | Charles S. Holladay, MD | Charleston Cancer Center | WIRB |
| 072 | CÉRM-2018-005 | François Vincent, MD | CHAUR, Quebec | Comite d'ethique de la recherche volet medical du CIUSSS MCQ |
| 073 | 20181417 | John Paul Thropay, MD | Clinical Trial Research Associates, Inc | WIRB |
| 076 | HC6-24-c218245 | Khalil Sultanem, MD | Sir Mortimer B Davis Jewish General Hospital | Comite d'ethique de la recherche volet medical du CIUSSS MCQ |
| 077 | 20181417 | Thomas Galloway, MD | Fox Chase Cancer Center | WIRB |
| 078 | GTI-4419-301 Amend 3: 27 November 2018 | Panagiotis Fidias, MD | Exeter Hospital | Exeter Hospital IRB |
| 079 | 20181417 | Mohammed Almubarak, MD | West Virginia University Cancer Institute | WIRB |
| 080 | 7H-18-1 | Bing Xia, MD | University of Southern California | Univ of Southern California IRB |
| 081 | UMCIRB 18-001258 | Brian Muzyka, MD | East Carolina University | East Carolina University IRB |
| 082 | 20181417 | Robert D. Siegel, MD, FACP | St. Francis Hospital, Inc. | WIRB |
| 083 | 20181417 | Douglas Ciuba, MD | IACT Health | WIRB |
| 084 | 20181417 | Gary Walker, MD | Banner MD Anderson Cancer Center | WIRB |
| 087 | 20181417 | Tina Khair, DO | Gettysburg Cancer Center | WIRB |
| 088 | 5876 | Brandon Meyers, MD | Hamilton Health Science Corporation | Hamilton Integrated Research Ethics Board |
| 090 | 018-758 | Christal Murray, MD | Baylor Scott & White Research Institute | Baylor Scott & White Research Institute IRB |
| 091 | 20181417 | Eddie Thara, DO | Innovative Clinical Research Institute | WIRB |
| 093 | 1812902 | Steven Kossman, MD | Sharp Memorial Hospital | Sharp HealthCare IRB |
| 094 | MUHC 2019-05-30 | George Shenouda, MD | McGill University Health Center-Cedar Cancer Center | Comite d'ethique de la recherche volet medical du CIUSSS MCQ |
| 095 | CÉRM-2018-005 | Phuc Felix Nguyen-Tan, MD | Centre Hospitalier de l 'Université de Montréal | Comite d'ethique de la recherche volet medical du CIUSSS MCQ |
| 097 | 20181417 | Ronald G. Maggiore, MD | Wilmot Cancer Institute | WIRB |
| 098 | H19-00295 | Sarah Hamilton, MD | BC Cancer Agency | UBC BC Cancer Research Ethics Board |
| 099 | CÉRM-2018-005 | Mathieu Leclerc, MD | CHU de Québec | Comite d'ethique de la recherche volet medical du CIUSSS MCQ |
| 100 | 20181417 | Zulfiqar A. Malik, MD, PhD | New York Cancer and Blood Specialists | WIRB |
| 101 | 20181417 | John Stewart Hrom, III, MD | Hattiesburg Clinic, PA | WIRB |
| 102 | 20181417 | Byron C. May, M.D. | Mayo Clinic Jacksonville | WIRB |
| 103 | SITE0000983 Study20191397 | Min Yao, MD and Jennifer Dorth | University Hospitals Cleveland MC | Advarra IRB |
| 104 | 20181417 | Jaspreet Singh Grewal, MD | Norton Healthcare | WIRB |
| 105 | CÉRM-2018-005 | Nader Khaouam, MD | Hôpital Maisonneuve-Rosemont | Comite d'ethique de la recherche volet medical du CIUSSS MCQ |
| 106 | GTI-4419-301 28-Feb-2019 | William Schulz, MD | Swedish American Regional Cancer Center | Swedish American Health System IRB |
| 107 | 20181417 | Ibrahim Abdalla, MD | Lester E Cox Medical Center | WIRB |
| 108 | 20181417 | Samuel Bailey, MD | Appalachian Regonal Healthcare | WIRB |
| 109 | 20181417 | Steven Joseph DiBiase, MD and Andrew Brandmaier | New York Presbyterian Queens | WIRB |
| 110 | VAM-19-00370 | Mark Klein, MD | Minneapolis VA Health Care System | Dept of Veterans Affairs IRB |
| 111 | 20181417 | Tracy W. Dobbs, MD | Tennesee Cancer Specialists | WIRB |
| 112 | 20181417 | Sonia Reichert, MD | Woodland Clinic | WIRB |
| 113 | 20181417 | Doug Shin, MD | Emory University | WIRB |
| 114 | 20181417 | Gopichand Pendurti, MD | Mosaic Life Care | WIRB |
| 116 | 20181417 | Pramvir S. Verma, MD | Fort Belvoir Community Hospital | WIRB |
| 117 | 916235 | Wilfred P. dela Cruz, MD PhD | San Antonio Military Medical Center | Regional Health Command-Central IRB |
| 118 | 20181417 | Brian S. Kim, MD | Hoag Memorial Presbyterian Hospital | WIRB |
| 119 | 20181417 | Michael E. Kasper, MD | Boca Raton Regional Hospital | WIRB |
| 120 | 20181417 | Christopher Spencer, MD | Delbert Day Cancer Institute | WIRB |
| 121 | 02993 | Ryan Burri, MD | Bay Pines VA Healthcare System | Bay Pines VA Health System IRB |
| 122 | 20181417 | Sarada Gurubhagavatula, MD | Summit Medical Group | WIRB |
| 123 | 20181417 | Brian Choi, MD | Compassionate Care Research Group, Inc. | WIRB |
| 124 | 20181417 | Grant W. Harrer, MD | Benefis Hospitals, Inc. | WIRB |
| 126 | 20181417 | Zachary Zumsteg, MD | Cedars-Sinai Medical Center | WIRB |
| 127 | 20181417 | Jason Eli Tache, DO | BRCR Medical Center, Inc | WIRB |
| 130 | 20181417 | Yaser Homsi, MD | Innovative Clinical Research Institute | WIRB |
| 132 | 20181417 | Richard John Cassidy, MD | Cancer Specialists of North Florida | WIRB |
| 134 | 20181417 | Chaitali Nangia, MD | Immuno-Oncology Clinic, Inc. | WIRB |
| 135 | CÉRM-2018-005 | Marc-André Brassard, MD | Chicoutimi Hospital | Comite d'ethique de la recherche volet medical du CIUSSS MCQ |
| 136 | 20181417 | Mayra Rivera-Figueroa, MD | Clinical Research Puerto Rico, Inc | WIRB |
| 153 | 20181417 | Brian Chang, MD | Parkview Research Center | WIRB |
| 154 | GTI-4419-301 5 Nov 2020 | Anand Desai, MD | Summa Health | Advarra IRB |
| 156 | 20181417 | David Crockett, MD | Commonspirit Health Research Institute | WIRB |
| 138 - US Oncology | 18182 | Nurul A. Wahid, MD | US Oncology Research | US Oncology IRB |
| 147 - US Oncology | 18182 | Anand Shivnani, MD | US Oncology Research | US Oncology IRB |
| 142 - US Oncology | 18182 | Ragene R. Rivera, MD | US Oncology Research | US Oncology IRB |
| 144 - US Oncology | 18182 | Eric S. Nadler, MD | US Oncology Research | US Oncology IRB |
| 141 - US Oncology | 18182 | Jeffery D. Morton, MD | US Oncology Research | US Oncology IRB |
| 146 - US Oncology | 18182 | Kristi J. McIntyre, MD | US Oncology Research | US Oncology IRB |
| 148 - US Oncology | 18182 | Vivek S. Kavadi, MD | US Oncology Research | US Oncology IRB |
| 137 - US Oncology | 18182 | Justin J. Juliano, MD | US Oncology Research | US Oncology IRB |
| 145 - US Oncology | 18182 | Amanda L. Gillespie-Twardy, MD | US Oncology Research | US Oncology IRB |
| 140 - US Oncology | 18182 | Carlos A. Encarnacion, MD | US Oncology Research | US Oncology IRB |
| 139 - US Oncology | 18182 | Emily F. Dunn, MD | US Oncology Research | US Oncology IRB |
| 143 - US Oncology | 18182 | L. Andy Chen, MD, PhD | US Oncology Research | US Oncology IRB |

WIRB = Western Institutional Review Board

IRB = Institutional Review Board

IRBMED = Institutional Review Boards of the University of Michigan Medical School

VA= Veterans Affairs
