# Supplementary material for: Avasopasem manganese treatment for severe oral mucositis from chemoradiotherapy for locally advanced head and neck cancer: phase 3 randomized controlled trial (ROMAN)
Source: eClinicalMedicine. 2025 Oct 8;89:103539. doi: 10.1016/j.eclinm.2025.103539 (PMC12538942; doi:10.1016/j.eclinm.2025.103539)
Supplement: Redacted Protocol [file mmc2.pdf]

CLINICAL STUDY PROTOCOL

Study Title:	ROMAN: Reduction in Oral Mucositis with Avasopasem Manganese (GC4419) – Phase 3 Trial in Patients Receiving Chemoradiotherapy for Locally-Advanced, Non-Metastatic Head and Neck Cancer
Sponsor:	Galera Therapeutics, Inc.<br>[redacted]
IND Number:	111,539
Protocol ID:	GTI-4419-301
Medical Monitor:	[redacted]
Protocol Version/Date:	Amendment 4: 13 July 2020

CONFIDENTIAL INFORMATION

The information contained in this document is proprietary to Galera Therapeutics, Inc. The information is to be used only in connection with review of, or participation in, authorized clinical studies of the investigational drug described in the protocol. Disclosure of the information to others not under obligations to maintain this information in confidence is prohibited without written authorization from Galera Therapeutics, Inc. with the exception that, to the extent necessary to obtain informed consent from those persons to whom the drug may be administered, a general protocol description may be disclosed to such potential subjects.

STUDY ACKNOWLEDGEMENT

Study Title: ROMAN: Reduction in Oral Mucositis with Avasopasem Manganese (GC4419) – Phase 3 Trial in Patients Receiving Chemoradiotherapy for Locally-Advanced, Non-Metastatic Head and Neck Cancer

Protocol No. GTI-4419-301 Final Protocol Date: 13 July 2020

This protocol has been approved by Galera Therapeutics, Inc. The following signature documents this approval.

[redacted]

Chief Medical Officer
Galera Therapeutics, Inc.

Investigator Statement:

I have read the attached protocol dated 13 July 2020 and agree to abide by all provisions set forth therein. I will provide copies of the protocol and other pertinent information to all individuals responsible to me who will assist with the study.

I agree to comply with the International Council for Harmonisation (ICH), Tripartite Guideline on Good Clinical Practice (GCP), applicable Food and Drug Administration (FDA) regulations/guidelines set forth in 21 Code of Federal Regulations (CFR) Parts 11, 50, 54, 56, and 312 and other applicable regulatory authority requirements.

I agree to ensure that Financial Disclosure Statements will be completed before study initiation, during the studies if there are changes that affect my financial disclosure status, and one year after study completion by:

myself (including, if applicable, my spouse [or legal partner] and dependent children)

my sub-investigators (including, if applicable, their spouses [or legal partners] and dependent children)

I agree to ensure that the confidential information contained in this document will not be used for any purpose other than the evaluation or conduct of the clinical investigation without the prior written consent of Galera Therapeutics, Inc.

The Sponsor or its designee will have access to source documentation from which case report forms have been completed.

______________________________ ________________________

Signature of Principal Investigator Date (DD MMM YYYY)

____

Printed Name of Principal Investigator

1. SYNOPSIS

Name of Sponsor/Company: Galera Therapeutics, Inc.	
**Name of Investigational Product: GC4419 (avasopasem manganese)**	
**Name of Active Ingredient:**<br>GC4419 (Manganese, dichloro[(4aS,13aS,17aS,21aS)-1,2,3,4,4a,5,6,12,13,13a,14,15,16,17,17a,18,19,20,21,21a-eicosahydro-11,7- nitrilo-7H-dibenzo[b,h][1,4,7,10] tetraazacycloheptadecine-κN5, κN13, κN18, κN21, κN22]-) is a water soluble, highly stable, low molecular weight manganese-containing macrocyclic ligand complex whose activity mimics that of naturally occurring superoxide dismutase (SOD) enzymes.	
**Title of Study:**<br>ROMAN: Reduction in Oral Mucositis with Avasopasem Manganese (GC4419) – Phase 3 Trial in Patients Receiving Chemoradiotherapy for Locally-Advanced, Non-Metastatic Head and Neck Cancer	
**Number of Study Center(s):**<br>Approximately 100 sites in the United States and Canada	
**Estimated Enrollment Period:**<br>Approximately 32 months	
**Studied Period (years):**<br>Date first subject enrolled: 3-Oct-2018<br>Estimated date last subject enrolled: 2nd quarter 2021<br>Estimated date last subject completed Post-IMRT Phase (completion of follow up for primary efficacy endpoint): 2nd quarter 2021<br>Estimated date for primary efficacy and safety results: 2nd half 2021<br>Estimated date last subject completed Long-term Follow-up: 2nd quarter 2023<br>Estimated date for tumor outcome results: 2nd half 2023	**Phase of development:** 3
**Study Period Definitions:**<br>**Study Treatment Period:** the period of time from Study Day 1 (IMRT Day 1/Baseline) through the last day of IMRT. Subjects who receive at least 60 Gy of IMRT will be considered to have completed the Study Treatment Period.<br>**OM (Oral Mucositis) Observation Period:** the period of time from Study Day 1 (IMRT Day 1/Baseline) through the last OM assessment. By protocol, the last OM assessment is to occur during the second week after completion of IMRT.<br>**AE (Adverse Event) Observation Period:** the period of time from the date of randomization through 30 days post last day of cisplatin, IMRT, or GC4419/Placebo (i.e., whichever occurs last).	

LTFU (Long-term Follow-up) Observation Period: the period of time from last day of IMRT through 24 months post-IMRT.

Objectives:

Primary Objective:

- To assess the effect of GC4419 administered intravenously (IV) on the incidence of severe oral mucositis (SOM; Grade 3-4 OM) during the Study Treatment Period in subjects receiving post-operative or definitive therapy with single-agent cisplatin plus Intensity-Modulated Radiation Therapy (IMRT) for locally advanced, non-metastatic squamous cell carcinoma (SCC) of the oral cavity or oropharynx

Secondary Objectives:

- To assess the effect of GC4419 administered intravenously (IV) on the incidence of Grade 4 OM during the Study Treatment Period in subjects receiving post-operative or definitive therapy with single-agent cisplatin plus Intensity-Modulated Radiation Therapy (IMRT) for locally advanced, non-metastatic squamous cell carcinoma (SCC) of the oral cavity or oropharynx
- To assess the effect of GC4419 administered intravenously (IV) on the total number of days of Grade 3-4 OM during the OM Observation Period in subjects receiving post-operative or definitive therapy with single-agent cisplatin plus Intensity-Modulated Radiation Therapy (IMRT) for locally advanced, non-metastatic squamous cell carcinoma (SCC) of the oral cavity or oropharynx
- To assess the effect of GC4419 administered intravenously (IV) on the total number of days of Grade 4 OM during the OM Observation Period in subjects receiving post-operative or definitive therapy with single-agent cisplatin plus Intensity-Modulated Radiation Therapy (IMRT) for locally advanced, non-metastatic squamous cell carcinoma (SCC) of the oral cavity or oropharynx
- To confirm safety and tolerability of GC4419 administered IV in subjects receiving cisplatin plus IMRT for locally advanced, non-metastatic SCC of the oral cavity or oropharynx
- To evaluate potential effects of GC4419 on tumor efficacy outcomes for the study population

Endpoints:

Primary Endpoint:

- Cumulative incidence of SOM, defined as the proportion of subjects with any occurrence of WHO Grade 3-4 OM, during the Study Treatment Period

Secondary Endpoints:

Efficacy:

- Cumulative incidence of WHO Grade 4 OM, defined as the proportion of subjects with any occurrence of WHO Grade 4 OM, during the Study Treatment Period
- Total number of days (per subject) of SOM (WHO Grade 3-4) during the OM Observation Period for all subjects
- Total number of days (per subject) of Grade 4 OM during the OM Observation Period for all subjects

Safety:

Safety of GC4419 as measured by:

- Frequency, duration, and severity of adverse events (AEs) and serious AEs (SAEs) during the AE Observation Period;
- Incidence and shifts of clinically significant laboratory abnormalities

Effect of treatment on tumor outcomes (locoregional failure, distant metastases, progression-free survival, overall survival) during the LTFU Observation Period

Methodology:

GTI-4419-301 is a randomized, double-blind, placebo-controlled, multi-center international study conducted to evaluate GC4419 administered IV to reduce the incidence and severity of radiation-induced SOM in subjects receiving chemoradiation for squamous cell carcinoma of the head and neck (SCCHN), limited to the oral cavity or oropharynx. Subjects will be randomized in a 3:2 ratio to 1 of 2 treatment arms:

Arm A: 90 mg GC4419 per day (60 min IV infusion to complete within 60 minutes prior to IMRT), concurrent with daily fractions of IMRT (2.0-2.2 Gy) to a total of 60-72 Gy over approximately 7 weeks, plus cisplatin administered 100 mg/m^2 once every three weeks for 3 doses or 40 mg/m^2 once weekly for 6-7 doses (Investigator's choice)

Arm B: Placebo daily (60 min IV infusion to complete within 60 minutes prior to IMRT), concurrent with daily fractions of IMRT (2.0-2.2 Gy) to a total of 60-72 Gy over approximately 7 weeks, plus cisplatin administered 100 mg/m^2 once every three weeks for 3 doses or 40 mg/m^2 once weekly for 6-7 doses (Investigator's choice)

Enrollment will be stratified at randomization for 2 factors: cisplatin schedule (weekly vs q3week); post-operative vs definitive treatment.

Note: Planned radiation fields in both arms must include at least 2 oral sites (left and right buccal mucosa, floor of mouth, left and right ventral/lateral tongue, soft palate) with each site receiving a cumulative dose of at least 50 Gy.

If IMRT is not administered on any given day due to a treatment break or unforeseen circumstances, GC4419/Placebo should not be administered on that day.

All subjects will be assessed twice weekly for OM per WHO grading criteria until the end of the study treatment period (last day of IMRT), and once weekly thereafter for 2 weeks.

The study will be monitored by a Data Monitoring Committee (DMC).

PK sampling will be sought from all subjects.

Number of Subjects (planned):

Approximately 450 total subjects, 270 in the GC4419 arm and 180 in the Placebo arm, will be enrolled from investigational sites in the US and Canada.

Diagnosis and Main Criteria for Inclusion:

Inclusion Criteria:

1. Pathologically-confirmed diagnosis of squamous cell carcinoma of the head and neck, defined as SCC of the oral cavity or oropharynx that will be treated with cisplatin plus concurrent IMRT.

Note: Patients with unknown primary tumors whose treatment plan matches the requirements specified in Inclusion Criteria #2 and #3 below are eligible for the study.

2. Treatment plan to receive a continuous course of IMRT delivered as single daily fractions of 2.0 to 2.2 Gy with a cumulative radiation dose of 60-72 Gy. Planned radiation treatment fields must include at least two oral sites (left and right buccal mucosa, floor of mouth, left and right ventral/lateral tongue, soft palate) that are each planned to receive a total of ≥ 50 Gy. Patients who have had prior surgery are eligible, provided they have fully recovered from surgery, and patients who may have surgery in the future are eligible.

Note: Unavoidable doses of at least 50 Gy, to include entrance, exit, and scatter doses, still constitute planned radiation.

3. Treatment plan to receive standard cisplatin monotherapy administered either every three weeks (100 mg/m^2 for 3 doses) or weekly (40 mg/m^2 for 6-7 doses). The decision on which cisplatin regimen to use in combination with IMRT and GC4419 will be at the discretion of the Investigator.
4. Age 18 years or older
5. Eastern Cooperative Oncology Group (ECOG) performance status ≤ 2
6. Adequate hematologic function as indicated by:
   - Absolute neutrophil counts (ANC) ≥ 1,500/mm^3
   - Hemoglobin (Hgb) ≥ 9.0 g/dL
   - Platelet count ≥ 100,000/mm^3
7. Adequate renal and liver function as indicated by:
   - Serum creatinine acceptable for treatment with cisplatin per institutional guidelines
   - Total bilirubin ≤ 1.5 × upper-normal limit (ULN)
   - Aspartate aminotransferase (AST) and alanine aminotransferase (ALT) ≤ 2.5 × ULN
   - Alkaline phosphatase ≤ 2.5 × ULN
8. Serum pregnancy test negative for females of childbearing potential
9. Males and females must agree to use effective contraception starting prior to the first day of treatment and continuing after the last dose of GC4419/Placebo for 30 days (females) or 90 days (males)
10. Properly obtained written informed consent

Exclusion Criteria:

1. Tumor of the lips, larynx, hypopharynx, nasopharynx, sinuses, or salivary glands
2. M1 disease (distant metastasis)
3. Prior radiotherapy to the region of the study cancer or adjacent anatomical sites or more than 25% of total body marrow-bearing area (potentially interfering with chemo-tolerance)
4. Prior induction chemotherapy for current HNC or any prior immunotherapy
5. Receiving any approved or investigational anti-cancer agent other than those provided for in this study
6. Concurrent participation in another interventional clinical study or use of another investigational agent within 30 days of first dose of GC4419/Placebo

Note: Patients who are participating in non-interventional clinical studies (e.g., QOL, imaging, observational, follow-up studies, etc.) are eligible, regardless of the timing of participation.

7. Inability to eat soft solid food at baseline for reasons other than mouth soreness after surgery or dental procedures
8. Complete reliance on parenteral or gastrointestinal tube-delivered nutrition at baseline

Note: Patients who have gastrostomy tubes prophylactically placed are eligible. Patients receiving supplemental nutrition through a gastrostomy tube at baseline may be eligible depending on diet.

9. Malignant tumors other than head and neck cancer (HNC) within the last 5 years, unless treated definitively and with low risk of recurrence in the judgment of the treating Investigator
10. Active infectious disease excluding oral candidiasis
11. Presence of oral mucositis at baseline. Subjects with mouth or throat pain solely due to post-operative effects are eligible, however.
12. Known history of human immunodeficiency virus (HIV) or history of active hepatitis B/C (patients who have been vaccinated for hepatitis B and do not have a history of infection are eligible)
13. Female patients who are pregnant or breastfeeding
14. Known allergies or intolerance to cisplatin and similar platinum-containing compounds
15. Requirement for concurrent treatment with nitrates or other drugs that may, in the judgment of the treating Investigator, create a risk for a precipitous decrease in blood pressure.
16. Medical history that includes any condition, or requires the use of concomitant medications which, in the Investigator's judgment, are associated with or create a risk of increased carotid sinus sensitivity, symptomatic bradycardia, or syncopal episodes.

Investigational Product, Dosage and Mode of Administration:

GC4419 is formulated as a clear solution at a concentration of 9 mg/mL (Arm A) in 26 mM sodium bicarbonate-buffered 0.9 wt. % saline for parenteral administration (drug product). The GC4419 solution may appear clear or have a slight yellowish tint. GC4419 will be presented in 35 single use

amber vials, one vial per day. Vials will be filled with 11 mL of GC4419, of which 10 mL will be added into a 250 mL bag of normal saline, for daily IV administration over 60 minutes.

Although solutions should be free of particulates, it is possible that some vials may have some fine visible particulates. Infusions must be prepared using a sterile 0.2 or 0.22 micron syringe filter prior to introduction into the infusion bag or a sterile 0.2 or 0.22 micron inline filter must be used during IV administration.

GC4419 will be administered concurrent with daily fractions of IMRT (2.0-2.2 Gy) to a total of 60-72 Gy over 7 weeks, plus cisplatin administered 100 mg/m^2 once every three weeks for 3 doses or 40 mg/m^2 once weekly for 6-7 doses (Investigator's choice).

Duration of Treatment:

Approximately 35 doses, on days in which IMRT is administered, Monday-Friday (M-F), for approximately 7 weeks. GC4419 will be administered within 1 hour prior to each IMRT treatment.

Reference Therapy, Dosage and Mode of Administration:

Placebo will consist of 26 mM sodium bicarbonate-buffered 0.9 wt. % saline for parenteral administration only (Arm B). Placebo will be presented in 35 single use amber vials, one vial per day. Vials will be filled with 11 mL of Placebo, of which 10 mL be added into a 250 mL bag of normal saline, for daily IV administration over 60 minutes. Placebo will be administered concurrent with daily fractions of IMRT (2.0-2.2 Gy) to a total of 60-72 Gy over 7 weeks, plus cisplatin administered 100 mg/m^2 once every three weeks for 3 doses or 40 mg/m^2 once weekly for 6-7 doses (Investigator's choice).

Criteria for Evaluation:

Efficacy:

WHO Criteria for OM

Safety:

National Cancer Institute (NCI) - Common Terminology Criteria for Adverse Events (CTCAE), version 5.0

Clinical Laboratory Assessment: A central laboratory vendor will be utilized.

DMC Review

Toxicity Management:

Toxicity requiring 25% GC4419 or Placebo dose reduction:

Grade 2 or greater hypotension within 2 hours after the start of GC4419/Placebo infusion (i.e., anytime from start of infusion through 1 hour after end of infusion).

The dose of GC4419 or Placebo may be reduced by 25% for Grade 3-4 adverse events (AEs) judged by the Investigator to be likely attributable to the study infusion.

Two dose reductions for toxicity will be permitted per subject. Subjects unable to tolerate GC4419/Placebo after 2 dose reductions must discontinue treatment with GC4419/Placebo but should continue with cisplatin/IMRT and other study assessments and procedures, with the concurrence of the treating Investigator, if the subject maintains informed consent to do so.

For other toxicities (including those attributable to cisplatin and IMRT): management per institutional and American Society of Clinical Oncology (ASCO) guidelines and Investigator judgment. Cisplatin

toxicities should be managed by modification of the cisplatin dose and schedule, not by substitution of another systemic agent.<br>Multinational Association of Supportive Care in Cancer (MASCC)/International Society of Oral Oncology (ISOO) Oral Care Education Materials and patient instructions for oral hygiene will be strongly recommended, and a summary appended to the protocol (Appendix 6).<br>OM will NOT be considered an AE requiring dose modification for the purposes of this study.
**Concomitant Medications/Treatments:**<br>Investigators may prescribe any concomitant medication or supportive therapy deemed necessary to provide adequate supportive care including antiemetics, systemic antibiotics, hydration to prevent renal damage, topical fluoride etc., with the following exceptions:<br>Low-level laser treatment for OM<br>Amifostine (Ethyol®)<br>Benzydamine (Difflam®, Pharixia®, Tantum Verde)<br>Cetuximab (Erbitux®)<br>Glutamine in any form<br>GM-CSF applied topically<br>'Magic mouthwashes' or 'Miracle mouthwashes' are permitted, provided they do not contain:<br>– Chlorhexidine<br>– Hydrogen peroxide<br>– Diphenhydramine (Benadryl®) liquid formulation<br>– Tetracycline<br>– Any other listed disallowed medications<br>– Notes:<br>o Topical lidocaine preparations are permitted<br>o Diphenhydramine (Benadryl®) administered as tablets or by injection is permitted<br>MuGard™, Gelclair®, Episil®, or other barrier devices<br>Caphosol®<br>Nitrates, phosphodiesterase type 5 (PDE 5) inhibitors (e.g., sildenafil, tadalafil, or similar agents) or other drugs that in the judgment of the treating Investigator could create a risk of a precipitous decrease in blood pressure are prohibited until at least 24 hours after the last dose of GC4419<br>Pyridostigmine or other drugs that in the judgment of the treating Investigator could create a risk of increased carotid sinus sensitivity, symptomatic bradycardia, or syncopal episodes<br>Palifermin (Kepivance®) or other keratinocyte or fibroblast growth factor<br>Povidone-iodine rinses

Steroid rinses

Sucralfate in suspension form (use of sucralfate tablets is permitted)

Other biologic response modifiers – except systemic hematopoietic growth factors for the management of anemia or myelosuppression

Concurrent approved or investigational anti-cancer therapy (e.g., chemotherapy, immunotherapy, targeted therapy, hormone and biologic therapy) other than the Protocol regimen

Other investigational agents or perceived herbal/homeopathic or other remedies for OM, which are considered investigational for purposes of this study (includes nitric oxide)

All medication restrictions begin on Day 1 of IMRT and GC4419/Placebo. All medication restrictions end after post-IMRT OM follow-up is completed unless otherwise noted.

Anti-emetic prophylaxis and hematopoietic growth factor use are permitted per ASCO guidelines. Following ASCO (and MASCC) guidelines for the prevention and management of chemotherapy-induced nausea and vomiting (CINV) is strongly encouraged.

Oral care per MASCC/ISOO Oral Care Education Materials (summarized in Appendix 6) will be strongly recommended for all subjects as part of standard of care.

Statistical Methods:

Approximately 450 subjects will be enrolled (270 GC4419:180 Placebo) to obtain approximately 400 Intent-to-Treat (ITT) subjects (240 GC4419:160 Placebo). The proposed ITT sample size of 400 assumes that 10% will discontinue early, yielding approximately 216 subjects in the experimental arm and approximately 144 in the Placebo arm who will complete their IMRT course. The study will have approximately 95% power to detect a relative reduction in incidence of severe OM throughout the study treatment period in the experimental arm of approximately 34% compared to Placebo (43% incidence for GC4419 vs 65% for Placebo).

Safety and efficacy will be assessed on the ITT population consisting of all randomized subjects who receive at least one dose of GC4419/Placebo, excluding the 28 subjects whose dosing was interrupted due to the Sponsor's dosing suspension decision.

Analysis of incidence/proportion endpoints will use the Cochran-Mantel-Haenszel (CMH) test stratified by the randomization factors as entered at treatment assignment. The study hypothesis for the primary endpoint will be tested at a 2-sided Type 1 error rate of 0.05.

To control the overall Type 1 error at 0.05, the primary endpoint will first be tested at a two-sided 0.05 level. If the primary endpoint result is significant at this level, then Holm-Bonferroni multiplicity testing will be conducted on the following proposed secondary endpoints applying a familywise two-sided alpha of 0.05:

- incidence of Grade 4 OM during the Study Treatment Period;
- total number of SOM days (per subject) during the OM Observation Period for the entire study population; and
- total number of days of Grade 4 OM (per subject) during the OM Observation Period for the entire study population.

Efficacy analyses will be stratified by the randomization strata:

Cisplatin schedule (weekly vs q3weekly)

Post-operative vs definitive treatment.

2. TABLE OF CONTENTS

TABLE OF CONTENTS

1. SYNOPSIS 3
2. TABLE OF CONTENTS 11
3. GLOSSARY 16
4. INTRODUCTION 19
4.1. Background 19
4.1.1. Oral Mucositis in Patients Treated with Chemoradiation for HNC 19
4.1.2. Oral Mucositis is an Unmet Medical Need 20
4.2. GC4419 Overview 21
4.3. Study Period Definitions 22
5. STUDY OBJECTIVES, ENDPOINTS AND PURPOSE 23
5.1. Objectives 23
5.2. Endpoints 24
5.2.1. Rationale for Primary Endpoint 25
6. INVESTIGATIONAL PLAN 26
6.1. Overall Study Design 26
6.2. Treatment Plan and Duration of Therapy 27
6.2.1. Rationale for GC4419 Dose and Schedule Selection 28
6.2.2. Rationale for Cisplatin/IMRT Treatment Plan 28
6.2.3. Rationale for Tumor Follow-up and Analysis 29
7. SELECTION AND WITHDRAWAL OF SUBJECTS 29
7.1. Subject Inclusion Criteria 29
7.2. Subject Exclusion Criteria 30
7.3. Rationale for Patient Population 31
7.4. Screen Failures 32
7.5. Randomization Failures 32
7.6. Subject Withdrawal Criteria 32
7.7. Study and Site Closure 33
8. GC4419/PLACEBO 33
8.1. Description of GC4419/Placebo 33
8.1.1. GC4419 33

8.1.2. Placebo 34
8.2. Treatment Assignment 34
8.2.1. Blinding 34
8.3. GC4419/Placebo Packaging and Labeling 34
8.3.1. GC4419 35
8.3.2. Placebo 35
8.4. GC4419/Placebo Storage 35
8.5. GC4419/Placebo Preparation 35
8.6. GC4419/Placebo Administration 36
8.7. GC4419/Placebo Accountability and Compliance 39
8.8. GC4419/Placebo Handling and Disposal 39
8.9. Concomitant Medications 39
8.9.1. Prohibited Medications 40
8.9.2. CYP2D6 Substrates 41
9. TOXICITY MANAGEMENT 42
9.1. Dose Delays and Dose Modifications for Toxicity 42
9.2. Supportive Care Guidelines 42
9.2.1. Supportive care for chemotherapy-induced nausea and vomiting (CINV) 43
10. ASSESSMENTS 43
10.1. Safety Assessments 43
10.1.1. Clinical Assessments 43
10.1.2. Laboratory Assessments 44
10.2. Oral Mucositis Assessments 44
10.3. Radiation Therapy Quality Assurance 45
10.4. Pharmacokinetic (PK) Measurements 45
10.4.1. Pharmacokinetic (PK) Measurements of GC4419 45
10.5. Biological Surrogate Markers of Mucositis 46
10.6. Tumor Status Assessment 46
10.6.1. Clinical Tumor Assessment 46
10.6.2. Tumor Imaging 47
10.6.2.1. Pre-Treatment Tumor Imaging 47
10.6.2.2. Post-Treatment Tumor Imaging 47
10.6.3. Survival Status 47

10.7. Schedule of Time and Events 47
10.8. Screening 48
10.9. Treatment 49
10.9.1. Baseline/Day 1 (First Day of IMRT and GC4419/Placebo) 49
10.9.2. Day 2 of IMRT and GC4419/Placebo 49
10.9.3. Days 3 to 5 of IMRT and GC4419/Placebo 50
10.9.4. Week 2 50
10.9.5. Week 3 51
10.9.6. Week 4 51
10.9.7. Week 5 52
10.9.8. Week 6 53
10.9.9. Week 7 (plus additional IMRT weeks, if needed) 53
10.9.10. Last Day of IMRT or Early Termination Visit 54
10.10. Follow-up 54
10.10.1. Post-IMRT OM Follow-up 54
10.10.2. Long-term Follow-up 55
11. ADVERSE EVENTS AND SERIOUS ADVERSE EVENTS 55
11.1. Definitions 56
11.1.1. Adverse Event (AE) 56
11.1.2. Serious Adverse Event (SAE) 56
11.2. Adverse Event Reporting Requirements 57
11.2.1. Serious Adverse Events 57
11.2.2. All Adverse Events (AEs) Regardless of Seriousness 57
11.2.3. Clinical Laboratory Abnormalities and Other Abnormal Assessments as AEs and SAEs 57
11.2.4. Disease-Related Events and/or Disease-Related Outcomes Not Qualifying as AEs or SAEs 58
11.2.5. Grading of Adverse Events 58
11.3. Relationship to Study Drug 58
11.4. Recording Adverse Events 59
11.5. Follow-up of AEs and SAEs 60
11.6. Post-Study Reporting Requirements 60
11.7. Regulatory Reporting of Adverse Events 61

11.8. Pregnancy 61
11.8.1. Time Period for Collecting Pregnancy Information 61
11.8.2. Action to be Taken if Pregnancy Occurs in a Female Partner of a Male Subject 61
12. STATISTICS 62
12.1. General Considerations....62
12.1.1. Randomization and Stratification 62
12.1.2. Treatment of Subjects Affected by Sponsor Dosing Suspension 62
12.2. Sample Size 62
12.3. Analysis Populations 63
12.4. Safety Analysis 63
12.5. Primary Efficacy Analysis 63
12.5.1. Multiplicity 63
12.5.2. Handling of Missing Data....64
12.5.3. Covariate Adjustment 64
12.6. Secondary Efficacy Analyses 64
12.6.1. Secondary Endpoints 64
12.7. Exploratory Efficacy Analyses 64
12.8. Analysis of Tumor Endpoints....65
12.8.1. Local or Regional Progression....65
12.8.2. Distant Metastasis....65
12.8.3. Second Primary Neoplasm 65
12.9. Interim Analysis....65
13. ETHICS 65
13.1. Ethical Conduct of the Study....65
13.2. Institutional Review Board (IRB)/Independent Ethics Committee (IEC)/Research Ethics Board (REB) Approval 65
13.3. Written Informed Consent 66
13.4. Data Monitoring Committee (DMC) 66
14. DIRECT ACCESS TO SOURCE DATA/DOCUMENTS....66
14.1. Study Monitoring....66
14.2. Audits and Inspections....67
14.3. Protocol Compliance 67

14.4. Protocol Modifications 67
14.5. Information Disclosure 67
14.5.1. Ownership 67
14.5.2. Confidentiality 68
14.5.3. Publication 68
15. QUALITY CONTROL AND QUALITY ASSURANCE 68
16. DATA HANDLING AND RECORDKEEPING 68
16.1. Case Report Forms 68
16.2. Retention/Inspection of Records 68
17. LIST OF REFERENCES 70
18. APPENDICES 74
APPENDIX 1. SCHEDULE OF ASSESSMENTS 75
APPENDIX 2. WORLD HEALTH ORGANIZATION (WHO) SCORE OF ORAL MUCOSITIS 78
APPENDIX 3. PERFORMANCE STATUS CONVERSION 79
APPENDIX 4. NATIONAL CANCER INSTITUTE-COMMON TERMINOLOGY CRITERIA FOR ADVERSE EVENTS, VERSION 5.0 80
APPENDIX 5. RECOMMENDED REGIMENS FOR HIGH-RISK CHEMOTHERAPY-INDUCED NAUSEA AND VOMITING (CINV) PER ASCO AND MASCC GUIDELINES 81
APPENDIX 6. DAILY ORAL CARE RECOMMENDATIONS 83
APPENDIX 7. CYP2D6 SUBSTRATES 84
APPENDIX 8. SUMMARY OF CHANGES IN AMENDMENT 4 85

LIST OF TABLES

Table 1: Abbreviations and Specialist Terms 16
Table 2: Comparison of WHO Scale vs. NCI-CTCAE v5.0 for Oral Mucositis 26
Table 3: Chemoradiation and GC4419/Placebo Administration Schedule Example: 35 Doses of GC4419/Placebo (5 days/week over 7 Week Schedule) 37
Table 4: Pharmacokinetic Sampling Schedule to Assess GC4419 46
Table 5: Adverse Event Severity 58
Table 6: Schedule of Assessments 75
Table 7: Antiemetic Dosing for Adults – High Risk (Cisplatin) 81

3. GLOSSARY

The following abbreviations and specialist terms are used in this study protocol.

Table 1: Abbreviations and Specialist Terms

Abbreviation or Specialist Term	Definition
AE	Adverse Event
AJCC	American Joint Committee on Cancer
ALT	Alanine Aminotransferase
ANC	Absolute Neutrophil Count
ASCO	American Society of Clinical Oncology
AST	Aspartate Aminotransferase
BSA	Body Surface Area
BUN	Blood Urea Nitrogen
CFR	Code of Federal Regulations
CINV	Chemotherapy-Induced Nausea and Vomiting
CMH	Cochran-Mantel-Haenszel
CRF	Case Report Form
CRO	Contract Research Organization
CRT	Chemo-Radiotherapy
CS	Clinically Significant
CTCAE	Common Terminology Criteria for Adverse Events
CYP2D6	Cytochrome P450 Isozyme 2D6
DDDP	Division of Dermatology and Dental Products
DMC	Data Monitoring Committee
DP	Drug Product
ECG	Electrocardiogram
ECOG	Eastern Cooperative Oncology Group
FDA	Food and Drug Administration
GCP	Good Clinical Practice
Gy	Gray

Abbreviation or Specialist Term	Definition
Hgb	Hemoglobin
HIV	Human Immunodeficiency Virus
HNC	Head and Neck Cancer
HPV	Human Papilloma Virus
HSCT	Hematopoietic Stem Cell Transplantation
ICF	Informed Consent Form
ICH	International Council for Harmonisation
IEC	Independent Ethics Committee
IMRT	Intensity-Modulated Radiation Therapy
IND	Investigational New Drug
IRB	Institutional Review Board
IRT	Interactive Response Technology
ISOO	International Society of Oral Oncology
ITT	Intent-to-Treat
IV	Intravenous
IXRS	Interactive Voice and Web Response System
LTFU	Long-term Follow-up
MASCC	Multinational Association of Supportive Care in Cancer
M-F	Monday to Friday
MnSOD	Manganese Superoxide Dismutase
NCI	National Cancer Institute
NCS	Non-Clinically Significant
NEPA	Netupitant and Palonosetron
OC	Oral Cavity
OM	Oral Mucositis
OP	Oropharynx
OS	Overall Survival
PFS	Progression Free Survival

Abbreviation or Specialist Term	Definition
PK	Pharmacokinetic
QOL	Quality of Life
REB	Research Ethics Board
RNA	Ribonucleic Acid
RT	Radiation Therapy
SAE	Serious Adverse Event
SAS	Statistical Analysis System
SCC	Squamous Cell Carcinoma
SCCHN	Squamous Cell Carcinoma of the Head and Neck
SGOT	Serum Glutamic-Oxaloacetic Transaminase
SGPT	Serum Glutamic-Pyruvic Transaminase
SOC	Standard of Care
SOD	Superoxide Dismutase
SOM	Severe Oral Mucositis
ULN	Upper Limit of Normal
WHO	World Health Organization

4. INTRODUCTION

4.1. Background

4.1.1. Oral Mucositis in Patients Treated with Chemoradiation for HNC

Oral mucositis (OM) is a common, problematic, and painful complication of cancer therapy, particularly in regimens that include radiation to the head and neck (Sonis 2009). Oral mucositis is readily graded using the commonly-used five-point World Health Organization (WHO) scale (Appendix 2).

Most patients receiving combined chemoradiotherapy for head and neck cancer (HNC) can be expected to develop severe (WHO Grade 3-4) OM (Sonis, Elting et al. 2004), and nearly all HNC patients receiving radiation therapy (RT) with concurrent cisplatin are expected to develop ulcerative OM (WHO Grade 2 or higher) (Traynor, Richards et al. 2010). The consequences for patients developing severe OM are especially serious, because by definition, Grade 3-4 OM entails compromise to nutrition and/or hydration, and may require surgical procedure, RT interruption, and/or hospitalization.

Standard chemoradiotherapy for locally advanced squamous cell carcinoma of the head and neck (SCCHN), whether in the post-operative or definitive setting, currently consists of intensity-modulated radiation therapy (IMRT) plus systemic therapy. Common systemic treatment is with single-agent cisplatin, administered either q3 weeks or once weekly schedule (Mendenhall, Amdur et al. 2006; Salama, Seiwert et al. 2007; Ang, Zhang et al. 2014; Nguyen-Tan, Zhang et al. 2014). The monoclonal antibody cetuximab has also been shown to increase the efficacy of radiotherapy for locally advanced head and neck cancer (Bonner, Harari et al. 2006). Recently reported results from the RTOG 1016 study in human papilloma virus (HPV)-associated oropharyngeal cancer showed survival after IMRT plus cetuximab to be inferior to that obtained with IMRT/cisplatin (Gillison, et al. 2018).

Published observations for patients treated with RT for HNC, without additional treatment to prevent OM, indicate that approximately 50% of patients receiving RT alone (Bonner, Harari et al, 2006), and that approximately 70% of patients receiving RT plus single-agent cisplatin may be expected to develop severe OM, with a median duration of approximately 3-4 weeks among those who develop severe OM, a median time to onset of approximately 28-35 days after the start of therapy, and a cumulative incidence of approximately 10% per week/10 Gy. Data for the placebo groups in two published Phase 3 studies of Kepivance® (palifermin) in patients receiving RT plus single-agent cisplatin for SCCHN (Henke, Alfonsi et al. 2011; Le, Kim et al. 2011), and results for patients receiving IMRT plus platinum plus placebo in a more recent report (Kudrimoti, Curtis et al. 2016) support these observations. Other observations also supporting these expectations come from aggregate data from approximately 380 patients in placebo groups who received IMRT/cisplatin for oral cavity or oropharyngeal cancers (S. Sonis, Dana Farber Cancer Institute, personal communication).

Adding cetuximab to standard chemoradiation regimens for HNC increases the risk of significant OM (Ang, Zhang et al. 2014), while cetuximab without additional chemotherapy has been reported in one study to increase the risk of OM in patients receiving radiation modestly, if at all (Bonner, Harari et al. 2006). In RTOG 1016, the incidence of NCI Grade 3-4 OM was similar for RT plus cetuximab and RT plus cisplatin (Gillison, et al. 2018).

Among patients being treated for HNC, OM follows a predictable and well-documented course (Sonis 2011). By the end of the first week of treatment (typically cumulative radiation doses of 10 Gy), erythema of the oral mucosa is usually seen and patients complain of discomfort that is characterized as burning. This relatively mild pain escalates between the second and third week of treatment (radiation doses of 20 Gy to 30 Gy), when frank ulceration of the mucosa develops. Lesions at this stage often necessitate a modification in food intake and a marked increase in the need for analgesics. Individual ulcers frequently coalesce as radiation progresses resulting in confluent injury affecting many aspects of the oral mucosa. Pain intensifies and may be inadequately controlled even with aggressive narcotic therapy (Elting, Cooksley et al. 2007).

OM has substantial impact on day-to-day functioning. In addition to the common need for, and inadequate pain control with, narcotics, the profound clinical impact of OM also includes weight loss, difficulty eating and swallowing, dehydration, need for nutritional support, and reduced performance status (Elting, Cooksley et al. 2007), as well as secondary infections at sites of ulcerative OM (Bodey, Rodriguez et al. 1978), and diminished quality of life (QOL) outcomes (Elting, Keefe et al. 2008). Patients with HNC may also suffer the additional complications of short- and long-term xerostomia, taste change, and trismus related to post-radiation fibrosis. These consequences are particularly pronounced in patients manifesting severe OM, and especially in the 20-30% who suffer Grade 4 and require artificial alimentation, usually via a percutaneous gastrostomy tube.

Among patients treated for HNC, even mild mucositis results in more frequent hospitalization and breaks in treatment, introducing the risk of compromised anti-tumor efficacy (Vera-Llonch, Oster et al. 2006; Russo, Haddad et al. 2008). In granulocytopenic patients, mucositis is strongly associated with an increased risk of bacteremia and sepsis (Ruescher, Sodeifi et al. 1998). Adverse health economic outcomes include increased analgesic and antibiotic use, increased number of febrile days, need for parenteral nutrition, prolonged length of hospital stay, and increased resource use and associated cost (Nonzee, Dandade et al. 2008).

4.1.2. Oral Mucositis is an Unmet Medical Need

Oral mucositis prevention and management remains a substantial unmet need. For years, there has been no substantial change in its management (Keefe 2006). Current guidelines (Lalla, Bowen et al. 2014) from the Multinational Association of Supportive Care in Cancer and the International Society of Oral Oncology (MASCC/ISOO) limit recommended or suggested interventions to prevent OM to:

- Kepivance® (palifermin) in the setting of high-dose chemotherapy and total body irradiation, followed by autologous hematopoietic stem cell transplantation (HSCT), for a hematological malignancy. However, Kepivance® is not licensed/approved for the treatment of OM associated with HNC;
- Oral cryotherapy in the setting of 5-fluorouracil therapy or chemotherapy/HSCT;
- Low-level laser therapy in the setting of chemotherapy/HSCT or chemoradiotherapy for HNC;
- Benzydamine mouthwash in the setting of HNC treated with moderate-dose RT, without concomitant chemotherapy;
- Oral zinc supplements in the setting of radiation or chemoradiation;
- Oral care protocols across all cancer treatment modalities.

Narcotics and doxepin mouthwash are recommended or suggested by MASCC/ISOO for the treatment of OM-related pain. Several other agents currently used to treat OM symptoms, while designed to palliate associated pain or manage infection, do not alter the underlying biologic processes that give rise to OM. MASCC/ISOO currently recommends or suggests *against* the use of some agents that have historically been used (e.g., antimicrobial mouthwashes, sucralfate, chlorhexidine mouthwash), citing evidence for lack of effectiveness against OM in one or more treatment settings.

Further, no approved product is available in the US (or elsewhere) for OM in the vast majority of these patients, especially those with solid tumors such as SCCHN. At present, palifermin is the only FDA-approved (licensed) product for OM but its indicated use (patients receiving HSCT for hematologic malignancies) is limited to a very small cohort (4%) of the total population at risk for OM: a cohort associated with conditioning regimens prior to stem cell transplant for the treatment of hematologic malignancies. The approval specifically excludes patients with solid tumors (Spielberger, Stiff et al. 2004).

A few medical devices are on the market, but they generally lack sufficient data to allow recommendation. The mucoadhesive MuGard™ is indicated as a palliative treatment for the management of OM but requires administration 4 to 6 times per day for optimal effect and does not alter the mechanism of OM (MuGard prescribing information). The oral gel Gelclair® has a similar use and effect for OM or oral irritation due to other causes but does not affect the mechanism of OM. Caphosol® (supersaturated calcium phosphate rinse) is indicated for xerostomia or as an adjunct to standard oral care for OM but failed to reduce the incidence of ulcerative or severe OM in a recently-published Phase 2 study (Rao, Trotti et al. 2014). The lipid-based oral barrier rinse Episil® and the bacteriostatic rinse GelX® (zinc gluconate-taurine complex) may be used for pain related to OM but do not affect the mechanism causing OM.

Therefore, current standard of care for patients with solid tumors consists of oral care protocols and palliative approaches to deal with painful symptoms. An urgent need remains for novel products, like GC4419, aimed at reducing the incidence and severity of severe OM.

4.2. GC4419 Overview

GC4419 is a novel, highly stable manganese-containing macrocyclic ligand complex with a molecular weight of 483 g/mol, whose activity mimics that of naturally occurring superoxide dismutase (SOD) enzymes. It is therefore a prototype of a new class of drugs termed selective SOD mimetics. GC4419 selectively removes superoxide anions without reacting with other reactive oxygen species, including nitric oxide, hydrogen peroxide, and peroxynitrite. In addition, unlike native SOD, GC4419 is not deactivated by nitration.

GC4419 is being developed for the initial indication of reduction of the incidence and severity of severe OM induced by RT, with or without systemic therapy, under IND 111,539 with the Division of Dermatology and Dental Products (DDDP), United States FDA.

By efficiently and rapidly removing O2•- , GC4419 offers a treatment paradigm for preventing or controlling OM. Numerous published studies indicate that OM, esophagitis, pneumonitis, fibrosis, or other normal-tissue radiation damage may be reduced by treatment with liposomally encapsulated exogenous manganese (Mn) SOD or a MnSOD transgene, exogenous Cu/Zn SOD, or a dismutase mimetic enantiomerically related to GC4419 (Delanian, Baillet et al. 1994; Epperly, Defilippi et al. 2000; Guo, Seixas-Silva et al. 2003; Murphy, Fey et al. 2008; Thompson, Chu et al. 2010).

GC4419 was studied extensively in nonclinical settings, in different animal models, and various human xenograft and murine syngeneic tumor models (for details see the Investigator's Brochure). Nonclinical data have identified GC4419 as a promising new radioprotective, anti-cancer and anti-inflammatory agent. GC4419 was active as a radio-protectant in animal models of cancer radiation therapy and added to the activity of chemotherapeutic agents in animal models of cancer. Importantly, GC4419 did not interfere with the anti-tumor effects of either radiation therapy or chemotherapy in animal models of cancer.

GC4419 was studied in a Phase 1b/2a clinical study, GT-001 (NCT01921426, Anderson, Sonis, et al. 2018) and Phase 2b study, GT-201 (NCT02508389). In study GT-001, GC4419 was administered throughout the full 6- to 7-week course of chemo-radiotherapy (CRT) at doses of either 30 or 90 mg, Monday-Friday (M-F), and appeared to substantially reduce the duration, incidence, and overall severity (defined here as the incidence specifically of Grade 4) of severe OM compared with historical expectations (data are based on 43 evaluable subjects). Further, GC4419 did not appear to increase the known toxicity of the CRT regimen (IMRT plus concurrent cisplatin), nor did it appear to interfere with tumor response to CRT assessed through 1 year post therapy (for details see Anderson, Sonis, et al. 2018 and the Investigator's Brochure). These results supported the design of the randomized, double-blind, placebo-controlled, Phase 2b study GT-201, with two GC4419 doses of 30 and 90 mg selected for comparison with placebo and administered 5 days/week. As in GT-001, patients eligible for GT-201 were required to have locally advanced squamous cell carcinoma of the oral cavity (OC) or oropharynx (OP), with their standard care calling for concurrent IMRT and cisplatin. Results from 223 randomized subjects indicated that GC4419 at daily dose of 90 mg resulted in statistically significant reduction on the primary endpoint, severe OM duration, for subjects on the 90 mg arm compared to placebo, as determined by the non-parametric Van Elteren test. The median duration of SOM, as defined in the protocol, was 1.5 days for subjects receiving 90 mg of GC4419 vs 19 days for subjects receiving placebo (p=0.024). Also, the incidence of SOM during the IMRT treatment period was 43% for subjects receiving 90 mg of GC4419 vs 65% for subjects receiving placebo (nominal p value=0.009), a relative reduction of approximately 34%. The safety profile of GC4419 in combination with the IMRT/platinum regimen in GT-201 study was acceptable; safety results with both the 90 mg dose and the 30 mg dose were similar to those with placebo.

Additional details about the pathogenesis of OM, the mechanism of action of GC4419, and the results of non-clinical testing as well as the clinical trials GT-001 and GT-201 are provided in the Investigator's Brochure.

4.3. Study Period Definitions

Study Treatment Period: the period of time from Study Day 1 (IMRT Day 1/Baseline) through the last day of IMRT. Subjects who receive at least 60 Gy of IMRT will be considered to have completed the Study Treatment Period.

OM (Oral Mucositis) Observation Period: the period of time from Study Day 1 (IMRT Day 1/Baseline) through the last OM assessment. By protocol, the last OM assessment is to occur during the second week after completion of IMRT.

AE (Adverse Event) Observation Period: the period of time from the date of randomization through 30 days post last day of cisplatin, IMRT, or GC4419/Placebo (i.e., whichever occurs last).

LTFU (Long-term Follow-up) Observation Period: the period of time from last day of IMRT through 24 months post-IMRT.

5. STUDY OBJECTIVES, ENDPOINTS AND PURPOSE

5.1. Objectives

Primary Objective:

To assess the effect of GC4419 administered intravenously (IV) on the incidence of severe oral mucositis (SOM; Grade 3-4 OM) during the Study Treatment Period in subjects receiving post-operative or definitive therapy with single-agent cisplatin plus Intensity-Modulated Radiation Therapy (IMRT) for locally advanced, non-metastatic squamous cell carcinoma (SCC) of the oral cavity or oropharynx

Secondary Objectives:

To assess the effect of GC4419 administered intravenously (IV) on the incidence of Grade 4 OM during the Study Treatment Period in subjects receiving post-operative or definitive therapy with single-agent cisplatin plus Intensity-Modulated Radiation Therapy (IMRT) for locally advanced, non-metastatic squamous cell carcinoma (SCC) of the oral cavity or oropharynx

To assess the effect of GC4419 administered intravenously (IV) on the total number of days of Grade 3-4 OM during the OM Observation Period in subjects receiving post-operative or definitive therapy with single-agent cisplatin plus Intensity-Modulated Radiation Therapy (IMRT) for locally advanced, non-metastatic squamous cell carcinoma (SCC) of the oral cavity or oropharynx

To assess the effect of GC4419 administered intravenously (IV) on the total number of days of Grade 4 OM during the OM Observation Period in subjects receiving post-operative or definitive therapy with single-agent cisplatin plus Intensity-Modulated Radiation Therapy (IMRT) for locally advanced, non-metastatic squamous cell carcinoma (SCC) of the oral cavity or oropharynx

To confirm safety and tolerability of GC4419 administered IV in subjects receiving cisplatin plus IMRT for locally advanced, non-metastatic SCC of the oral cavity or oropharynx

To evaluate potential effects of GC4419 on tumor efficacy outcomes for the study population

Exploratory Objectives:

To assess duration (per subject) of SOM (WHO Grade 3-4) for subjects who have experienced SOM during the OM Observation Period

To assess duration of Grade 4 OM for subjects who have experienced Grade 4 OM during the OM Observation Period

To assess effects of GC4419 on the cumulative incidence of SOM (WHO Grade 3-4) from the first IMRT fraction through the delivery of progressive, cumulative IMRT doses

To assess effects of GC4419 on the cumulative incidence of SOM (WHO Grade 3-4) during the OM Observation Period

To assess time from beginning of IMRT and IMRT dose to onset of SOM (WHO Grade 3-4)

To assess resolution (defined as ≤ Grade 2 OM) of SOM (WHO Grade 3-4) before the end of the Study Treatment Period for subjects who have experienced SOM during the Study Treatment Period

To assess resolution (defined as ≤ Grade 3 OM) of Grade 4 OM before the end of the Study Treatment Period for subjects who have experienced Grade 4 OM during the Study Treatment Period

To assess effects of GC4419 on the incidence, onset, and duration of ulcerative OM (≥ Grade 2)

To assess treatment delivery and delays (number and duration of delays) of IMRT and cisplatin

To assess the relationship between drug exposure and safety/efficacy

To assess the effects of GC4419 on circulating cytokine levels and gene expression levels

To assess the effects of GC4419 on kidney function throughout the first year post-IMRT

5.2. Endpoints

Primary Endpoint:

Cumulative incidence of SOM, defined as the proportion of subjects with any occurrence of WHO Grade 3-4 OM, during the Study Treatment Period

Secondary Endpoints:

Efficacy:

Cumulative incidence of WHO Grade 4 OM, defined as the proportion of subjects with any occurrence of WHO Grade 4 OM, during the Study Treatment Period

Total number of days (per subject) of SOM (WHO Grade 3-4) during the OM Observation Period for all subjects

Total number of days (per subject) of Grade 4 OM during the OM Observation Period for all subjects

Safety:

Safety of GC4419 as measured by:

- Frequency, duration, and severity of Adverse Events (AEs) and serious AEs (SAEs) during the AE Observation Period;
- Incidence and shifts of clinically significant laboratory abnormalities

Effect of treatment on tumor outcomes (locoregional failure, distant metastases, progression-free survival, overall survival) during the LTFU Observation Period

Exploratory Endpoints:

Duration (per subject) of SOM (WHO Grade 3-4) for subjects who have experienced SOM during the OM Observation Period

Duration of Grade 4 OM for subjects who have experienced Grade 4 OM during the OM Observation Period

- Effects of GC4419 on the cumulative incidence of SOM (WHO Grade 3-4) from the first IMRT fraction through the delivery of progressive, cumulative IMRT doses
- Effects of GC4419 on the cumulative incidence of SOM (WHO Grade 3-4) during the OM Observation Period
- Time from beginning of IMRT and IMRT dose to onset of SOM (WHO Grade 3-4)
- Resolution (defined as ≤ Grade 2 OM) of SOM (WHO Grade 3-4) before the end of the Study Treatment Period for subjects who have experienced SOM during the Study Treatment Period
- Resolution (defined as ≤ Grade 3 OM) of Grade 4 OM before the end of the Study Treatment Period for subjects who have experienced Grade 4 OM during the Study Treatment Period
- Effects of GC4419 on the incidence, onset, and duration of ulcerative OM (≥ Grade 2)
- Treatment delivery and delays (number and duration of delays) of IMRT and cisplatin
- Relationship between drug exposure and safety/efficacy
- Effects of treatment assignment on circulating cytokine levels and gene expression levels
- Effects of treatment assignment on kidney function throughout the first year post-IMRT

5.2.1. Rationale for Primary Endpoint

The WHO scale is commonly used to assess OM in clinical care and research settings. Originally developed as a standard toxicity reporting index, it has evolved into the most accepted outcome for efficacy testing of drugs as it has been shown to meet key characteristics for clinical studies:

- Accurate reflection of the severity and course of the objective and subjective changes of mucositis.
- Easy to teach with a low inter-observer variability.
- Does not require measurement of lesions.
- Sensitive enough to discriminate treatment efficacy.
- Clinically meaningful and easily interpreted endpoints for clinicians, patients and regulatory agencies.

The WHO scale has been used as the primary efficacy endpoint for many studies and is accepted internationally.

There is precedent for using severe OM as defined by the WHO scale as the basis for drug approval. Palifermin (Kepivance®) is indicated to decrease the incidence and duration of severe OM in patients with hematologic malignancies receiving myelotoxic therapy requiring hematopoietic stem cell support when the preparative regimen is predicted to result in WHO Grade > 3 OM in the majority of patients. Approval of palifermin was based on a reduction in the number of days during which patients experienced severe OM (Grade 3/4 on the WHO scale); other analyses included the incidence, duration, and severity of oral mucositis and the use of opioid analgesia (Kepivance prescribing information). Additional studies of agents to reduce OM—notably the 2 published studies of palifermin in the HNC population (Henke, Alfonsi et al. 2011; Le, Kim et al. 2011) also have used the WHO scale.

The incidence of severe OM is planned as the single primary endpoint of this Phase 3 study. Secondary and exploratory endpoints are expected to provide important and clinically relevant data supporting the primary endpoint.

Because of the nature of the WHO grading scale (See Appendix 2), improvement of meaningful magnitude in the primary endpoint implies clinical benefit. While ulcerative (Grade 2) OM is very common, its functional consequences may be open to question. However, by definition, WHO Grade 3 OM entails a change in diet, and WHO Grade 4 OM includes inability to eat, requiring parenteral or tube feedings.

Many reports of OM in clinical studies use the NCI-CTCAE, in which the grading of OM is different from the WHO scale (Table 2). Whereas the WHO criteria have been stable for years, NCI criteria have changed markedly with every new version. The NCI criteria have found their greatest application in describing mucositis as an AE associated with anti-cancer regimens, not as an instrument to assess intervention efficacy.

Table 2: Comparison of WHO Scale vs. NCI-CTCAE v5.0 for Oral Mucositis

OM Grade	WHO Scale	NCI-CTCAE v5.0
0	No mucositis	No mucositis
1	Pain and erythema	Asymptomatic or mild symptoms; intervention not indicated
2	Ulceration but no compromise in diet (able to eat solid food)	Moderate pain or ulcer that does not interfere with oral intake; modified diet indicated
3	Ulceration with ability to eat only liquids	Severe pain; interfering with oral intake
4	Ulceration with inability to eat/requirement for tube or parenteral feeding	Life-threatening consequences; urgent intervention indicated

As noted, the WHO scale will be used in the current study. Separate grading of OM according to the NCI-CTCAE will not be performed. As in prior Galera-sponsored studies (GT-001 and GT-201), the WHO OM score for each subject will be assessed by trained Investigator-evaluators followed by Quality Control process as described under GT-201 results (See Investigator's Brochure).

6. INVESTIGATIONAL PLAN

6.1. Overall Study Design

GTI-4419-301 is a randomized, double-blind, placebo-controlled, multi-center international study conducted to evaluate GC4419 administered IV to reduce the incidence and severity of radiation induced OM in subjects receiving chemoradiation for SCCHN, limited to the oral cavity or oropharynx.

6.2. Treatment Plan and Duration of Therapy

Subjects will be randomized in a 3:2 ratio to 1 of 2 arms:

Arm A: 90 mg GC4419 per day (60 min IV infusion to complete within 60 minutes prior to IMRT), concurrent with daily fractions of IMRT (2.0-2.2 Gy) to a total of 60-72 Gy over approximately 7 weeks, plus cisplatin administered 100 mg/m^2 once every three weeks for 3 doses or 40 mg/m^2 once weekly for 6-7 doses (Investigator's choice)

Arm B: Placebo daily (60 min IV infusion to complete within 60 minutes prior to IMRT), concurrent with daily fractions of IMRT (2.0-2.2 Gy) to a total of 60-72 Gy over approximately 7 weeks, plus cisplatin administered 100 mg/m^2 once every three weeks for 3 doses or 40 mg/m^2 once weekly for 6-7 doses (Investigator's choice)

Enrollment will be stratified at randomization for 2 factors: cisplatin schedule (weekly vs q3week); post-operative vs definitive treatment.

Note: Planned radiation fields in both arms must include at least 2 oral sites (left and right buccal mucosa, floor of mouth, left and right ventral/lateral tongue, soft palate) with each site receiving a cumulative dose of at least 50 Gy.

GC4419/Placebo will be given IV by a 60-minute infusion. IMRT must be initiated as soon as possible upon completion of the GC4419/Placebo infusion but no later than 60 minutes following the end of the GC4419/Placebo infusion.

GC4419/Placebo will be given beginning on the first day of radiation and continuing daily, concurrent with each dose of IMRT, to a cumulative radiation dose of approximately 60-72 Gy.

If IMRT is not administered on any given day due to a treatment break or unforeseen circumstances, GC4419/Placebo should not be administered on that day. Breaks in IMRT will be determined by the subject's treating physician in accordance with standard of care. Subjects should resume GC4419/Placebo administration when IMRT resumes. On days when planned doses of both GC4419/Placebo and IMRT are not administered (e.g., due to a holiday site closure, etc.), GC4419/Placebo dosing may be extended along with IMRT to make up any missed dose up to a maximum of 35 doses of GC4419/Placebo. If a fraction of IMRT is not administered for any reason after GC4419/Placebo has been administered, that day's GC4419/Placebo will count as one of the 35 doses.

Anti-emetic prophylaxis and hematopoietic growth factor use should be administered per American Society of Clinical Oncology (ASCO) guidelines (Appendix 5). If institutional guidelines permit, cisplatin may be administered prior to or after the first day of IMRT, as long as it follows a weekly or tri-weekly schedule. On days in which chemotherapy and GC4419/Placebo are administered, the following administration sequence should be used if possible: GC4419/Placebo, IMRT, prehydration, and cisplatin. Patients treated with induction chemotherapy prior to concomitant chemoradiation are not eligible for this study.

All subjects will be assessed twice weekly for oral mucositis per WHO grading criteria until the end of the study treatment period (last day of IMRT). All subjects will continue to be assessed once weekly for 2 weeks after the end of the study treatment period.

The study will be monitored by a Data Monitoring Committee (DMC). Multinational Association of Supportive Care in Cancer (MASCC)/International Society of Oral Oncology (ISOO) Oral Care Education Materials and patient instructions for oral hygiene should be

strongly recommended. A summary of those guidelines and instructions is appended to the protocol (Appendix 6).

PK sampling to assess GC4419 will be sought from all subjects.

6.2.1. Rationale for GC4419 Dose and Schedule Selection

Data from the Phase 2 GT-201 study indicate that the acute toxicity of GC4419 and the overall adverse event profile in combination with IMRT/cisplatin are acceptable and as expected. Safety results with 90 mg dose were similar to those with Placebo and GC4419 does not appear to increase the toxicity of IMRT/cisplatin. In addition, the 90 mg dose of GC4419 in study GT-201 met its primary endpoint versus Placebo in demonstrating reduced duration of severe OM, and with strong statistical significance (p=0.024). Also of note is that Phase 2 data have indicated an apparent dose response between the 90 mg, 30 mg, and Placebo arms.

6.2.2. Rationale for Cisplatin/IMRT Treatment Plan

Meta-analyses have indicated superior survival for HNC patients treated with concurrent chemoradiotherapy compared with standard fractionation (M-F) radiation therapy alone. While not all available regimens have been tested directly against one another, the benefit appears superior for single-agent cisplatin over other chemotherapy regimens (Pignon, le Maitre et al. 2009). Further, recently reported results from the RTOG 1016 study in human papilloma virus (HPV)-associated oropharyngeal cancer showed survival after IMRT plus cetuximab to be inferior to that obtained with IMRT/cisplatin (Trotti, et al. 2018). Accordingly, in the current study, the still-standard cisplatin/IMRT regimen has been chosen as it poses a significant medical need by virtue of the predictable and high incidence of associated severe OM.

Standard fractionation (five fractions/week, delivered M-F) IMRT has been chosen for the current study. Although accelerated fractionation (six fractions/week, combined with two rather than three doses of cisplatin q3 weeks), has been studied, prospective lead investigators have advised that standard fractionation remains the widely-used standard, and in the interest of using a chemoradiation regimen that is as uniform and commonly-used as possible, the standard fractionation approach is being retained. Accelerated fractionation appears to increase the incidence of severe OM (Overgaard, Hansen et al. 2003).

In the event that a radiation fraction is missed because of a holiday or technical issues, administering two fractions in a subsequent day, to maintain five fractions per week, will be allowed, at the discretion of the treating investigator and consistent with institutional practice.

Cisplatin is administered concomitantly with IMRT by either a q3weekly or weekly schedule. Recent literature reviews (Sturtz, Wouters et al. 2017; Jacinto, Co, et al. 2017) have found no differences in tumor outcomes or mucositis between the two schedules. A randomized trial in the adjuvant (post-operative) setting (Noronha, Joshi, et al. 2018), in which 87% of patients enrolled had squamous cancer of the oral cavity, cited superior progression-free survival for the q3weekly arm, with no difference in mucositis. However, the weekly dose of cisplatin in that study was 30 mg/m^2. Other reports (Stojan, Vermorken et al. 2015; Nguyen-Tan, Zhang et al. 2014) have described a relationship between total cisplatin dose and overall survival in combination with RT for HNC, with a target cumulative cisplatin dose of 200 mg/m^2 or greater now appearing indicated as standard of care to obtain improved survival with RT/cisplatin over RT alone. This threshold could be missed with weekly cisplatin doses $<$ 40 mg/m^2. In the present trial, cisplatin doses of 100 mg/m^2 by the q3week schedule, and 40 mg/m^2 by the weekly

schedule, will be required. Although the risk of severe OM may not be different according to cisplatin schedule, and no such difference was demonstrated in a subset analysis from GT-201 (data not shown), stratification of randomization will be based on choice of cisplatin schedule, as it was in GT-201. Either regimen will be accepted as within the IMRT/cisplatin standard of care for the patient population to be enrolled in the present study.

6.2.3. Rationale for Tumor Follow-up and Analysis

Although the mechanism of action, existing nonclinical data (in vitro and in vivo), and existing clinical data with GC4419 uniformly indicate otherwise, there is a theoretical potential for GC4419 to protect the tumor from the therapeutic effects of concurrent chemoradiation. Patient candidates for GTI-4419-301 will be informed of this potential risk and the existing data as part of the consent process and document. GT-001 showed no evidence of tumor protection (See Investigator's Brochure). Tumor follow-up is ongoing for GT-201.

Fakhry et al. published an analysis comparing time to and patterns of progression for HPV-positive and HPV-negative patients treated in RTOG 0129 or RTOG 0522. The rate of progression was similar in the two groups. After a median follow-up of four years, the majority of progression events was found to have occurred in the first year after protocol therapy (Fakhry et al. 2014). This information suggests that tumor control data at one year after protocol treatment may provide a strong indication that results are consistent with expectations. As such, subjects in this Phase 3 trial will be followed for tumor progression for one year after completing treatment. Subjects will continue follow-up past that point for overall survival only.

7. SELECTION AND WITHDRAWAL OF SUBJECTS

Approximately 450 total subjects, 270 in the experimental arm (GC4419) and 180 in the control arm (Placebo), will be enrolled from investigational sites in the United States and Canada to ensure a sufficient number overall, with an assumed proportion of early discontinuations from OM assessments of approximately 10%.

7.1. Subject Inclusion Criteria

Patients are required to meet the following inclusion criteria before entering the study:

1. Pathologically-confirmed diagnosis of squamous cell carcinoma of the head and neck, defined as SCC of the oral cavity or oropharynx that will be treated with cisplatin plus concurrent IMRT.

Note: Patients with unknown primary tumors whose treatment plan matches the requirements specified in Inclusion Criteria #2 and #3 below are eligible for the study.

2. Treatment plan to receive a continuous course of IMRT delivered as single daily fractions of 2.0 to 2.2 Gy with a cumulative radiation dose of 60-72 Gy. Planned radiation treatment fields must include at least two oral sites (left and right buccal mucosa, floor of mouth, left and right ventral/lateral tongue, soft palate) that are each planned to receive a total of ≥ 50 Gy. Patients who have had prior surgery are eligible, provided they have fully recovered from surgery, and patients who may have surgery in the future are eligible.

Note: Unavoidable doses of at least 50 Gy, to include entrance, exit, and scatter doses, still constitute planned radiation.

3. Treatment plan to receive standard cisplatin monotherapy administered either every three weeks (100 mg/m^2 for 3 doses) or weekly (40 mg/m^2 for 6-7 doses). The decision on which cisplatin regimen to use in combination with IMRT and GC4419/Placebo will be at the discretion of the Investigator.
4. Age 18 years or older
5. Eastern Cooperative Oncology Group (ECOG) performance status ≤ 2
6. Adequate hematologic function as indicated by:
    - Absolute neutrophil counts (ANC) ≥ 1,500/mm^3
    - Hemoglobin (Hgb) ≥ 9.0 g/dL
    - Platelet count ≥ 100,000/mm^3
7. Adequate renal and liver function as indicated by:
    - Serum creatinine acceptable for treatment with cisplatin per institutional guidelines
    - Total bilirubin ≤ 1.5 × upper-normal limit (ULN)
    - Aspartate aminotransferase (AST) and alanine aminotransferase (ALT) ≤ 2.5 × ULN
    - Alkaline phosphatase ≤ 2.5 × ULN
8. Serum pregnancy test negative for females of childbearing potential
9. Males and females must agree to use effective contraception starting prior to the first day of treatment and continuing after the last dose of GC4419/Placebo for 30 days (females) or 90 days (males)
10. Properly obtained written informed consent

7.2. Subject Exclusion Criteria

Patients will be excluded if they meet any of the following exclusion criteria:

1. Tumor of the lips, larynx, hypopharynx, nasopharynx, sinuses, or salivary glands
2. M1 disease (distant metastasis)
3. Prior radiotherapy to the region of the study cancer or adjacent anatomical sites or more than 25% of total body marrow-bearing area (potentially interfering with chemo-tolerance)
4. Prior induction chemotherapy for current HNC or any prior immunotherapy
5. Receiving any approved or investigational anti-cancer agent other than those provided for in this study
6. Concurrent participation in another interventional clinical study or use of another investigational agent within 30 days of first dose of GC4419/Placebo

Note: Patients who are participating in non-interventional clinical studies (e.g., QOL, imaging, observational, follow-up studies, etc.) are eligible, regardless of the timing of participation.

7. Inability to eat soft solid food at baseline for reasons other than mouth soreness after surgery or dental procedures
8. Complete reliance on parenteral or gastrointestinal tube-delivered nutrition at baseline

Note: Patients who have gastrostomy tubes prophylactically placed are eligible. Patients receiving supplemental nutrition through a gastrostomy tube at baseline may be eligible depending on diet.

9. Malignant tumors other than HNC within the last 5 years, unless treated definitively and with low risk of recurrence in the judgment of the treating Investigator
10. Active infectious disease excluding oral candidiasis
11. Presence of oral mucositis at baseline. Subjects with mouth or throat pain solely due to post-operative effects are eligible, however.
12. Known history of human immunodeficiency virus (HIV) or history of active hepatitis B/C (patients who have been vaccinated for hepatitis B and do not have a history of infection are eligible)
13. Female patients who are pregnant or breastfeeding
14. Known allergies or intolerance to cisplatin and similar platinum-containing compounds
15. Requirement for concurrent treatment with nitrates or other drugs that may, in the judgment of the treating Investigator, create a risk for a precipitous decrease in blood pressure.
16. Medical history that includes any condition, or requires the use of concomitant medications which, in the Investigator’s judgment, are associated with or create a risk of increased carotid sinus sensitivity, symptomatic bradycardia, or syncopal episodes.

7.3. Rationale for Patient Population

Eligibility will be limited to patients with squamous cell tumors of the oral cavity or oropharynx in an attempt to keep the evaluation of OM as uniform as possible and to facilitate comparison with the prior data and assumptions as described in the Investigator’s Brochure. Although some studies, such as the studies of palifermin for OM in patients with HNC cancer, have included other anatomic sites (e.g., laryngeal, hypopharyngeal), exclusion of these from the current study may simplify the evaluation of OM (e.g., by reducing the need for endoscopy) for the immediate goal of obtaining a clearer assessment of the efficacy of GC4419 in the proposed indication. Patients with locally advanced squamous cell cancer of the oral cavity or oropharynx remain appropriate candidates for concurrent chemoradiation as standard of care, with single-agent cisplatin perhaps the most widely accepted and widely used standard regimen.

Eligibility will include patients scheduled to receive the prescribed IMRT/cisplatin regimen either as definitive therapy or post-operatively. Both groups may be candidates for IMRT/cisplatin as standard care and the incidence of severe OM is expected to be similar for both groups. In addition, both have been included in the Phase 1 study, GT-001 and the randomized Phase 2b study, GT-201.

Patients with unknown primary tumor whose IMRT/cisplatin treatment plans conform to study requirements will be eligible, provided that appropriate work up, including for tumor HPV status,

is consistent with an oropharyngeal or oral cavity primary site and IMRT/cisplatin treatment plans otherwise appropriate for the present study.

7.4. Screen Failures

A subject is considered to be a screen failure if the subject signs the informed consent form but withdraws consent or is deemed ineligible before being randomly assigned to a treatment arm. The reason why the subject was precluded from the clinical study will be collected. All subjects who sign the informed consent form for this study, including screening failures, will be entered in the IXRS.

7.5. Randomization Failures

A subject is considered to be a randomization failure if the subject signs the informed consent form and is randomized to a treatment arm but withdraws consent or is deemed ineligible prior to receiving their first dose of IMRT and GC4419/Placebo. Basic demographic and disease history information will be collected for randomization failures, as well of the reason the subject was precluded from the clinical study. All randomization failures will be listed on the Subject Status Log which is further detailed in the Regulatory Binder. Treatment assignment of randomization failures will remain blinded until after analysis of primary and secondary efficacy and safety data.

7.6. Subject Withdrawal Criteria

In accordance with the Declaration of Helsinki, a subject has the right to withdraw from the study at any time for any reason. The Investigator may also, at his/her discretion, discontinue a subject from participating in this study at any time. Additionally, study treatment may be discontinued for any of the following reasons:

- Adverse Event (AE)
- Medical requirement to administer a contra-indicated medication
- Subject non-compliance
- Subject has a confirmed positive serum pregnancy test
- Discontinuation of the study at the request of the Sponsor

The primary reason for ceasing treatment with the randomized therapy (GC4419 or Placebo) will be clearly documented in the subject's medical record and recorded on the appropriate CRF page. A subject who permanently discontinues treatment with GC4419 or Placebo will not be allowed to be retreated.

If a subject discontinues randomized therapy as a result of an AE or serious adverse event (SAE), every attempt should be made to keep the subject in the study and continue to perform the required study-related follow-up and procedures. If this is not possible or acceptable to the subject, the subject may be withdrawn from the study.

Subjects who withdraw consent for further administration of GC4419 or Placebo should be encouraged to continue and complete their standard treatment with IMRT/cisplatin, and should be encouraged to continue with other study procedures, notably with OM assessments as scheduled.

If a subject withdraws consent, additional details about the reasons for that decision will be sought and documented.

Withdrawn subjects will not be replaced.

7.7. Study and Site Closure

Both the Sponsor and the Investigator reserve the right to terminate the study at any time. Should this be necessary, both parties will arrange discontinuation procedures. In terminating the study, the Sponsor and the Investigator will assure that adequate consideration is given to the protection of the subjects' interests.

Upon completion of the study, the monitor will conduct the following activities in conjunction with the Investigator or site staff, as appropriate:

- Return of all study data to the Sponsor (as applicable)
- Resolution of all data queries
- Accountability, reconciliation, and arrangements for all unused GC4419/Placebo
- Review of site study records for completeness
- Shipment of laboratory samples (as applicable)

In addition, the Sponsor reserves the right to temporarily suspend or prematurely discontinue this study either at a single site or at all sites at any time for reasons including, but not limited to, safety or ethical issues or severe non-compliance. If the Sponsor determines such action is needed, the Sponsor will discuss this with the Investigator (including the reasons for taking such action) at that time. When feasible, the Sponsor will provide advance notification to the Investigator of the impending action prior to it taking effect.

The Sponsor will promptly inform all other investigators and/or institutions conducting the study if the study is suspended or terminated for safety reasons and will also inform the regulatory authorities of the suspension or termination of the study and the reason(s) for the action. If required by applicable regulations, the Investigator must inform the IRB/IEC/Research Ethics Board (REB) promptly and provide the reason for the suspension or termination. If the study is prematurely discontinued, all study data must be returned to the Sponsor.

Financial compensation to investigators and/or institutions will be in accordance with the agreement established between the Investigator and the Sponsor.

8. GC4419/PLACEBO

8.1. Description of GC4419/Placebo

8.1.1. GC4419

GC4419 (Manganese,dichloro[(4aS,13aS,17aS,21aS)-1,2,3,4,4a,5,6,12,13,13a,14,15,16,17,17a,18,19,20,21a-eicosahydro-11,7-nitilo-7H-dibenzo[b,h][1,2,7,10]tetraazacylcoheptadecine-κN5,κN13,κN18,κN21,κN22]-) is a water soluble, highly stable, low molecular weight manganese-containing macrocyclic ligand complex whose activity mimics that of naturally occurring SOD enzymes.

GC4419 is formulated as a clear solution at the following concentration to support the active arm:

Arm A: 90 mg GC4419: 9 mg/mL in 26 mM sodium bicarbonate-buffered 0.9 wt. % saline for parenteral administration.

There are no other excipients. GC4419 is packaged as a 11 mL ± 0.1mL aliquot in a 10 mL amber glass vial with an S-127 4432/50 gray stopper and a 20 mm red flip-off seal.

8.1.2. Placebo

Arm B: 26 mM sodium bicarbonate-buffered 0.9 wt. % saline for parenteral administration.

There are no other excipients. Placebo is packaged as a clear solution of 11 mL ± 0.1mL aliquot in a 10 mL amber glass vial, with an S-127 4432/50 gray stopper and a 20 mm red flip-off seal.

8.2. Treatment Assignment

Subjects will be randomly assigned to one of the two treatment arms through an interactive response technology (IRT). The system will assign a subject a unique randomization number that will remain consistent for the duration of the study. See the Pharmacy Binder for additional information regarding subject randomization and the IRT system.

8.2.1. Blinding

Treatment assignment should remain blinded until analyses of the primary and secondary safety and efficacy results of the study have been performed on the final, locked data through the end of post-IMRT follow-up for all subjects. Only in the case of an emergency, when knowledge of the investigational product is essential for the clinical management or welfare of the subject, may the Investigator unblind an individual subject's treatment assignment prior to the completion of the primary and secondary safety and efficacy analyses. The Investigator will, whenever possible, discuss options with the Medical Monitor or appropriate Sponsor/CRO study personnel before unblinding. If the blind is broken for any reason and the Investigator is unable to contact the Sponsor prior to unblinding, the Investigator must notify the Sponsor/CRO as soon as possible following the unblinding incident without revealing the subject's study treatment assignment, unless the information is important to the safety of subjects remaining in the study.

If a serious adverse event (SAE; as defined in Section 11.1.2) is reported to the Sponsor/CRO, the Sponsor/CRO staff may unblind the treatment assignment for the individual subject. If an expedited regulatory report to one or more regulatory agencies is required, the report will identify the subject's treatment assignment, consistent with applicable regulations for the territory in which the report is made. When applicable, a copy of the regulatory report may be sent to investigators in accordance with relevant regulations, the Sponsor policy, or both.

8.3. GC4419/Placebo Packaging and Labeling

GC4419 and Placebo will be presented in kits of 35 single-use vials, which represent 35 daily doses to be administered IV concurrent with IMRT. To maintain the blind, GC4419, as well as Placebo, will be packaged into amber vials and will appear generally identical; that is, there will be no distinguishing features in or on the packaging that could permit the identification of GC4419 or Placebo.

8.3.1. GC4419

GC4419 is packaged as an 11 mL ± 0.1mL aliquot in a 10 mL amber glass vial with a S-127 4432/50 gray stopper and a 20 mm red flip-off seal. Each bottle will be labeled with the appropriate language, including the required regulatory text. Further label details will be provided in a separate Pharmacy Manual.

8.3.2. Placebo

Placebo is packaged as an 11 mL ± 0.1mL aliquot in a 10 mL amber glass vial with an S-127 4432/50 gray stopper and a 20 mm red flip-off seal. Each bottle will be labeled with the appropriate language, including the required regulatory text. Further label details will be provided in a separate Pharmacy Manual.

8.4. GC4419/Placebo Storage

GC4419/Placebo must be stored at 2°C to 8°C at all times until use. Study drug must not be frozen at any time. Temperature excursions up to 25°C or down to 0.1°C for four hours are accepted; however, the Sponsor or its designee must be notified immediately of the temperature excursion to ensure proper oversight.

Once prepared, the IV bags containing GC4419/Placebo saline mixtures must be administered to subjects within 24 hours. GC4419/Placebo saline mixtures should spend as little time as possible outside refrigerated conditions, not exceeding more than 6 hours at ambient temperature, and must never be frozen. If freezing of the material is evident, that supply must be quarantined per institutional guidelines and the Sponsor or its designee must be notified immediately.

8.5. GC4419/Placebo Preparation

GC4419/Placebo will be provided to the study site in single use, sterile, pyrogen-free vials ready for dose preparation. Proper mixing with normal saline is required. Standard aseptic techniques will be used to maintain sterility.

Assignment of treatment arm will be randomized 3:2 with respect to treatment arms A and B. GC4419 and Placebo will be presented in kits of 35 single-use vials, which represent 35 daily doses to be administered IV concurrent with IMRT.

To prepare daily IV solutions, investigational pharmacists will extract 10 mL from a single vial and add to 250 mL normal saline. Note that there is no extraction of saline (i.e., the infusion solution volume will be 250 mL saline + 10mL volume of GC4419/Placebo for a total volume of 260 mL). No additional modifications or adjustments are to be made to the infusion solution.

The GC4419 solution may appear clear or have a slight yellowish tint. Although solutions should be free of particulates, it is possible that some vials may have some fine visible particulates. Infusions must be prepared using a sterile 0.2 or 0.22 micron syringe filter prior to introduction into the infusion bag or a sterile 0.2 or 0.22 micron inline filter must be used during IV administration. Filtration does not influence dosage calculations.

NOTE: Filtration is mandatory, either during preparation with syringe filters or at IV administration with inline filters. Unfiltered GC4419 solution or infusion solution must not be administered to patients.

Further information and preparation details will be provided in a separate Pharmacy Manual.

Note: Investigational staff who prepare infusion solutions cannot be oral evaluators. Any investigational staff that prepare infusions should maintain a strict blind of treatment assignment. In the event a vial contains particulates or is discolored with a slight yellowish tint that could indicate active drug, such an unblinded observation should not be discussed with internal site staff including those that may be directly involved in subject assessments.

8.6. GC4419/Placebo Administration

GC4419 or Placebo/saline mixture will be administered intravenously at an infusion rate that totals 60 min (± 6 min to account for saline overfill) for the total dose assigned. Infusions of GC4419/Placebo must be administered using an infusion pump (i.e., not by drip rate). Infusion pump models are not specified and may be per institutional preference/standard. If sterile 0.2 or 0.22 micron syringe filters are not used during infusion preparation, 0.2 or 0.22 micron inline filters must be used during IV administration.

To facilitate administration of GC4419/Placebo according to the study schedule, an indwelling venous access device may be used, at the discretion of the treating Investigator.

IMRT must be initiated as soon as possible upon completion of the GC4419/Placebo infusion, but no later than 60 minutes following the end of the infusion.

GC4419/Placebo will be given beginning on the first day of radiation and continuing daily, concurrent with each dose of IMRT, to a cumulative radiation dose of approximately 60-72 Gy.

Table 3 outlines the chemoradiation and GC4419/Placebo administration schedules. Please note chemotherapy is not required to be administered on the study days listed in Table 3 as long as it follows a weekly or tri-weekly schedule.

If IMRT is not administered on any given day due to a treatment break or unforeseen circumstances, GC4419/Placebo should not be administered on that day. Breaks in IMRT will be determined by the subject’s treating physician in accordance with standard of care. Subjects should resume GC4419/Placebo administration when IMRT resumes. On days when planned doses of both GC4419/Placebo and IMRT are not administered (e.g., due to a holiday site closure), GC4419/Placebo dosing may be extended along with IMRT to make up the missed dose(s) to a maximum of 35 doses of GC4419/Placebo. If a fraction of IMRT is not administered for any reason after GC4419/Placebo has been administered, that day’s GC4419/Placebo will count as one of the 35 doses.

**Table 3: Chemoradiation and GC4419/Placebo Administration Schedule
Example: 35 Doses of GC4419/Placebo (5 days/week over 7 Week Schedule)**

Treatment	Week 1					Week 2					Week 3					Week 4				
	Day 1	Day 2	Day 3	Day 4	Day 5	Day 8	Day 9	Day 10	Day 11	Day 12	Day 15	Day 16	Day 17	Day 18	Day 19	Day 22	Day 23	Day 24	Day 25	Day 26
GC4419/Placebo[1]	X	X	X	X	X	X	X	X	X	X	X	X	X	X	X	X	X	X	X	X
Cisplatin[2]																				
Tri-Weekly	X															X				
Weekly	X					X					X					X				
Radiation[3]	X	X	X	X	X	X	X	X	X	X	X	X	X	X	X	X	X	X	X	X

Treatment	Week 5					Week 6					Week 7				
	Day 29	Day 30	Day 31	Day 32	Day 33	Day 36	Day 37	Day 38	Day 39	Day 40	Day 43	Day 44	Day 45	Day 46	Day 47
GC4419/Placebo[1]	X	X	X	X	X	X	X	X	X	X	X	X	X	X	X
Cisplatin[2]															
Tri-Weekly											X				
Weekly	X					X					X[4]				
Radiation[3]	X	X	X	X	X	X	X	X	X	X	X	X	X	X	X

[1] Intravenous GC4419/Placebo is administered by a 60-minute intravenous infusion (±6 min) once a day for the first 35 days of IMRT (Monday through Friday). IMRT must be initiated as soon as possible upon completion of GC4419/Placebo infusion, but no later than 60 minutes post GC4419/Placebo infusion. On Day 1/Baseline, chemotherapy prehydration and infusion should be administered after GC4419/Placebo infusion and IMRT, if possible. If IMRT is not received on any given day due to a treatment break or unforeseen circumstances, GC4419/Placebo should not be administered on that day. Subjects should resume GC4419/Placebo administration when IMRT resumes. If a subject is scheduled to receive IMRT on a weekend day (e.g., to make-up for a holiday site closure), the Investigator should contact the Medical Monitor for a discussion prior to IMRT administration.

[2] Cisplatin monotherapy should be administered in a standard q3 weeks regimen (100 mg/m^2) or weekly regimen (40 mg/m^2). Anti-emetic prophylaxis and hematopoietic growth factor use should be administered per ASCO guidelines. If institutional guidelines permit, cisplatin may be administered prior to or after the first day of IMRT. On days in which chemotherapy and GC4419/Placebo are administered, the administration sequence should be GC4419/Placebo, IMRT, prehydration, and then cisplatin, if possible. Patients treated with induction chemotherapy prior to concomitant chemoradiation are not eligible for this study.

[3] Eligible patients will be scheduled to receive a continuous course IMRT delivered in single daily fractions of 2.0 to 2.2 Gy, five days per week (Monday through Friday), with a cumulative radiation dose of 60-72 Gy. Planned radiation treatment fields must include at least two oral sites (buccal mucosa, floor of mouth, tongue, soft palate) that are each planned to receive $\geq$ 50 cumulative Gy.

[4] The 7th dose of cisplatin will only be administered if the subject is on the 7-dose weekly cisplatin schedule (weekly cisplatin schedule may be 6 or 7 doses, depending on Investigator choice).

8.7. GC4419/Placebo Accountability and Compliance

Compliance with GC4419/Placebo dosing, including administration details (e.g., volume, start, stop times, etc.) should be documented in the source documents and recorded on the CRF.

The Investigator is responsible for ensuring adequate accountability of all used and unused GC4419/Placebo. This includes acknowledgment of receipt of each shipment of GC4419/Placebo (quantity and condition), subject dispensing records, and quantity of GC4419/Placebo returned or destroyed. Dispensing records will document quantities received from the Sponsor and quantities dispensed to subjects, including container number or lot number, date dispensed, subject identifier number, subject initials, and the initials of the person dispensing the medication. Any GC4419/Placebo that is prepared but not used must also be recorded in the dispensing records.

All GC4419/Placebo supplies and associated documentation will be reviewed and verified by the study monitor. All GC4419/Placebo and used containers are to be retained by the site until notified by the study monitor, who will instruct the site in the disposal and/or destruction of all used GC4419/Placebo supplies. Copies of all forms, documenting drug receipt at the study site, drug transportation to satellite sites, and drug return to the Sponsor, together with drug accountability records, will be retained according to the regulations governing record retention.

The Investigator will not allow GC4419/Placebo to be given to any patient not included in the study or to any unauthorized person.

8.8. GC4419/Placebo Handling and Disposal

After completion of the study, all unused GC4419/Placebo will be inventoried and, if possible, destroyed locally at the site. GC4419/Placebo should not be returned directly to the Sponsor unless specifically requested by the Sponsor. The study monitor will instruct the site in the disposal and/or destruction of all used and unused GC4419/Placebo supplies. Destruction of any GC4419/Placebo should be documented appropriately.

8.9. Concomitant Medications

All concomitant therapies (i.e., prescription and over-the-counter medications) taken by subjects from the date of randomization through 30 days following the last GC4419/Placebo, IMRT or cisplatin (i.e., whichever occurs last) dose will be collected in the CRF. Additionally, any concomitant therapies if used to treat any serious or related adverse event will be recorded in the CRF.

Anti-emetic prophylaxis and hematopoietic growth factors should be used per ASCO guidelines (Hesketh, Kris et al. 2017).

Subjects who withdraw consent for GC4419/Placebo should be encouraged to continue and complete standard IMRT/cisplatin treatment and other protocol procedures, notably scheduled OM assessments.

However, if a subject withdraws consent for the study or is removed from the study completely (i.e., the subject is no longer participating in any study procedures or follow-up) no further data should be collected after the date of the subject’s study discontinuation.

8.9.1. Prohibited Medications

Investigators may prescribe any concomitant medication or supportive therapy deemed necessary to provide adequate supportive care including antiemetics, systemic antibiotics, hydration to prevent renal damage, topical fluoride etc., with the following exceptions:

- Low-level laser treatment for OM
- Amifostine (Ethyol®)
- Benzydamine (Difflam®, Pharixia®, Tantum Verde)
- Cetuximab (Erbitux®)
- Glutamine in any form
- GM-CSF applied topically
- ‘Magic mouthwashes’ or ‘Miracle mouthwashes’ are permitted, provided they do not contain:
  - Chlorhexidine
  - Hydrogen peroxide
  - Diphenhydramine (Benadryl®) liquid formulation
  - Tetracycline
  - Any other listed disallowed medications
  - Notes:
    - Topical lidocaine preparations are permitted
    - Diphenhydramine (Benadryl®) administered as tablets or by injection is permitted
- MuGard™, Gelclair®, Episil®, or other barrier devices
- Caphosol®
- Nitrates, phosphodiesterase type 5 (PDE 5) inhibitors (e.g., sildenafil, tadalafil, or similar agents) or other drugs that in the judgment of the treating Investigator could create a risk of a precipitous decrease in blood pressure are prohibited until at least 24 hours after the last dose of GC4419
- Pyridostigmine or other drugs that in the judgment of the treating Investigator could create a risk of increased carotid sinus sensitivity, symptomatic bradycardia, or syncopal episodes are prohibited until at least 24 hours after the last dose of GC4419
- Palifermin (Kepivance®) or other keratinocyte or fibroblast growth factor
- Povidone-iodine rinses
- Steroid rinses
- Sucralfate in suspension form (use of sucralfate tablets is permitted)

Other biologic response modifiers – except systemic hematopoietic growth factors for the management of anemia or myelosuppression

Concurrent approved or investigational anti-cancer therapy (e.g., chemotherapy, immunotherapy, targeted therapy, hormone and biologic therapy) other than the Protocol regimen

Other investigational agents or perceived herbal/homeopathic or other remedies for OM, which are considered investigational for purposes of this study (includes nitric oxide)

All medication restrictions begin on Day 1 of IMRT and GC4419/Placebo. All medication restrictions end after post-IMRT OM follow-up is completed unless otherwise noted.

Subjects who receive prohibited medications prior to completion of post-IMRT OM follow-up will not automatically be removed from the study; however, administration of a prohibited medication is a significant deviation from the protocol and must be reported to the Medical Monitor as soon as possible and the presiding IRB/IEC/REB (per institutional guidelines). The decision for study continuation or discontinuation will be made at that time on a case-by-case basis and in consideration of the clinical requirement and circumstances.

Although unlikely to affect OM, diphenhydramine (Benadryl®) should not be used in mouthwashes or rinses.

Chlorhexidine oral rinse may exacerbate OM and should not be used.

Mouthwashes or rinses containing sodium bicarbonate, clotrimazole (Mycelex), nystatin, fluconazole (Diflucan), viscous xylocaine, and/or viscous lidocaine are permitted. If a subject uses “Magic Mouthwashes” or “Miracle Mouthwashes,” all ingredients must be recorded in the subject’s medical record in order to confirm the mouthwashes did not contain the prohibited ingredients listed above.

Anti-emetic prophylaxis and hematopoietic growth factor use are permitted per ASCO guidelines. Following ASCO (and MASCC) guidelines for the prevention and management of chemotherapy-induced nausea and vomiting (CINV) is strongly encouraged.

Oral care per International Society of Oral Oncology (ISOO) guidelines is strongly recommended for all subjects as part of standard of care (Appendix 6).

8.9.2. CYP2D6 Substrates

In vitro, GC4419 inhibited cytochrome P450 isozyme 2D6 (CYP2D6) with an IC50 of 0.079 μM. Other cytochrome P450 isozymes were not inhibited in vitro. This prompted a clinical drug-drug interaction study in healthy human subjects, which demonstrated that GC4419 is a strong inhibitor of CYP2D6.

Concomitant use of GC4419 increases the concentration of drugs that are CYP2D6 substrates, which may increase the risk of toxicities of these drugs. Concomitant use of GC4419 with CYP2D6 substrates with a narrow therapeutic range should be considered carefully (CYP2D6 substrates where minimal increases in concentration of the substrate may lead to serious or life-threatening toxicities). Such CYP2D6 substrates include certain beta blockers (e.g., propranolol and metoprolol), antidepressants (e.g., tricyclics), antipsychotics (e.g., phenothiazines and most atypicals), and antiarrhythmics (e.g., propafenone, flecainide) (see Appendix 7 for additional examples).

In addition, concomitant use of GC4419 may decrease the concentration of active metabolites of prodrugs that require CYP2D6 for activation. Concomitant use of GC4419 with CYP2D6 prodrug substrates requiring functional CYP2D6 activity for their clinical benefit (e.g., codeine, tramadol, taxomifen) may decrease their effectiveness.

Concomitant administration of a CYP2D6-substrate drug concurrent with administration of GC4419 should be limited to cases in which the treating investigator has determined that the use of the specific CYP2D6-substrate drug is medically necessary. In such cases, the patient should be carefully observed for clinical adverse events of the CYP2D6-substrate drug, the dose of which may require reduction.

9. TOXICITY MANAGEMENT

9.1. Dose Delays and Dose Modifications for Toxicity

The following toxicity requires a 25% dose reduction in GC4419/Placebo:

Grade 2 or greater hypotension within 2 hours after the start of GC4419/Placebo infusion (i.e., anytime from start of infusion through 1 hour after end of infusion).

The dose of GC4419 or Placebo may be reduced by 25% for Grade 3-4 adverse events (AEs) judged by the Investigator to be likely attributable to the study infusion.

Two dose reductions for toxicity will be permitted per subject. After the first event, the subject will be re-challenged at 75% of the original dose (7.5 mL GC4419/Placebo in 250 mL normal saline). After the second event, the subject will be re-challenged at 50% of the original dose (5.0 mL GC4419/Placebo in 250 mL normal saline). Subjects who are unable to tolerate GC4419/Placebo infusions following 2 dose reductions must be discontinued from the study treatment but should continue with cisplatin/IMRT and other study assessments and procedures, with the concurrence of the treating Investigator, if the subject maintains informed consent to do so.

For other toxicities (including those attributable to cisplatin and IMRT), management will be per institutional and ASCO guidelines and Investigator judgment.

The Sponsor strongly recommends managing treatment modifications for cisplatin related toxicities by reducing the dose and/or altering the schedule of cisplatin administration. Such modifications may be made per the judgment of the treating Investigator. However, substitution of other systemic agents (e.g., carboplatin with or without paclitaxel, cetuximab, etc.) is not consistent with the protocol and should not be done.

OM will not be considered an AE requiring dose modification for the purposes of this study.

9.2. Supportive Care Guidelines

Necessary supportive measures for optimal medical care will be given throughout the study. Supportive care medications may be administered at the Investigator's discretion and recorded in the CRF (including administration of prophylactic antiemetic medication if deemed appropriate by the Investigator). However, medications are subject to the exclusions listed in Section 8.9.1.

9.2.1. Supportive care for chemotherapy-induced nausea and vomiting (CINV)

Medication to prevent or manage chemotherapy-induced nausea and vomiting (CINV) should follow recent guidelines from ASCO and MASCC (Appendix 5). Adult patients who are treated with cisplatin (considered a high-emetic-risk single agent for the purposes of these guidelines) should be offered a four-drug combination of a NK_1 receptor antagonist, a serotonin (5-HT_3) receptor antagonist, dexamethasone, and olanzapine. Dexamethasone and olanzapine should be continued on days 2 to 4. (Type: evidence based, benefits outweigh harms; quality of evidence: high; strength of recommendation: strong.) (Hesketh, Kris et al. 2017).

Supportive care for CINV should be optimized, per ASCO and MASCC guidelines, before GC4419 dose is reduced for nausea and vomiting.

10. ASSESSMENTS

The study procedures to be conducted for each subject enrolled in the study are described in the text that follows and presented in the Schedule of Assessments in Appendix 1.

Any deviation from protocol procedures should be explained in the source documents. The Sponsor (or designee) and the site's institutional review board (IRB) – as required by the IRB's policies and procedures – should be notified as soon as possible of any deviations potentially affecting subject safety, GC4419/Placebo administration or the assessment of safety, efficacy and tolerability parameters.

10.1. Safety Assessments

Safety will be assessed on the basis of treatment-emergent AEs, physical examination findings, clinical laboratory tests, electrocardiogram (ECG) measurements, and vital sign measurements.

10.1.1. Clinical Assessments

The following clinical assessments are defined when referenced in the schedule of events for this study:

12-Lead ECG: ventricular rate, P-R interval, QRS interval, QT interval, and QTc

Vital signs: measured following two minutes of rest in the sitting position – temperature, systolic and diastolic blood pressures, heart rate and respiration rate

Weight and Height: measured in kilograms (kg) and centimeters (cm), respectively

Performance Status: ECOG (see Appendix 3 for conversion criteria for Karnofsky to ECOG)

Vital signs are required to be taken at Screening, Day 1 (Baseline), once during Week 4, once during Week 7 and any additional IMRT weeks, and on the Last Day of IMRT. On Day 1 (Baseline) and Day 22, blood pressure must be measured at the following times:

1. Within 30 minutes prior to the pre-GC4419/Placebo PK draw, and
2. After GC4419/Placebo infusion, prior to standing and prior to the End of GC4419/Placebo Infusion PK draw.

10.1.2. Laboratory Assessments

All protocol required clinical laboratory assessments from screening through end of treatment should be performed at the central laboratory.

The Investigator must assess all abnormal clinical laboratory results for clinical significance in a timely fashion. A notation of clinically significant (CS) or non-clinically significant (NCS) with initials and date will be documented on the respective laboratory report next to any abnormal value. Information on laboratory AE reporting can be found in Section 11.2.

The following laboratory assessments are defined when referenced in the schedule of events (Table 6) for this study:

Hematology Profile: hemoglobin, hematocrit, red blood cell count, white blood cell count with differential, and platelet count. Differential to include total neutrophils, lymphocytes, monocytes, eosinophils, and basophils.

Serum Chemistry Profile: glucose, blood urea nitrogen (BUN), creatinine, sodium, potassium, calcium, albumin, total protein, direct bilirubin, total bilirubin, alkaline phosphatase, ALT (SGPT), AST (SGOT), chloride, phosphate, bicarbonate, magnesium.

Serum Cotinine Test: required for all subjects at Baseline, Week 4, and Last Day of IMRT.

Serum Pregnancy Test: required for all females of childbearing potential. Lack of childbearing potential must be noted in the source documents, if applicable.

Blood urea nitrogen (BUN) and creatinine measurements will also be taken at 3, 6, 9, and 12-months post-IMRT to assess kidney function. These assessments will be done at local labs.

10.2. Oral Mucositis Assessments

OM assessments will be completed at the Screening Visit (within 28 days of IMRT start), Baseline Visit, and twice weekly (no less than two days apart) within each five-day IMRT treatment period. The extent of subjects’ OM will be assessed by a trained evaluator and scored using the WHO OM toxicity grading scale. All subjects must have an oral assessment on the last day of IMRT treatment. If a subject withdraws for any reason prior to the end of IMRT, a complete oral assessment should be done on that day.

All subjects will be evaluated at 7 ± 2 calendar days from the last day of IMRT (Post-IMRT Week 1) and 14 ± 2 calendar days from the last day of IMRT (Post-IMRT Week 2).

Study site personnel will be provided with specific training and instructions regarding OM assessment performance, grading, and documentation. Designated trained study staff (oral evaluators) will conduct all assessments using a standardized and consistent method. To reduce inter-observer variability, the fewest possible number of evaluators should be involved in the assessments of each subject. The oral evaluators will use a Sponsor-provided headlamp for all oral assessments conducted for this study.

The WHO scale will be the measure for assessing OM. The assessment of the impact of OM on a subject’s ability to eat is critical for accurate scoring of the WHO scale. Therefore, standardization of the assessment is very important. In order to reduce variability in assessing food intake, the definitions for solids, liquids, and nothing by mouth are provided here:

Solid foods are defined as foods that need to be chewed. Examples include meat, grains and vegetables.

Liquids are defined as foods that take the shape of their container. Examples include fruit juices, soups, pureed foods, mashed potatoes, cooked cereals (oatmeal), baby food, Jell O®, pudding, and ice cream.

Nothing by mouth is defined as no eating or drinking, except enough liquid to allow for taking medications.

The WHO scoring scale is appended in Appendix 2.

10.3. Radiation Therapy Quality Assurance

Quality assurance for IMRT will be conducted by a prospective review of the overall treatment plan, at the time of subject screening, by a radiation oncologist who is not an Investigator on the study. In addition to IMRT treatment, dosimetry and dose volume histograms will be collected after simulation. The 50-Gy isodose line should be clearly indicated, along with relevant imaging through the anatomic region for planned treatment. Confirmation of appropriate planned doses to oral cavity and oropharynx sub-sites for eligibility will be confirmed.

This information will be available for summary and retrospective analysis at the end of the study.

10.4. Pharmacokinetic (PK) Measurements

10.4.1. Pharmacokinetic (PK) Measurements of GC4419

PK sampling will be sought from all subjects for GC4419.

Plasma samples for GC4419 PK measurements will be collected in two cycles: Study Days 1 and 2, and on Study Day 22. On Study Days 1 and 22, four samples will be drawn as follows:

The first sample will be drawn prior to GC4419/Placebo administration.

The second sample will be drawn within 10 minutes after the end of GC4419/Placebo infusion.

The third sample will be drawn within 10 minutes after the end of IMRT.

The fourth and final sample will be drawn between 60 and 180 minutes after the end of GC4419/Placebo infusion.

On Study Day 2 only one PK sample will be drawn. This sample should be drawn prior to the GC4419/Placebo infusion on Day 2.

Table 4 below summarizes the PK sample collection time points. Note that this schedule may be adjusted to accommodate mid/late-week study starts and holidays. Adjustments in the PK schedule must be discussed with the Sponsor or its representative ahead of time. Time of actual blood draws for PK assessment must be recorded in the source notes. Plasma PK samples will be sent to a central laboratory for analysis and interpretation. Further details on PK sample collection, processing, and shipping are provided in a separate manual. Volumes and start/stop times for administration of IV fluids will be collected on PK sampling days.

Table 4: Pharmacokinetic Sampling Schedule to Assess GC4419

Timepoint	Day 1	Day 2	Day 22
Pre-GC4419/Placebo	X	X	X
End of GC4419/Placebo Infusion (+10min)	X		X
Post-IMRT (+10min)	X		X
60-180 min Post End of GC4419/Placebo Infusion	X		X

10.5. Biological Surrogate Markers of Mucositis

Biological surrogate markers of mucositis will be assessed in all subjects enrolled in the study. Correlation between levels of circulating cytokines and proteins and clinical endpoints will be assessed by analysis of blood samples collected for all subjects during the treatment phase of the study. Refer to Schedule of Assessments in Appendix 1, Table 6 for the schedule of biomarker assessments for each treatment schedule. For subjects who consent separately, RNA samples for the assessment of gene expression patterns prior to receiving the first dose of GC4419 and upon completion of GC4419 doses will be collected during screening and on the last day of IMRT. Additional details regarding the processing and handling of biomarker samples will be provided in a separate manual.

Blood draws for cytokine analysis should be conducted on an OM assessment day. For standardization, the Sponsor recommends conducting the cytokine draw on the day of the second OM assessment.

10.6. Tumor Status Assessment

10.6.1. Clinical Tumor Assessment

The subject’s tumor status will be assessed clinically at the following time points:

- Last day of IMRT
- Every 3 months, ± 30 days, throughout the 1st year post-IMRT (Months 3, 6, 9, and 12)

The following tumor status information will be collected at each of the above time points:

- Disease progression – locoregional and distant metastasis
- Development of second primary tumors
- Additional malignancies

A head/neck/oral exam to assess the tumor status is sufficient on the Last day of IMRT if disease progression is not suspected. If disease progression is suspected, a laryngopharyngoscopy should be conducted on the Last day of IMRT.

At Months 3, 6, 9, and 12 post-IMRT, a brief history & a physical examination by a Radiation Oncologist, Medical Oncologist, ENT, or Head & Neck Surgeon, including a laryngopharyngoscopy (mirror and/or fiberoptic and/or direct procedure), must be conducted. If local/regional progression or recurrence has already been discovered at a previous visit, a laryngopharyngoscopy is no longer required at future visits. However, if distant progression is

discovered at a previous visit, a laryngopharyngoscopy is still required at subsequent visits to assess the subject for local/regional progression/recurrence. A head/neck/oral exam must always be conducted at months 3, 6, 9, and 12 post-IMRT.

10.6.2. Tumor Imaging

10.6.2.1. Pre-Treatment Tumor Imaging

Radiographic tumor imaging must occur within 60 days prior to the first day of IMRT (Baseline) for definitively treated subjects. Pre-surgical imaging should be used if available for post-operatively treated subjects, even if the imaging occurs prior to the 60-day window. If pre-surgical imaging was not done, post-surgical imaging can be used.

One of the following imaging combinations is recommended:

- CT scan of the neck with contrast plus chest CT with or without contrast
- MRI scan of the neck with contrast plus chest CT with or without contrast
- CT scan of the neck with contrast plus PET/CT of the neck and chest with or without contrast
- MRI scan of the neck with contrast plus PET/CT of the neck and chest with or without contrast

If the subject has no evidence of disease at Baseline, it should be clearly indicated.

10.6.2.2. Post-Treatment Tumor Imaging

Radiographic imaging must be performed at Month 12 post-IMRT for all subjects. Subjects treated definitively (as opposed to post-operatively) will also undergo imaging at Month 3 post-IMRT to assess tumor response/clearance to the degree possible.

Radiographic imaging is highly recommended at any post-IMRT follow-up visit at which disease progression is suspected by the treating physician. If radiographic imaging is performed, both local/regional recurrence and distant metastases should be evaluated.

When possible, the same imaging technique (i.e., CAT, PET, or MRI) that was used for pre-treatment tumor imaging at the time of staging should be used for post-treatment tumor imaging.

10.6.3. Survival Status

Subjects will be followed for survival for two years after IMRT:

- Every 3 months, ± 30 days, throughout the 1st year post-IMRT (Months 3, 6, 9, and 12)
- Every 4 months, ± 30 days, throughout the 2nd year post-IMRT (Months 16, 20, and 24)

10.7. Schedule of Time and Events

A schedule of study assessments table is located in Appendix 1, Table 6. Minor changes to the assessment schedule may be made to accommodate holidays, administrative closures, etc., which if necessary, are not considered deviations by the Sponsor. Sites should contact the Sponsor (or its representative) prospectively to address rescheduling protocol assessments and data handling.

10.8. Screening

The following screening observations and procedures will be completed within 28 days of IMRT and GC4419/Placebo start:

Obtain a signed IRB/IEC/REB-approved informed consent form (ICF)

Confirm subject eligibility by reviewing inclusion/exclusion criteria

Obtain medical history, tobacco use history, and alcohol use history

Obtain HNC history; HNC history should include:

- Tumor HPV status, and strain(s) if known
- Pre-treatment tumor imaging (See Section 10.6.2.1)
- Prior treatments
- Confirmation of histopathological diagnosis of SCC
- Tumor staging (AJCC 8th Edition) (pre-surgical staging for surgical subjects if available; otherwise, provide post-surgical staging)

Conduct a complete physical examination, including height

Ensure a dental examination was conducted for IMRT clearance and potential sources of mucosal irritation (e.g., tooth extraction) were eliminated. The dental exam must occur within the 28-day screening period. The exam must be performed by a licensed clinician, but not necessarily by a dentist. For edentulous subjects, the subject must be cleared for IMRT per SOC (i.e., oral exam).

Conduct OM assessment and record the WHO score

Record planned IMRT and chemotherapy parameters

Measure vital signs, body weight, and ECOG Performance Status

Conduct a 12-lead ECG

Record concomitant medications from date of randomization

Record AEs from date of randomization

Record/update medical conditions and illnesses that have occurred since the subject signed the ICF and record in medical history

Draw blood for laboratory measurements

- Chemistry profile
- Hematology profile
- Biomarker sample for pro-inflammatory cytokine analysis (See Section 10.5)
- For subjects who consent separately: RNA sample (PAXgene) for genomic studies (See Section 10.5)
- Serum pregnancy test for women of childbearing potential

All questions related to subject eligibility should be directed to Galera's Medical Monitor or designee.

10.9. Treatment

10.9.1. Baseline/Day 1 (First Day of IMRT and GC4419/Placebo)

Prior to receiving the first dose of GC4419/Placebo the following observations and procedures will be conducted for all subjects:

Confirm continued subject eligibility by reviewing inclusion/exclusion criteria

Measure vital signs, body weight, and ECOG Performance Status

Blood pressure must be measured within 30 minutes prior to pre-GC4419/Placebo Infusion PK blood draw (See Section 10.1.1)

Record BSA (used to confirm cisplatin dosing)

Ensure concomitant medications have been recorded from date of randomization

Ensure AEs have been recorded from date of randomization

Record/update medical conditions and illnesses that have occurred since the subject signed the ICF and record in medical history.

Record tobacco use

Conduct first OM assessment for the week and record the severity using the WHO score

Draw blood for laboratory measurements

- Chemistry profile
- Hematology profile
- Cotinine test
- PK sampling (See Section 10.4)
- Biomarker sample for pro-inflammatory cytokine analysis (See Section 10.5)

Administer the first GC4419/Placebo dose by continuous intravenous infusion over 60 minutes.

Following GC4419/Placebo administration the following observations and procedures will be conducted for all subjects:

Measure blood pressure prior to End of GC4419/Placebo Infusion PK draw (See Section 10.1.1)

Draw blood for PK sampling (See Section 10.4)

Administer IMRT as soon as possible but no later than 60 minutes following the end of GC4419/Placebo dosing.

10.9.2. Day 2 of IMRT and GC4419/Placebo

Prior to GC4419/Placebo administration the following observations and procedures will be conducted for all subjects:

Record changes to concomitant medications

Record AEs

PK sampling (Section 10.4)

Administer the second GC4419/Placebo dose by continuous intravenous infusion. IMRT must begin as soon as possible but no later than 60 minutes following the end of GC4419/Placebo dosing.

10.9.3. Days 3 to 5 of IMRT and GC4419/Placebo

Prior to GC4419/Placebo administration the following observations and procedures will be conducted for all subjects on Days 3, 4, and 5 unless otherwise noted:

Record changes to concomitant medications

Record AEs

Prior to GC4419/Placebo administration the following observations and procedures will be conducted for all subjects on Day 3, 4, or 5 unless otherwise noted:

Conduct second OM assessment for the week and record the severity using the WHO score

Draw blood for laboratory measurements

- Chemistry profile
- Hematology profile

Administer GC4419/Placebo dose by continuous intravenous infusion. IMRT must begin as soon as possible but no later than 60 minutes following the end of GC4419/Placebo dosing.

10.9.4. Week 2

Prior to GC4419 administration the following observations and procedures will be conducted for all subjects on all IMRT days:

Record changes to concomitant medications

Record AEs

Prior to GC4419/Placebo administration the following procedure will be conducted for all subjects twice during this week, at least two days apart:

Conduct OM assessment and record the severity using the WHO score

Prior to GC4419/Placebo administration the following observations and procedures will be conducted for all subjects once this week:

For subjects receiving weekly cisplatin, record BSA (used to confirm cisplatin dosing)

Measure body weight

Record tobacco use

Draw blood for laboratory measurements

- Chemistry profile

- Hematology profile
- Biomarker sample for pro-inflammatory cytokine analysis (See Section 10.5)

Administer GC4419/Placebo dose by continuous intravenous infusion. IMRT must begin as soon as possible but no later than 60 minutes following the end of GC4419 dosing.

10.9.5. Week 3

Prior to GC4419/Placebo administration the following observations and procedures will be conducted for all subjects on all IMRT days:

Record changes to concomitant medications

Record AEs

Prior to GC4419/Placebo administration the following procedure will be conducted for all subjects twice during this week, at least two days apart:

Conduct OM assessment and record the severity using the WHO score

Prior to GC4419/Placebo administration the following observations and procedures will be conducted for all subjects once this week:

For subjects receiving weekly cisplatin, record BSA (used to confirm cisplatin dosing)

Measure body weight

Record tobacco use

Draw blood for laboratory measurements

- Chemistry profile
- Hematology profile

Administer GC4419/Placebo doses by continuous intravenous infusion. IMRT must begin as soon as possible but no later than 60 minutes following the end of GC4419 dosing.

10.9.6. Week 4

Prior to GC4419/Placebo administration the following observations and procedures will be conducted for all subjects on all IMRT days:

Record changes to concomitant medications

Record AEs

Prior to IMRT administration the following procedure will be conducted for all subjects twice during this week, at least two days apart:

Conduct OM assessment and record the severity using the WHO score

Prior to GC4419/Placebo administration the following observations and procedures will be conducted for all subjects once this week:

Perform symptom-directed PE

Measure vital signs, body weight, and ECOG Performance Status

Record BSA (used to confirm chemotherapy dosing)

Record tobacco use

Draw blood for laboratory measurements

- Chemistry profile
- Hematology profile
- Cotinine test
- Biomarker sample for pro-inflammatory cytokine analysis (See Section 10.5)

Prior to GC4419/Placebo administration the following observations and procedures will be conducted for all subjects on Day 22 only:

Draw blood for PK sampling (See Section 10.4)

Measure blood pressure within 30 minutes prior to pre-GC4419/Placebo Infusion PK blood draw (See Section 10.1.1)

Administer GC4419/Placebo doses by continuous intravenous infusion.

Following GC4419/Placebo administration the following observations and procedures will be conducted for all subjects on Day 22 only:

Measure blood pressure (See Section 10.1.1)

Draw blood for PK sampling (See Section 10.4)

IMRT must begin as soon as possible but no later than 60 minutes following the end of GC4419 dosing.

10.9.7. Week 5

Prior to GC4419/Placebo administration the following observations and procedures will be conducted for all subjects on all IMRT days:

Record changes to concomitant medications

Record AEs

Prior to GC4419/Placebo administration the following procedure will be conducted for all subjects twice during this week, at least two days apart:

Conduct OM assessment and record the severity using the WHO score

Prior to GC4419/Placebo administration the following observations and procedures will be conducted for all subjects once this week:

For subjects receiving weekly cisplatin, record BSA (used to confirm cisplatin dosing)

Measure body weight

Record tobacco use

Draw blood for laboratory measurements

- Chemistry profile

   - Hematology profile

Administer GC4419/Placebo doses by continuous intravenous infusion. IMRT must begin as soon as possible but no later than 60 minutes following the end of GC4419 dosing.

10.9.8. Week 6

Prior to GC4419/Placebo administration the following observations and procedures will be conducted for all subjects on all IMRT days:

Record changes to concomitant medications

Record AEs

Prior to IMRT administration the following procedure will be conducted for all subjects twice during this week, at least two days apart:

Conduct OM assessment and record the severity using the WHO score

Prior to IMRT administration the following observations and procedures will be conducted for all subjects on the once this week:

For subjects receiving weekly cisplatin, record BSA (used to confirm cisplatin dosing)

Measure body weight

Record tobacco use

Draw blood for laboratory measurements

   - Chemistry profile
   - Hematology profile
   - Biomarker sample for pro-inflammatory cytokine analysis (See Section 10.5)

Administer GC4419/Placebo doses by continuous intravenous infusion. IMRT must begin as soon as possible but no later than 60 minutes following the end of GC4419 dosing.

10.9.9. Week 7 (plus additional IMRT weeks, if needed)

Prior to GC4419/Placebo administration the following observations and procedures will be conducted for all subjects on all IMRT days:

Record changes to concomitant medications

Record AEs

Prior to IMRT administration the following procedure will be conducted for all subjects twice during this week, at least two days apart:

Conduct OM assessment and record the severity using the WHO score

Prior to IMRT administration the following observations and procedures will be conducted for all subjects once this week:

Record tobacco use

Measure vital signs, body weight, and ECOG Performance Status

Draw blood for laboratory measurements

- Chemistry profile
- Hematology profile

If receiving cisplatin this week, record BSA (used to confirm cisplatin dosing)

Note: It is recommended that these assessments be completed on the last day of IMRT to avoid having to duplicate them.

Administer GC4419/Placebo doses by continuous intravenous infusion. IMRT must begin as soon as possible but no later than 60 minutes following the end of GC4419 dosing.

10.9.10. Last Day of IMRT or Early Termination Visit

Prior to GC4419/Placebo administration the following observations and procedures will be conducted for all subjects on the last day of IMRT or if the subject terminates study participation early:

Conduct a complete physical examination

Measure vital signs, body weight, and ECOG status

Conduct clinical tumor assessment

Conduct OM assessment and record the severity using the WHO score

Record any changes to concomitant medications

Record AEs

Draw blood for laboratory measurements

- Chemistry profile
- Hematology profile

Note: If safety labs have already been drawn during the study week in which the last day of IMRT or early termination visit falls, then lab safety tests (chemistry and hematology profiles) do not need to be conducted again on the last day of IMRT or at the early termination visit. If safety labs have not been drawn during the current study week at the time of the early termination visit or on the last day of IMRT, then safety labs should be drawn on that day. Safety labs only need to be drawn once per study week after Week 1.

- Cotinine test
- Biomarker sample for pro-inflammatory cytokine analysis (see Section 10.5)
- For subjects who consent separately: RNA sample (PAXgene) for genomic studies (see Section 10.5)

10.10. Follow-up

10.10.1. Post-IMRT OM Follow-up

All subjects will be evaluated at 7 ± 2 calendar days from the last day of IMRT (Post-IMRT Week 1) and at 14 ± 2 calendar days from the last day of IMRT (Post-IMRT Week 2).

At each visit, the following should be completed:

Conduct OM assessment and record the severity using the WHO score

Record AEs and changes to concomitant medications through 30 days following the last dose of IMRT, cisplatin, or GC4419/Placebo (i.e., whichever occurs last).

10.10.2. Long-term Follow-up

All subjects will be followed for one year post-IMRT for tumor recurrence/progression. Subjects will be seen every 3 months (Months 3, 6, 9, and 12 post-IMRT) ± 30 days for each visit. At each of these visits the subject should be seen by a Radiation Oncologist, Medical Oncologist, ENT, or Head & Neck Surgeon and the following assessment should be conducted:

Clinical tumor assessment (See Section 10.6.1)

> Note: a laryngopharyngoscopy is required at Months 3, 6, 9, and 12 post-IMRT. However, if local/regional progression or recurrence has already been discovered at a previous visit, a laryngopharyngoscopy is no longer required at future visits. If distant progression is discovered at a previous visit, a laryngopharyngoscopy is still required at subsequent visits to assess the subject for local/regional progression/recurrence. A head/neck/oral exam must always be conducted at Months 3, 6, 9, and 12 post-IMRT.

Survival status assessment (See Section 10.6.3)

Radiographic imaging must be performed at Month 12 post-IMRT for all subjects. Subjects treated definitively (as opposed to post-operatively) will also undergo imaging at Month 3 post-IMRT to assess tumor response/clearance to the degree possible.

As indicated in Section 10.6.2.2, radiographic imaging is highly recommended if disease progression is suspected by the treating physician at any of the above time points. If radiographic imaging is performed, both local/regional recurrence and distant metastases should be evaluated. Biopsy of any lesion(s) suspicious for tumor recurrence is also recommended.

When possible, the same imaging technique (i.e., CAT, PET, or MRI) that was used for pre-treatment tumor imaging at the time of staging should be used for post-treatment tumor imaging.

The following assessments must also be conducted at 3, 6, 9, and 12 months post-IMRT:

BUN and creatinine assessment

Weight assessment

Subjects will continue to be followed for survival only for two years post-IMRT at 16, 20 and 24 months (see section 10.6.3).

11. ADVERSE EVENTS AND SERIOUS ADVERSE EVENTS

The Investigator is responsible for the detection and documentation of events meeting the criteria and definition of an AE or SAE as provided in this protocol. Throughout the study, AEs will be recorded in the source documents and on the appropriate pages of the CRF regardless of whether the AEs are considered related to GC4419/Placebo. To avoid confusion, the AE should be recorded in standard medical terminology.

11.1. Definitions

The following definitions of terms are guided by the ICH and the US CFR and are included here verbatim.

11.1.1. Adverse Event (AE)

An AE is any untoward medical occurrence in a patient or clinical investigation subject administered a pharmaceutical product and which does not necessarily have a causal relationship with this treatment. An AE can therefore be any unfavorable and unintended sign (including an abnormal laboratory finding), symptom, or disease temporally associated with the use of a medicinal (investigational) product, whether or not related to the medicinal (investigational) product.

Examples of an AE include:

- Significant or unexpected worsening or exacerbation of the condition/indication under study.
- Exacerbation of a chronic or intermittent pre-existing condition including either an increase in frequency and/or intensity (grade) of the condition.
- New conditions detected or diagnosed after investigational product administration even though they may have been present prior to the start of the study.
- Signs, symptoms, or the clinical sequelae associated with a suspected interaction of the investigational product with a concomitant medication.
- Signs, symptoms, or the clinical sequelae associated with a suspected overdose of either investigational product or a concurrent medication.

11.1.2. Serious Adverse Event (SAE)

Any untoward medical occurrence that at any dose:

- Results in death,
- Is life-threatening
  Note: The term ‘life-threatening’ in the definition of ‘serious’ refers to any adverse drug experience [adverse event] that places the patient or subject, in the view of the Investigator, at immediate risk of death from the reaction as it occurred, i.e., it does not include a reaction that, had it occurred in a more severe form, might have caused death. [emphasis added]
- Requires inpatient hospitalization or prolongation of hospitalization
  Note: In general, hospitalization signifies that the patient or subject has been detained (usually involving at least an overnight stay) at the hospital or emergency ward for observation and/or treatment that would not have been appropriate in the physician’s office or outpatient setting. Complications that occur during hospitalization are AEs. If a complication prolongs hospitalization or fulfills any other serious criteria, the event is serious. When in doubt as to whether “hospitalization” occurred or was necessary, the AE should be considered serious. Hospitalization for elective treatment of a pre-existing condition that did not worsen from baseline is not considered an AE.

Results in persistent or significant disability/incapacity

Note: The term disability means a substantial disruption of a person’s ability to conduct normal life functions.

OR

Is a congenital abnormality/birth defect.

11.2. Adverse Event Reporting Requirements

11.2.1. Serious Adverse Events

All events meeting the criteria for Serious Adverse Events (see Section 11.1.2) must be reported by investigational sites within 24-hours of becoming aware of the event. In order to determine the Sponsor’s timeline for notifying regulatory authorities and investigators per Federal Regulations, an event term, serious criteria, and causality is required at the time of the initial report. Specific SAE reporting instructions are provided in a separate manual.

The Investigator is responsible for notifying the IRB/IEC/REB in writing of serious events as soon as is practical in accordance with the policy of the IRB/IEC/REB.

11.2.2. All Adverse Events (AEs) Regardless of Seriousness

Any adverse medical condition or laboratory abnormality with an onset date before the date of randomization is considered to be pre-existing in nature, and part of a subject’s medical history. Adverse medical conditions that begin on or after date of randomization will be considered an adverse event, including SAEs, and followed for 30 days after the last dose of IMRT, cisplatin, or GC4419/Placebo (i.e., whichever occurs last), hereafter referred to as the “30 Day Follow-up Period”. Similarly, new events will be reported as AEs/SAEs if the start date is within 30 Day Follow-up Period. Increases in toxicity grade of pre-existing conditions that occur on or after the date of randomization are also considered an adverse event.

All adverse events must be recorded in the subject’s source documents and on the CRF regardless of frequency, severity (grade) or assessed relationship to randomized therapy.

11.2.3. Clinical Laboratory Abnormalities and Other Abnormal Assessments as AEs and SAEs

Clinically significant abnormal laboratory findings or other abnormal assessments that are associated with the disease being studied, unless judged by the Investigator as more severe than expected for the subject’s condition, or that are present or detected at the time of randomization and do not worsen, will not be reported as AEs or SAEs.

Laboratory abnormalities should only be recorded in the Adverse Event section of the CRF if at least one of the following criteria is met:

Meets the criteria of an SAE

Resulted in a dose reduction and/or delay in the administration of GC4419/Placebo, IMRT, and/or cisplatin

Treatment is initiated for the abnormality

Investigational product was discontinued

Grade 3 or Grade 4 per NCI CTCAE v5.0

All other abnormal laboratory findings will be captured via laboratory CRF pages and noted in shift tables.

Abnormal assessments (e.g., ECGs) that are judged by the Investigator as clinically significant will be recorded as AEs or SAEs if they meet the definitions as defined in Section 11.1.

11.2.4. Disease-Related Events and/or Disease-Related Outcomes Not Qualifying as AEs or SAEs

Oral mucositis will not be reported as an AE as it is captured as a study endpoint in the CRF. Progressive disease found by scan or on clinical evaluation should be captured on the applicable CRF pages and not as an AE.

11.2.5. Grading of Adverse Events

The severity of adverse events will be designated as mild, moderate, severe, life threatening, or fatal per NCI CTCAE version 5.0. If not specifically addressed in NCI CTCAE version 5.0, use Table 5 below:

Table 5: Adverse Event Severity

Grade	Criteria[1]
Mild – Grade 1	Asymptomatic or mild symptoms; clinical or diagnostic observations only; intervention not indicated
Moderate – Grade 2	Minimal, local, or noninvasive intervention indicated; limiting age-appropriate instrumental ADL[2]
Severe – Grade 3	Severe or medically significant but not immediately life-threatening; hospitalization or prolongation of hospitalization indicated; disabling; limiting self-care ADL[3]
Life Threatening – Grade 4	Life-threatening consequences; urgent intervention indicated
Death – Grade 5	Death related to adverse event

1 A semi-colon indicates 'or' within the description of the grade.
2 Instrumental ADL refer to preparing meals, shopping for groceries or clothes, using the telephone, managing money, etc.
3 Self-care ADL refer to bathing, dressing and undressing, feeding self, using the toilet, taking medications, and not bedridden.

11.3. Relationship to Study Drug

All AEs will be categorized by the Investigator with respect to their relationship to GC4419/Placebo. The Investigator should consult the IB and/or product information in the determination of his/her assessment. The relationship between GC4419/Placebo and the AE may

be considered related, possibly related, or unrelated. The criteria for each category are listed below:

Related: It is likely that GC4419/Placebo caused or contributed to the cause of the adverse event or laboratory abnormality, when the temporal sequence from the time of GC4419/Placebo administration, the known consequences of the subject's clinical/state condition or study procedures, the effects of discontinuing or reintroducing GC4419/Placebo on the adverse event, and other medically relevant factors are considered.

Possibly Related: There is a reasonable possibility that the adverse event or laboratory abnormality was caused by GC4419/Placebo, when the temporal sequence from the time of GC4419/Placebo administration, the known consequences of the subject's clinical state/condition or study procedures, and other medically relevant factors are considered.

Unrelated: The Investigator has a high level of certainty that the subject's clinical state/condition, study procedures, or other medically relevant factors other than treatment with GC4419/Placebo caused the adverse event or laboratory abnormality. This relationship category should only be used when a clear precipitating cause exists and it is not reasonably possible that the event is caused by treatment with GC4419/Placebo.

If the relationship between the AE/SAE and the investigational product is determined to be "possibly related" the event will be considered to be related to the investigational product for the purposes of expedited regulatory reporting.

11.4. Recording Adverse Events

All AEs must be recorded on the appropriate CRF regardless of the severity or relationship to GC4419/Placebo. All AEs that meet the seriousness criteria should also be recorded on the SAE Report Form. All SAEs must be reported to the Sponsor or delegated organization within the timeline stated in Section 11.2.

The recording of AEs will be based on data obtained from the following sources:

- Medical and surgical history
- Physical examinations including vital signs
- Clinical laboratory test results
- Subject verbal reports to the investigational staff and documented in the medical chart

All clinical events, including both observed (such as any reaction at sites of application) and volunteered problems, complaints, or symptoms, are to be recorded. The need to capture this information is not dependent upon whether the clinical event is associated with GC4419/Placebo use. AEs resulting from concurrent illnesses, reactions to concurrent medications or symptomatic progression of disease states are also to be recorded.

The information to be recorded for AEs will include:

- The specific type of event in standard medical terminology – diagnosis if known, is preferred over symptoms
- Duration of the clinical event (start and stop dates)

Severity (Grade 1, 2, 3, 4, or 5) of the clinical event

Seriousness (SAE) criteria, if applicable

Relationship of the AE to GC4419/Placebo as defined in Section 11.3

Management of GC4419/Placebo administration and other action taken to alleviate the clinical events

Clinical outcome of the AE

11.5. Follow-up of AEs and SAEs

If an adverse event changes in grade within the date of randomization through the 30 Day Reporting Period, the event should be recorded as a new AE.

After the initial AE/SAE report, the Investigator is required to proactively follow each subject and provide further information on the subject's condition. The Investigator will ensure that follow-up includes any supplemental investigations as may be indicated to elucidate the nature and/or causality of the AE or SAE. This may include additional laboratory tests or investigations, histopathological examinations, or consultation with other health care professionals.

Non-serious AEs that have not resolved within 30 days after last dose of GC4419/Placebo will be considered ongoing, and marked as such in the CRF. All SAEs will be followed until they resolve or a new baseline is established, at which point the appropriate CRF page(s) or SAE Report Form(s) will be updated.

Routine collection of AEs will stop 30 days after last dose of GC4419/Placebo, IMRT, or cisplatin (i.e., whichever occurs last); however, collection of clinical data will continue on AEs of interest or as clinical circumstances warrant that exceed the 30 days after last dose of GC4419/Placebo, per below and described in other sections of the Protocol:

- OM: 2 weeks post last IMRT dose (Section 10.10.1)
- Medical events, which in the opinion of the Investigator, serious and are believed to be a result of study participation to warrant notifying the Sponsor (Sections 11.6 and 11.8). In these circumstances, the Investigator should contact the Galera Medical Monitor (or designee) directly to discuss the case, and how it should be reported.

As reasonably requested by the Sponsor, the Investigator will perform or arrange for the conduct of supplemental measurements and/or evaluations to elucidate as fully as possible the nature and/or causality of the AE or SAE. If a subject dies during participation in the study or during a recognized follow-up period, the Sponsor will be provided with a copy of any post-mortem findings, including histopathology.

11.6. Post-Study Reporting Requirements

Although such information may not be routinely sought or collected by the Sponsor, SAEs that occur after the subject has completed a clinical study may be reported. Such cases will be evaluated for expedited reporting.

11.7. Regulatory Reporting of Adverse Events

The Sponsor will have final determination of when an event must be reported to the relevant regulatory authorities and is responsible for notifying the relevant regulatory authorities of certain events. The Investigator will report all SAEs that occur at his/her site to the IRB per the site's IRB regulations. AEs will be reported to regulatory authorities in compliance with 21 CFR 312.32, local and regional law and established guidance by the Sponsor or its designee. The format of the reports will be dictated by the local and regional requirements.

Investigators will also be notified of all unexpected, serious, drug-related events (7/15-Day Safety Reports) that occur during the clinical study. Each site is responsible for notifying its IRB/IEC/REB of these additional SAEs in accordance with local or central IRB/IEC/REB procedures. Copies of each report will be kept in the Investigator's files and adequate documentation will be provided to the Sponsor including documentation that the IRB/IEC/REB was notified of each safety report.

11.8. Pregnancy

The risks of treatment with GC4419 during pregnancy have not been evaluated. Male subjects and female subjects of childbearing potential who engage in sexual intercourse should use a barrier method of contraception throughout the study and for 30 days (females) or 90 days (males) following the last dose of GC4419/Placebo.

11.8.1. Time Period for Collecting Pregnancy Information

As permitted by IRB/EC/REB policies, any pregnancy that occurs from the first dose of GC4419/Placebo up to 30 days after last dose should be reported using the appropriate form within 2 weeks of learning of the subject's pregnancy. The subject will be followed throughout the course of the pregnancy. Generally, follow-up will be no longer than 6 to 8 weeks following the estimated delivery date. Any premature termination of the pregnancy should be reported. If a pregnancy is identified outside the 30 days after last dose, the Investigator may report using clinical judgment.

11.8.2. Action to be Taken if Pregnancy Occurs in a Female Partner of a Male Subject

The Investigator will attempt to collect pregnancy information on any female partner of a male study subject who becomes pregnant while the male partner is participating in this study. The Investigator will record pregnancy information on the appropriate form and submit it to the Sponsor within 2 weeks of learning of the partner's pregnancy. The partner will also be followed to determine the outcome of the pregnancy. Information on the status of the mother and child will be forwarded to the Sponsor. Generally, follow-up will be no longer than 6 to 8 weeks following the estimated delivery date. Any premature termination of the pregnancy will be reported. If a pregnancy is identified outside the 30 days after last dose, the Investigator may report using clinical judgment.

12. STATISTICS

12.1. General Considerations

A separate Statistical Analysis Plan will provide technical details of the statistical analyses to be performed, in addition to the specifications in this protocol. In the event of discrepancies between the protocol and the Statistical Analysis Plan, the latter will control the analyses performed.

All statistical analyses will be conducted with the SAS® software package version 9.2 or higher.

12.1.1. Randomization and Stratification

Eligible subjects will be randomized 3:2 to the two treatment arms. Randomization will be stratified by:

Assigned cisplatin schedule (q3 weeks or weekly).

Post-operative vs. definitive treatment

12.1.2. Treatment of Subjects Affected by Sponsor Dosing Suspension

On April 28, 2019, the Sponsor voluntarily suspended dosing in the trial following observation of fine particulate matter in two lots of clinical Drug Product (DP) at the 9-month stability test point. Subsequent analysis found that these two DP lots remained within specifications, and the adverse event profile in subjects who had received GC4419 from them was not different from observations in subjects who had received GC4419 from unaffected DP lots. Particulates had neither been observed in the DP lots in question at prior test points (3 and 6 months), nor in prior DP lots. The FDA's Division of Dermatology and Dental Products subsequently placed the IND on full clinical hold on July 15, 2019. The Sponsor submitted a Complete Response to Clinical Hold on August 7, 2019, and FDA removed the clinical hold on August 30, 2019. No subjects were enrolled to the study or received study drug from April 28, 2019 until after the removal of the clinical hold.

The "intent to treat" (ITT) population is defined to consist of all randomized subjects who receive at least one dose of blinded GC4419 or placebo. However, when the Sponsor suspended dosing, 28 subjects were in the course of receiving GC4419 or placebo. These 28 subjects will be excluded from the ITT analysis on the ground that their participation does not reflect an adequate test of the hypothesized effects of the experimental agent.

12.2. Sample Size

Approximately 450 total subjects (270 GC4419:180 Placebo) will be enrolled from investigational sites in the United States and Canada to obtain approximately 400 ITT subjects (240 GC4419:160 Placebo). The proposed ITT sample size of 400 assumes that 10% will discontinue early, yielding approximately 216 subjects in the experimental arm and approximately 144 in the Placebo arm who will complete their IMRT course.

With 144 subjects in the control arm and 216 subjects in the experimental arm, the study will have approximately 95% power to detect a relative reduction in incidence of severe OM throughout the treatment period in the experimental arm of approximately 34% compared to Placebo (i.e., 43% incidence for GC4419 vs 65% for Placebo).

12.3. Analysis Populations

Safety and efficacy analyses will be conducted on the ITT population, consisting of all randomized subjects who receive at least one dose of GC4419/Placebo, excluding the 28 subjects whose dosing was affected by the Sponsor's dosing suspension decision. Subjects will be analyzed according to their randomized treatment assignments.

Randomized subjects who withdraw prior to any dosing will have their reasons for withdrawal noted, and their treatment assignment will remain blinded until the end of the study.

Analyses on other efficacy populations (e.g., "per protocol" population) may be conducted. Such analyses will be specified in the Statistical Analysis Plan for this study.

12.4. Safety Analysis

AEs will be grouped by system organ class, high level term, and preferred term according to the Medical Dictionary for Regulatory Activities (MedDRA) dictionary. Incidence by subject will be tabulated for all treatment-emergent, serious, severe, and treatment-related adverse events. Detailed listings will be provided for all serious adverse events, deaths, and withdrawals due to adverse events.

Laboratory measurements and vital signs will be summarized by treatment group at each of the protocol-specified time points.

Safety parameters will be summarized with descriptive statistics on all subjects who received at least one dose of GC4419/Placebo. Safety analyses will be conducted on the ITT population and the dosing suspension subjects separately.

Further details will be provided in the study's Statistical Analysis Plan.

12.5. Primary Efficacy Analysis

SOM incidence (i.e., the proportion of subjects with SOM) during the Study Treatment Period will be compared between the GC4419 and Placebo arms by the Cochran-Mantel-Haenszel (CMH) test at a 2-sided Type 1 error rate of 0.05.

Efficacy analyses will be stratified by the factors used in randomization, namely post-operative vs. definitive treatment and planned chemotherapy schedule.

Details of the primary efficacy analysis will be included in the Statistical Analysis Plan.

12.5.1. Multiplicity

To control the overall Type 1 error at 0.05, the primary endpoint will first be tested at a two-sided 0.05 level. If the primary endpoint result is significant at this level, then Holm-Bonferroni multiplicity testing will be conducted on the following proposed secondary endpoints applying a familywise two-sided alpha of 0.05:

- incidence of Grade 4 OM during the Study Treatment Period;
- total number of SOM days (per subject) during the OM Observation Period for the entire study population; and

- total number of days of Grade 4 OM (per subject) during the OM Observation Period for the entire study population.

Further details regarding multiplicity testing will be included in the Statistical Analysis Plan.

12.5.2. Handling of Missing Data

Subjects who withdraw consent for GC4419/Placebo administration should be encouraged to continue and complete standard IMRT/cisplatin, and protocol assessments, notably scheduled OM assessments.

Investigators should make reasonable attempts to continue to collect WHO OM scores for inclusion in the study database for subjects who discontinue from GC4419/Placebo, cisplatin, or IMRT.

The study's Statistical Analysis Plan will discuss the imputation strategy for subjects who lack WHO OM scores, who discontinue from the study without complete follow-up of WHO OM scores, and whose resolution date of severe OM is unknown.

12.5.3. Covariate Adjustment

Because the randomization is stratified by baseline post-operative vs definitive treatment status, and by planned cisplatin schedule, the primary analysis will be stratified by these factors. The Statistical Analysis Plan will contain guidelines for combining strata should the size of any of the four strata be unacceptably small.

12.6. Secondary Efficacy Analyses

12.6.1. Secondary Endpoints

Secondary endpoints will be analyzed as described in Section 12.5.1; testing will be stratified by the factors used in randomization.

The number of days of SOM, or of Grade 4 OM, will be determined as follows:

- Subjects who never develop SOM during the OM Observation Period will be judged to have zero days of SOM
- Subjects who never develop Grade 4 OM during the OM Observation Period will be judged to have zero days of Grade 4 OM
- For subjects who do develop SOM or Grade 4 OM, the number of days of SOM or Grade 4 OM will be taken to include all days from the first observation of SOM or Grade 4 OM to the last observation of SOM or Grade 4 OM, with no subsequent SOM or Grade 4 OM

Additional details will be described in the Statistical Analysis Plan.

12.7. Exploratory Efficacy Analyses

Exploratory efficacy analyses will be described in the Statistical Analysis Plan.

12.8. Analysis of Tumor Endpoints

12.8.1. Local or Regional Progression

Local (primary site) or regional (neck) progression is defined as clinical or radiographic evidence of progressive disease at the primary site or neck. The location of progressive disease should be separately distinguished (local vs. neck) if possible. Progression of local or regional disease should be confirmed by biopsy when possible but may be clinically assessed and documented in the clinical record at the judgment of the treating clinicians. Tumor reappearing with the initial and immediate adjoining anatomical region of the primary will be considered local recurrence.

12.8.2. Distant Metastasis

Clear evidence of distant metastases (lung, bone, brain, etc.): biopsy is recommended where possible. Multiple lung nodules/masses are considered distant metastases from the index cancer unless proven otherwise.

The frequencies of locoregional and distant failure will be tabulated and standard Kaplan-Meier plots of cumulative event rates will be made for time-to-event endpoints (OS, PFS, locoregional failure over time, distant metastases over time).

12.8.3. Second Primary Neoplasm

A solitary, speculated lung mass/nodule is considered a second primary neoplasm unless proven otherwise.

Frequency of second primary neoplasms will be tabulated.

12.9. Interim Analysis

No interim efficacy analysis is planned.

An independent Data Monitoring Committee (DMC) will perform periodic unblinded safety reviews while subjects in the study receive GC4419/Placebo.

13. ETHICS

13.1. Ethical Conduct of the Study

The Investigator will ensure that this study is conducted in full compliance with the principles of the "Declaration of Helsinki" (version October 2008), ICH guidelines, in particular ICH-GCP, or with the laws and regulations of the country in which the research is conducted, whichever affords the greatest protection to the study subject. The Investigator will also assure that the basic principles outlined in "ICH Guideline for Good Clinical Practice" as published in the Federal Register May 9, 1997, and all applicable Federal regulations including 21 CFR parts 50, 54, 56 and 312 are adhered to.

13.2. Institutional Review Board (IRB)/Independent Ethics Committee (IEC)/Research Ethics Board (REB) Approval

This protocol and any accompanying material to be provided to the subject (such as advertisements, information sheets, or descriptions of the study used to obtain informed consent)

will be submitted, by the Investigator, to an IRB/IEC/REB. Approval from the IRB/IEC/REB must be obtained, and a copy must be provided to the Sponsor or its representatives before initiating the conduct of any study procedures including screening or enrolling any patients into the study.

No modifications or deviations from this protocol other than those that are deemed medically necessary by the Principal Investigator or designated sub-Investigator are to be made without prior, written approval by the Sponsor. Significant protocol deviations will be reported to the Sponsor and to the IRB/IEC/REB in accordance with its reporting policy.

Any modifications made to the protocol by the Sponsor after receipt of IRB/IEC/REB approval must be submitted to the committee for approval prior to implementation.

13.3. Written Informed Consent

In accordance with regulatory and local IRB/IEC/REB requirements, before study procedures are performed, patients will be informed about the study and required to sign the IRB/IEC/REB approved Informed Consent Form (ICF). This form will be signed after adequate explanation of the aims, methods, objective and potential hazards of the study and prior to undertaking any study-related procedures. The Sponsor or its designee will provide an ICF template to the Investigator. The Sponsor or its designee must approve changes to the ICF template prior to submission to the IRB/IEC/REB. Informed consent will be obtained according to the applicable IRB/IEC/REB requirements. No patient is to be screened or treated until an ICF, written in a language in which the patient is fluent, has been obtained. The signed ICF will be retained with the study records. Each patient will also be given a copy of his/her signed ICF.

13.4. Data Monitoring Committee (DMC)

An independent Data Monitoring Committee (DMC) will perform periodic unblinded safety reviews while subjects in the study receive GC4419/Placebo. The DMC will make recommendations regarding the conduct of the study, i.e., to continue enrollment, to hold enrollment until further review, to amend the protocol, or to stop the study early.

The details of the DMC meetings and responsibilities are outlined in a separate DMC Charter.

14. DIRECT ACCESS TO SOURCE DATA/DOCUMENTS

14.1. Study Monitoring

In accordance with ICH-GCP guidelines, the study monitor must have direct access to the Investigator’s source documentation in order to verify the consistency of the data recorded in the electronic CRFs.

The monitor is responsible for routine review of the electronic CRFs at regular intervals throughout the study, to verify adherence to the protocol, and the completeness, consistency and accuracy of the data being entered on them. The monitor should have full access to any subject records needed to verify the entries on the electronic CRFs. The Investigator agrees to cooperate with the monitor to assure that any follow-up items identified in the course of these monitoring visits are resolved.

During site visits, the monitor will:

- Check the progress of the study;
- Review study data collected;
- Conduct source document verification;
- Identify any issues and address their resolution.

This will be done in order to verify that the:

- Data are authentic, accurate, and complete;
- Safety and rights of subjects are being protected;
- Study is conducted in accordance with the currently approved protocol (and any amendments), GCP, and all applicable regulatory requirements.

The Investigator agrees to allow the monitor direct access to all relevant documents and to allocate his/her time and the time of his/her staff to the monitor to discuss findings and any relevant issues.

14.2. Audits and Inspections

Authorized representatives of the Sponsor, a regulatory authority, an Independent Ethics Committee or an Institutional Review Board may visit the site to perform audits or inspections, including source data verification. The purpose of a Sponsor audit or inspection is to systematically and independently examine all study-related activities and documents to determine whether these activities were conducted, and data were recorded, analyzed, and accurately reported according to the protocol, GCP guidelines of the ICH, and any applicable regulatory requirements. The Investigator should contact the Sponsor immediately if contacted by a regulatory agency about an inspection.

14.3. Protocol Compliance

The Investigator is responsible for ensuring the study is conducted in accordance with the procedures and evaluations described in this protocol.

14.4. Protocol Modifications

Protocol modifications, except those intended to reduce immediate risk to study subjects, may be made only by the Sponsor or its representatives. All protocol modifications must be submitted to the IRB/IEC/REB in accordance with local requirements. Approval must be obtained before changes can be implemented.

14.5. Information Disclosure

14.5.1. Ownership

All information provided by the Sponsor or its representatives, and all data and information generated by the site as part of the study (other than a subject’s medical records), are the sole property of the Sponsor.

14.5.2. Confidentiality

All information provided by the Sponsor or its representatives, and all data and information generated by the site as part of the study (other than a subject's medical records) will be kept confidential by the Investigator and other site staff. Information related to this study is subject to the confidentiality provisions of the Clinical Research Agreement between the investigative site and the Sponsor.

14.5.3. Publication

All publication or presentation rights for the findings of the clinical investigation under this protocol shall be governed by the appropriate terms of the Clinical Research Agreement between the investigational site and the Sponsor.

15. QUALITY CONTROL AND QUALITY ASSURANCE

The study will be monitored and managed in accordance with ICH-GCP.

To ensure compliance with GCP and all applicable regulatory requirements, the Sponsor or its representatives may conduct a quality assurance audit. Regulatory agencies may also conduct a regulatory inspection of this study. Such audits/inspections can occur at any time during or after completion of the study. If an audit or inspection occurs, the Investigator and institution agree to allow the auditor/inspector direct access to all relevant documents and to allocate his/her time and the time of his/her staff to the auditor/inspector to discuss findings and any relevant issues.

16. DATA HANDLING AND RECORDKEEPING

16.1. Case Report Forms

All required study data must be recorded on the electronic CRF provided by the Sponsor or its representatives. The data recorded onto the electronic CRF is derived from the source documents. The Investigator shall ensure that all data in the electronic CRF is accurate and consistent with the source documents or that any discrepancies of the electronic CRF with source documents are explained (ICH-GCP 4.9.2).

Electronic case report forms will be accessed by the study center for collection of all study data, and a copy of the electronic CRF will be provided to the site for the Investigator files. For each subject who is randomized, the electronic CRF must be completed by site staff and must be signed electronically by the Principal Investigator in a timely fashion after data collection. If a subject withdraws from the study, the electronic CRFs should be promptly completed and the reason for withdrawal must be noted. If a subject is withdrawn from the study because of a drug-related toxicity, thorough efforts should be made to clearly document the outcome.

16.2. Retention/Inspection of Records

The Investigator must maintain adequate and accurate records to enable the conduct of the study to be fully documented and the study data to be subsequently verified.

Records of drug receipt and disposition, electronic file of CRFs, source documents, reports of this investigation and other study documentation must be maintained by the Investigator for a period of at least two years for US sites and 25 years for Canadian sites following the date on

which the investigational drug is approved by FDA or other applicable regulatory agency for marketing for the purposes that were the subject of the clinical investigations. If no application is to be filed, records must be retained until two years for US sites and 25 years for Canadian sites following the date that the study is discontinued, and the FDA or other applicable regulatory agency is notified. If the application is not approved by the FDA or other applicable regulatory agency for such indication, records must be retained for two years for US sites and 25 years for Canadian sites after notification by the Sponsor of the FDA or other applicable regulatory agency decision. The records must be available for copying and inspection if requested by regulatory authorities.

The Sponsor should be notified in writing at least 30 days prior to the disposal or transfer to another location or party of any study records related to this protocol.

17. LIST OF REFERENCES

Anderson CM, Sonis ST, Lee CM, Adkins D, Allen BG, Sun W, et al. (2018). "Phase 1b/2a Trial of the Superoxide Dismutase Mimetic GC4419 to Reduce Chemoradiotherapy-Induced Oral Mucositis in Patients With Oral Cavity or Oropharyngeal Carcinoma." International journal of radiation oncology, biology, physics **100**(2): 427-435.

Ang, K. K., Q. Zhang, et al. (2014). "Randomized phase III trial of concurrent accelerated radiation plus cisplatin with or without cetuximab for stage III to IV head and neck carcinoma: RTOG 0522." Journal of clinical oncology : official journal of the American Society of Clinical Oncology **32**(27): 2940-2950.

Bodey, G. P., V. Rodriguez, et al. (1978). "Fever and infection in leukemic patients: a study of 494 consecutive patients." Cancer **41**(4): 1610-1622.

Bonner, J. A., P. M. Harari, et al. (2006). "Radiotherapy plus cetuximab for squamous-cell carcinoma of the head and neck." The New England journal of medicine **354**(6): 567-578.

Buettner, G. R., C. F. Ng, et al. (2006). "A new paradigm: manganese superoxide dismutase influences the production of H2O2 in cells and thereby their biological state." Free radical biology & medicine **41**(8): 1338-1350.

Delanian, S., F. Baillet, et al. (1994). "Successful treatment of radiation-induced fibrosis using liposomal Cu/Zn superoxide dismutase: clinical trial." Radiotherapy and oncology : journal of the European Society for Therapeutic Radiology and Oncology **32**(1): 12-20.

Elting, L. S., C. D. Cooksley, et al. (2007). "Risk, outcomes, and costs of radiation-induced oral mucositis among patients with head-and-neck malignancies." International journal of radiation oncology, biology, physics **68**(4): 1110-1120.

Elting, L. S., D. M. Keefe, et al. (2008). "Patient-reported measurements of oral mucositis in head and neck cancer patients treated with radiotherapy with or without chemotherapy: demonstration of increased frequency, severity, resistance to palliation, and impact on quality of life." Cancer **113**(10): 2704-2713.

Epperly, M. W., S. Defilippi, et al. (2000). "Intratracheal injection of manganese superoxide dismutase (MnSOD) plasmid/liposomes protects normal lung but not orthotopic tumors from irradiation." Gene therapy **7**(12): 1011-1018.

Fakhry C, Zhang Q, Nguyen-Tan PF, et.al. (2014). "Human Papillomavirus and Overall Survival After Progression of Oropharyngeal Squamous Cell Carcinoma." Journal of clinical oncology : official journal of the American Society of Clinical Oncology **32**:3365-3373.

Galera clinical study report number U20-03-06-001 A Double-Blind, Placebo-Controlled, Single Rising Dose Study to Evaluate the Safety and Tolerability and Determine the Pharmacokinetics of M40419 Administered as a 15 Minute Intravenous Infusion in Healthy Subjects.

Gillison et al. (2018). "Radiotherapy plus cetuximab or cisplatin in human papillomavirus-positive oropharyngeal cancer (NRG Oncology RTOG 1016): a randomised, multicentre, non-inferiority trial." Lancet. 393: 40-50.

Guo, H., J. A. Seixas-Silva, Jr., et al. (2003). "Prevention of radiation-induced oral cavity mucositis by plasmid/liposome delivery of the human manganese superoxide dismutase (SOD2) transgene." Radiation research **159**(3): 361-370.

Hahn, T., E. Zhelnova, et al. (2010). "A deletion polymorphism in glutathione-S-transferase mu (GSTM1) and/or theta (GSTT1) is associated with an increased risk of toxicity after autologous blood and marrow transplantation." Biology of blood and marrow transplantation : journal of the American Society for Blood and Marrow Transplantation **16**(6): 801-808.

Henke, M., M. Alfonsi, et al. (2011). "Palifermin decreases severe oral mucositis of patients undergoing postoperative radiochemotherapy for head and neck cancer: a randomized, placebo-controlled trial." Journal of clinical oncology : official journal of the American Society of Clinical Oncology **29**(20): 2815-2820.

Hesketh PJ, Kris MG, Basch E, et al. (2017). "Antiemetics: American Society of Clinical Oncology Clinical Practice Guideline Update." Journal of Clinical Oncology **35**:3240-3261.

Jacinto JCK, Co J, et.al. (2017). "The evidence on effectiveness of weekly vs triweekly cisplatin concurrent with radiotherapy in locally advanced head and neck squamous cell carcinoma (HNSCC): a systematic review and meta-analysis." British Journal of Radiology **90**:(1079).

Keefe, D. M. (2006). "Mucositis guidelines: what have they achieved, and where to from here?" Supportive care in cancer : official journal of the Multinational Association of Supportive Care in Cancer **14**(6): 489-491.

Kudrimoti, M., A. Curtis, et al. (2016). "Dusquetide: A novel innate defense regulator demonstrating a significant and consistent reduction in the duration of oral mucositis in preclinical data and a randomized, placebo-controlled phase 2a clinical study." Journal of biotechnology **239**: 115-125.

Lalla, R. V., J. Bowen, et al. (2014). "MASCC/ISOO clinical practice guidelines for the management of mucositis secondary to cancer therapy." Cancer **120**(10): 1453-1461.

Lalla, R. V. and D. E. Peterson (2006). "Treatment of mucositis, including new medications." Cancer journal **12**(5): 348-354.

Le, Q. T., H. E. Kim, et al. (2011). "Palifermin reduces severe mucositis in definitive chemoradiotherapy of locally advanced head and neck cancer: a randomized, placebo-controlled study." Journal of clinical oncology : official journal of the American Society of Clinical Oncology **29**(20): 2808-2814.

McCord, J. M. and I. Fridovich (1969). "Superoxide dismutase. An enzymic function for erythrocuprein (hemocuprein)." The Journal of biological chemistry **244**(22): 6049-6055.

Mendenhall, W. M., R. J. Amdur, et al. (2006). "Intensity-modulated radiotherapy in the standard management of head and neck cancer: promises and pitfalls." Journal of clinical oncology : official journal of the American Society of Clinical Oncology **24**(17): 2618-2623.

MuGard prescribing information.

Murphy, C. K., E. G. Fey, et al. (2008). "Efficacy of superoxide dismutase mimetic M40403 in attenuating radiation-induced oral mucositis in hamsters." Clinical cancer research : an official journal of the American Association for Cancer Research **14**(13): 4292-4297.

Nguyen-Tan, P. F., Q. Zhang, et al. (2014). "Randomized phase III trial to test accelerated versus standard fractionation in combination with concurrent cisplatin for head and neck carcinomas in the Radiation Therapy Oncology Group 0129 trial: long-term report of efficacy and toxicity." Journal of clinical oncology : official journal of the American Society of Clinical Oncology **32**(34): 3858-3866.

Nonzee, N. J., N. A. Dandade, et al. (2008). "Evaluating the supportive care costs of severe radiochemotherapy-induced mucositis and pharyngitis : results from a Northwestern University Costs of Cancer Program pilot study with head and neck and nonsmall cell lung cancer patients who received care at a county hospital, a Veterans Administration hospital, or a comprehensive cancer care center." Cancer **113**(6): 1446-1452.

Noronha v, Joshi A, et al. (2018). "Once-a-Week Versus Once-Every-3-Weeks Cisplatin Chemoradiation for Locally Advanced Head and Neck Cancer: A Phase III Randomized Noninferiority Trial." Journal of clinical oncology : official journal of the American Society of Clinical Oncology **36**:1064-1072.

Overgaard, J., H. S. Hansen, et al. (2003). "Five compared with six fractions per week of conventional radiotherapy of squamous-cell carcinoma of head and neck: DAHANCA 6 and 7 randomised controlled trial." Lancet **362**(9388): 933-940.

Pignon, J. P., A. le Maitre, et al. (2009). "Meta-analysis of chemotherapy in head and neck cancer (MACH-NC): an update on 93 randomised trials and 17,346 patients." Radiotherapy and oncology : Journal of the European Society for Therapeutic Radiology and Oncology **92**(1): 4-14.

Rao, N. G., A. Trotti, et al. (2014). "Phase II multicenter trial of Caphosol for the reduction of mucositis in patients receiving radiation therapy for head and neck cancer." Oral oncology **50**(8): 765-769.

Riley, P. A. (1994). "Free radicals in biology: oxidative stress and the effects of ionizing radiation." International journal of radiation biology **65**(1): 27-33.

Ruescher, T. J., A. Sodeifi, et al. (1998). "The impact of mucositis on alpha-hemolytic streptococcal infection in patients undergoing autologous bone marrow transplantation for hematologic malignancies." Cancer **82**(11): 2275-2281.

Russo, G., R. Haddad, et al. (2008). "Radiation treatment breaks and ulcerative mucositis in head and neck cancer." The oncologist **13**(8): 886-898.

Salama, J. K., T. Y. Seiwert, et al. (2007). "Chemoradiotherapy for locally advanced head and neck cancer." Journal of clinical oncology : official journal of the American Society of Clinical Oncology **25**(26): 4118-4126.

Sonis, S. and Galera Report no 1009-4419-0311 A Comparison of GC4419 and GC4403 for the Prevention of Oral Mucositis Induced by Acute Radiation in Hamsters. Biomodels Report Gal-03.

Sonis, S. and Galera Report no 1010-4419-0311 An Evaluation of GC4419 for the Prevention of Oral Mucositis Induced by Fractionated Radiation in Hamsters. Biomodels Report Gal-04.

Sonis, S., R. Haddad, et al. (2007). "Gene expression changes in peripheral blood cells provide insight into the biological mechanisms associated with regimen-related toxicities in patients being treated for head and neck cancers." Oral oncology **43**(3): 289-300.

Sonis, S. T. (2004). "The pathobiology of mucositis." Nature reviews. Cancer **4**(4): 277-284.

Sonis, S. T. (2009). "Mucositis: The impact, biology and therapeutic opportunities of oral mucositis." Oral oncology **45**(12): 1015-1020.

Sonis, S. T. (2011). "Oral mucositis." Anti-cancer drugs **22**(7): 607-612.

Sonis, S. T., L. S. Elting, et al. (2004). "Perspectives on cancer therapy-induced mucosal injury: pathogenesis, measurement, epidemiology, and consequences for patients." Cancer **100**(9 Suppl): 1995-2025.

Spielberger, R., P. Stiff, et al. (2004). "Palifermin for oral mucositis after intensive therapy for hematologic cancers." The New England journal of medicine **351**(25): 2590-2598.

Stojan P, Vermorken JB, et al. (2016). "Cumulative cisplatin dose in concurrent chemoradiotherapy for head and neck cancer: A systematic review." Head Neck **38**:E2151-E2158.

Sturtz P, Wouters K, et al. (2017). "Weekly Low-Dose Versus Three-Weekly High-Dose Cisplatin for Concurrent Chemoradiation in Locoregionally Advanced NonNasopharyngeal Head and Neck Cancer: A Systematic Review and Meta-Analysis of Aggregate Data." The Oncologist **22**:1056–1066.

Thompson, J. S., Y. Chu, et al. (2010). "The manganese superoxide dismutase mimetic, M40403, protects adult mice from lethal total body irradiation." Free radical research **44**(5): 529-540.

Traynor, A. M., G. M. Richards, et al. (2010). "Comprehensive IMRT plus weekly cisplatin for advanced head and neck cancer: the University of Wisconsin experience." Head & neck **32**(5): 599-606.

Trotti, A., Harris, J., et al. (2018). "NRG-RTOG 1016: Phase III Trial Comparing Radiation/Cetuximab to Radiation/Cisplatin in HPV-related Cancer of the Oropharynx." International Journal of Radiation Oncology, Biology, Physics Supplement, 2018.

Uno H, Claggett B, Tian L, et al. (2014). "Moving Beyond the Hazard Ratio in Quantifying the Between-Group Difference in Survival Analysis." Journal of clinical oncology : official journal of the American Society of Clinical Oncology **32**:2380-2385.

Vera-Llonch, M., G. Oster, et al. (2006). "Oral mucositis in patients undergoing radiation treatment for head and neck carcinoma." Cancer **106**(2): 329-336.

18. APPENDICES

APPENDIX 1. SCHEDULE OF ASSESSMENTS

Table 6: Schedule of Assessments

Assessments	Screening	Treatment									Follow-up			
											Post-IMRT OM Follow-up	Long-term Follow-up		
	≤28 days of IMRT Day 1	Wk 1		Wk 2	Wk 3	Wk 4	Wk 5	Wk 6	Wk 7	Last Day of IMRT or Early Term[1]	Post-IMRT Wks 1 & 2 (±2 D)[2]	Months 3, 6, 9, & 12 Post-IMRT (±30 D)	Months 3 & 12 Post-IMRT (±30 D)	Months 16, 20, 24 Post-IMRT (±30 D)
		Baseline (IMRT Day 1)	Days 2-7	Days 8-14	Days 15-21	Days 22-28	Days 29-35	Days 36-42	Days 43-49					
Informed consent	X													
Inclusion/exclusion criteria[3]	X	X												
Dental exam/IMRT clearance	X													
Medical and HNC histories[4]	X													
Record tobacco use	X	X		X	X	X	X	X	X					
Complete physical exam[5]	X									X				
Height	X													
Weight	X	X		X	X	X	X	X	X	X				
Symptom-directed PE						X								
Vital signs, ECOG[6]	X	X				X			X	X				
Record BSA[7]		X		X	X	X	X	X	X					
Tumor imaging[8]	X												X	
Clinical tumor assessment[9]										X		X	X	
Concomitant medications[10]	X	X	Weekdays							X	X			
Adverse Events[11]	X	X	Weekdays							X	X			
ECG (12-lead)[12]	X													
OM assessment[13]	X	X	X	Twice-weekly					X	X	X			
Blood draw: Serum pregnancy test[14]	X													
Blood draw: Lab safety tests[15]	X	X	X	X	X	X	X	X	X	X[16]				
Blood draw: Serum cotinine[17]		X				X				X				
Blood draw: PK[18]		X	X			X								
Blood draw: Cytokines[19]	X	X		X		X		X		X				
Blood draw: RNA[20]	X									X				
Dosing GC4419/Placebo[21]		X	Weekdays when IMRT is given							X				
BUN, creatinine, and weight												X		
Survival Status[22]												X	X	X

BSA = body surface area; ECG = electrocardiogram; ECOG = Eastern Cooperative Oncology Group; HNC = head and neck cancer, IMRT = intensity-modulated radiation therapy; OM = oral mucositis; PE = physical examination; PK = pharmacokinetics

Table footnotes

[1] If a subject ends study participation early and/or withdraws consent after randomization, all last day of IMRT procedures should be completed.

[2] The Post-IMRT Weeks 1 and 2 Follow-up Visits will be scheduled based on the last day of IMRT. All subjects will be seen at 7 ± 2 calendar days from the last day of IMRT and 14 ± 2 calendar days from the last day of IMRT.

[3] See protocol Sections 7.1 and 7.2.

[4] The HNC history should include tumor HPV status, staging (AJCC 8th Edition) information, prior treatments, and confirmation of histopathological diagnosis of SCC. Medical conditions and illnesses that have occurred since the subject signed the ICF up until the date of randomization should be recorded as medical history. Medical history also includes tobacco and alcohol use history.

[5] At the Screening and Last Day of IMRT Visits, a complete physical examination will be conducted.

[6] Vital signs (temperature, systolic and diastolic blood pressures, heart rate, and respiration rate), and ECOG will be obtained and recorded at the Screening and Baseline Visits, once during Weeks 4 and 7, and at the Last Day of IMRT Visit. All vital signs should be measured following 2 minutes of rest in the sitting position. On Day 1 (Baseline) and Day 22, blood pressure must be measured at the following times: 1) within 30 minutes prior to the pre-GC4419/Placebo PK draw, and 2) after GC4419/Placebo infusion, prior to standing and prior to the End of GC4419/Placebo Infusion PK draw.

[7] For subjects receiving tri-weekly cisplatin, body surface area (BSA) will be recorded to confirm cisplatin dosing at the Baseline Visit and once during Weeks 4 and 7. For subjects receiving weekly cisplatin, BSA will be recorded to confirm cisplatin dosing at the Baseline Visit and once per week until chemotherapy is completed.

[8] Radiographic imaging must be performed within 60 days prior to the first day of IMRT for definitively treated subjects. Imaging can occur prior to the 60-day window for post-operatively treated subjects. Pre-surgical imaging should be captured if available; post-surgical imaging will be accepted otherwise. Imaging must occur at Month 12 post-IMRT for all subjects. Subjects treated definitively (as opposed to post-operatively) will also undergo imaging at Month 3 post-IMRT to assess tumor response/clearance to the degree possible. Radiographic imaging is highly recommended at any post-IMRT follow-up visit at which disease progression is suspected by the treating physician. If radiographic imaging is performed, both local/regional recurrence and distant metastases should be evaluated.

[9] At the Last Day of IMRT Visit, a clinical tumor assessment will be conducted. A head/neck/oral exam is sufficient on the Last day of IMRT if disease progression is not suspected. If disease progression is suspected, a laryngopharyngoscopy should be conducted. At Months 3, 6, 9, and 12 a laryngopharyngoscopy (mirror and/or fiberoptic and/or direct procedure) is required to evaluate local/regional or distant progression. However, if local/regional progression or recurrence has already been discovered at a previous visit, a laryngopharyngoscopy is no longer required at future visits. If distant progression is discovered at a previous visit, a laryngopharyngoscopy is still required at subsequent visits to assess the subject for local/regional progression/recurrence. A head/neck/oral exam should always be conducted at Months 3, 6, 9, and 12 post-IMRT.

[10] All concomitant therapies (e.g., prescription and over-the-counter medications) taken by subjects on or after the date of randomization through 30 days following the last dose of GC4419/Placebo, IMRT or cisplatin (i.e. whichever occurs last) will be collected in the CRF. Any concomitant therapies used to treat any serious or related adverse event will be recorded in the CRF.

[11] AEs and SAEs with onset dates on or after the date of randomization through 30 days following the last dose of GC4419/Placebo, IMRT or cisplatin (i.e. whichever occurs last) will be recorded on the CRF. All subjects with SAEs will be followed until the events resolve, stabilize, become chronic, the subject completes the study, or the subject is lost to follow-up.

[12] Ventricular rate and P-R, QRS, QT, and QTc intervals will be assessed and recorded at Screening.

[13]All OM assessments must be performed by trained evaluators. The extent of the subject's OM will be scored using the WHO OM toxicity scale. OM assessments will be completed at the Screening Visit, at the Baseline Visit, and twice weekly (no less than two days apart) within each 5-day IMRT period. For Week 1, the first OM assessment will occur at the Baseline Visit and one additional OM assessment must occur at least two days later during the week. The extent of the subject's OM will be scored using the WHO OM toxicity scale. All subjects will be seen at 7 ± 2 calendar days from the last day of IMRT and 14 ± 2 calendar days from the last day of IMRT.

[14]For a woman of childbearing potential, serum pregnancy test must be performed at the Screening Visit.

[15]Clinical laboratory measurements will be conducted at the Screening Visit, twice during Week 1 (once at the Baseline Visit and again on Day 3, 4 or 5), and once weekly from Week 2 through the last day of IMRT. Clinical laboratory measurements at these visits will include the hematology profile (hemoglobin, hematocrit, red blood cell count, white blood cell count with differential, and platelet count. Differential to include total neutrophils, lymphocytes, monocytes, eosinophils, and basophils) and chemistry profile (glucose, BUN, creatinine, sodium, potassium, calcium, albumin, total protein, total bilirubin, direct bilirubin, alkaline phosphatase, ALT (SGPT), AST (SGOT), chloride, phosphate, bicarbonate, magnesium).

[16]If safety labs have already been drawn during the study week in which the last day of IMRT or early termination visit falls, then lab safety tests (chemistry and hematology profiles) do not need to be conducted on the last day of IMRT or at the early termination visit. If safety labs have not been drawn during the current study week at the time of the early termination visit or on the last day of IMRT, then safety labs should be drawn on that day. Safety labs only need to be drawn once per study week after Week 1.

[17]Blood samples will be collected for serum cotinine test at Baseline (Day 1), once during Week 4, and on the Last Day of IMRT.

[18]Blood samples will be collected for GC4419 pharmacokinetic (PK) measurements at Baseline (Day 1), Day 2, and Day 22. See Table 4 for additional information.

[19]For cytokine analysis, blood samples will be collected at the Screening Visit (within 28 days of IMRT start but at least 72 hours prior to Baseline), at the Baseline Visit, Week 2 OM Visit 2, Week 4 OM Visit 2, Week 6 OM Visit 2, and at the Last Day of IMRT Visit. Whenever possible, blood draws for cytokine analysis should be conducted at OM Visit 2 for each protocol-specified week; however, if blood draws must be taken on another day within a given week, it will not be considered a protocol deviation.

[20]If the subject consents separately, blood samples will be collected at the Screening Visit (within 28 days of IMRT start but at least 72 hours prior to Baseline) and at the Last Day of IMRT Visit for the assessment of gene expression patterns prior to receiving the first dose of GC4419 and upon completion of GC4419 doses.

[21]GC4419/Placebo will be administered up to 35 times: weekly, Monday through Friday, beginning at Baseline (IMRT Day 1) and continuing through the 35th dose. IMRT must begin no longer than 60 min following the end of the GC4419/Placebo infusion. If IMRT is not received on any given day due to a treatment break or unforeseen circumstances, GC4419/Placebo should not be administered on that day. Subjects should resume GC4419/Placebo administration when IMRT resumes. If GC4419/Placebo was already administered on a given day and IMRT is not administered due to unforeseen circumstances, that day's GC4419/Placebo dose will still count as one of the 35 doses.

[22]Subjects will be followed for survival for two years. The subject's survival status will be assessed at the following time points: Every 3 months, ± 30 days, throughout the 1st year post-IMRT (Months 3, 6, 9, and 12), and Every 4 months, ± 30 days, throughout the 2nd year post-IMRT (Months 16, 20, and 24).

APPENDIX 2. WORLD HEALTH ORGANIZATION (WHO) SCORE OF ORAL MUCOSITIS

Grade	Scoring Criteria
Grade 0:	None
Grade 1:	Erythema and Soreness; No ulcers
Grade 2:	Ulcers; Able to eat a solid diet
Grade 3:	Ulcers; Requires a liquid diet
Grade 4:	Ulcers; Not able to tolerate a solid or liquid diet; Requires IV or tube feeding

APPENDIX 3. PERFORMANCE STATUS CONVERSION

Performance Status Conversion: ECOG - Karnofsky			
ECOG		Karnofsky	
Score	Description	Score	Description
0	Fully active, able to carry on all pre-disease performances without restriction.	100	Normal, no complaints, no evidence of disease
		90	Able to carry on normal activity, minor signs or symptoms of disease.
1	Restricted in physically strenuous activity, but ambulatory and able to carry out work of a light or sedentary nature, office work	80	Normal activity with effort, some signs or symptoms of disease
		70	Cares for self, unable to carry on normal activity or do active work
2	Ambulatory and capable of all self-care, but unable to carry out any work activities; Up and about more than 50% of waking hours	60	Requires occasional assistance, but is able to care for most of his/her needs
		50	Requires considerable assistance and frequent medical care
3	Capable of only limited self-care, confined to bed or chair more than 50% of waking hours	40	Disabled, requires special care and assistance
		30	Severely disabled, hospitalization indicated; Death not imminent
4	Completely disabled; Cannot carry on any self-care; Totally confined to bed or chair	20	Very sick, hospital indicated, death not imminent
		10	Moribund, fatal processes progressing rapidly
5	Death	0	Death

APPENDIX 4. NATIONAL CANCER INSTITUTE-COMMON TERMINOLOGY CRITERIA FOR ADVERSE EVENTS, VERSION 5.0

See the following website link for the complete NCI-CTCAE Version 5.0:

https://ctep.cancer.gov/protocolDevelopment/electronic_applications/docs/CTCAE_v5_Quick_Reference_8.5x11.pdf

APPENDIX 5. RECOMMENDED REGIMENS FOR HIGH-RISK CHEMOTHERAPY-INDUCED NAUSEA AND VOMITING (CINV) PER ASCO AND MASCC GUIDELINES

Note: Both ASCO and MASCC define single-agent IV cisplatin as a "high risk" regimen for CINV.

ASCO: http://ascopubs.org/doi/pdf/10.1200/JCO.2017.74.4789

Table 7: Antiemetic Dosing for Adults – High Risk (Cisplatin)

Drug Class	Agent	Dose on Day of Chemotherapy	Dose(s) on Subsequent Days
NK_1 Receptor Antagonist	Aprepitant	125 mg oral	80 mg oral; days 2 and 3
	Fosaprepitant	150 mg IV	Day 1 only
	Netupitant-palonosetron	300 mg netupitant/0.5 mg palonosetron<br>oral in single capsule (NEPA)	Day 1 only
	Rolapitant	180 mg oral	Day 1 only
5-HT_3 Receptor Antagonist	Granisetron	2 mg oral OR 1 mg OR 0.01 mg/kg IV OR 1 transdermal patch OR 10 mg SC	Day 1 only
	Ondansetron	8 mg oral twice daily OR 8 mg oral dissolving tablet twice daily OR three 8 mg oral soluble films OR 8 mg or 0.15 mg/kg IV	
	Palonosetron	0.50 mg oral OR 0.25 mg IV	
	Dolaseetron	100 mg oral ONLY	
	Tropisetron	5 mg oral OR IV	
	Ramosetron	0.3 mg IV	
Corticosteroid if aprepitant is used*	Dexamethasone	12 mg oral OR IV	8 mg oral OR IV; once daily on days 2-4*

Corticosteroid if fosaprepitant is used*	Dexamethasone	12 mg oral OR IV	8 mg oral OR IV day 2; 8 mg oral OR IV twice daily on days 3-4*
Corticosteroid if netupitant-palonosetron is used*	Dexamethasone	12 mg oral OR IV	8 mg oral OR IV; once daily on days 2-4*
Corticosteroid if rolapitant is used	Dexamethasone	20 mg oral OR IV	8 mg oral OR IV; once daily on days 2-4
Olanzapine	Olanzapine	10 mg oral	10 mg oral on days 2-4

*Presumes patients are receiving an NK1 antagonist. If they are not, the **dexamethasone dose should be adjusted** to 20 mg on day 1 and 16 mg on days 2-4.

MASCC: full updated guidelines may be found at: http://www.mascc.org/antiemetic-guidelines (2016)

For the prevention of non-AC highly emetogenic chemotherapy, a three-drug regimen including single doses of a 5-HT3 RA, dexamethasone and an NK1 RA (aprepitant, fosaprepitant, netupitant or rolapitant), given before chemotherapy is recommended. In patients receiving non-AC highly emetogenic chemotherapy treated with a combination of an NK1 RA, a 5-HT3 RA and dexamethasone to prevent acute nausea and vomiting, dexamethasone on days 2–4 is suggested to prevent delayed nausea and vomiting. If aprepitant 125 mg is used in day 1, then dexamethasone 8 mg x 1 (days 2-4) + aprepitant 80 mg x 1 (days 2-3) OR dexamethasone 8 mg x 2 (days 2-4) + metoclopramide 20 mg x 4 (days 2-4). Please note that this dosage of metoclopramide derives from a phase III study and some regulatory authorities like EMA now recommend a maximum 0.5 mg/kg total daily dose.

APPENDIX 6. DAILY ORAL CARE RECOMMENDATIONS

The following protocol is strongly recommended for all dentulous or partially dentulous study participants to optimize oral health during radiation therapy and minimize the risk of post-radiation dental sequelae.

1. Diet – Minimize foods that contain refined sugars such as cookies, cakes, candy, sugar-containing drinks, etc. Favor fruits and vegetables.
2. Avoid alcohol, tobacco products, and carbonated beverages.
3. Practice scrupulous oral hygiene:
    a. Use a soft toothbrush or an electric toothbrush at least twice daily.
    b. Use fluoride-containing toothpaste or fluoride gel. When mouth becomes sore from mucositis continue to use toothbrush dipped in bland oral rinse.
    c. Clean spaces between the teeth using dental floss or similar device.
    d. If mouth is too sore to carry this out, rinse with topical anesthetic rinse to ensure completion of daily oral care.
4. Chew sugarless chewing gum after meals. Avoid other forms of chewing gum.
5. Use bland oral rinse three times a day until mouth becomes sore, then increase to every hour when mucositis begins.

Recipe for bland oral rinse: 1 teaspoon salt and 1 teaspoon baking soda in 4 cups of water

APPENDIX 7. CYP2D6 SUBSTRATES

Examples of CYP2D6 Substrates:

Beta Blockers	Antidepressants	Antipsychotics	Others
carvedilol<br>S-metoprolol<br>propafenone<br>timolol	amitriptyline<br>clomipramine<br>desipramine<br>duloxetine<br>fluoxetine<br>imipramine<br>paroxetine	haloperidol<br>risperidone<br>thioridazine	aripiprazole<br>atomoxetine<br>codeine<br>dextromethorphan<br>doxepine<br>flecainide<br>mexiletine<br>ondansetron<br>oxycodone<br>risperidone<br>tamoxifen<br>tramadol<br>venlafaxine

Reference the following link for a complete and current list of CYP2D6 substrates: https://drug-interactions.medicine.iu.edu/Clinical-Table.aspx

APPENDIX 8. SUMMARY OF CHANGES IN AMENDMENT 4

The GTI-4419-301 Protocol Amendment 3, dated 27 November 2018 is replaced by this Protocol Amendment 4, dated 13 July 2020. This Protocol Amendment 4 applies to all sites.

Listed below is a summary of substantive changes incorporated into Amendment 4. Administrative changes, such as cross-linking and typographical/grammatical corrections have also been made, which may not be summarized. A redline version which identifies all non-formatting changes (i.e., "was-is" document) has been provided to all sites. (Note: The page numbers in the final version may not match exactly the page numbers in the redline version due to the listing of previous and new text).

1. **Change:** Updated number of participating sites to 100.

   Rationale: Increase in number of participating sites.

2. **Change:** Updated total number of subjects enrolled to 450.

   Rationale: Subject number increased to enhance the statistical power of the study to support a more robust statistical finding for a single pivotal efficacy study NDA and to support the overall final safety database intended for the marketing application.

3. **Change:** Updated estimated enrollment period to 32 months.

   Rationale: Estimate increased due to increase in subject number.

4. **Change:** Updated study period.

   Rationale: Study timeline extended due to increase in subject number.

5. **Change:** Added study period definitions.

   Rationale: To align between protocol and final Statistical Analysis Plan.

6. **Change:** Added information regarding the sponsor's dosing suspension decision. Clarified that the 28 subjects on treatment during the dosing suspension are excluded from the ITT.

   Rationale: New information.

7. **Change:** Added results from the RTOG 1016 study to background section.

   Rationale: New information.

8. **Change:** Clarified that once prepared, GC4419/Placebo saline mixtures should spend as little time as possible outside refrigerated conditions, not exceeding more than 6 hours at ambient temperature.

   Rationale: Clarification.

9. **Change:** Added information regarding description of GC4419 and instructions regarding required use of a 0.2 or 0.22 micron syringe filter during preparation of GC4419/Placebo or inline filter during administration of GC4419/Placebo to align with the revised Pharmacy Manual.

Rationale: To align between the protocol and revised Pharmacy Manual the description of the filtering requirement in the preparation process as a precautionary measure in the event that visible particles are found in the vialed drug product.

10. **Change:** Clarified that all medication restrictions begin on Day 1 of IMRT and GC4419/Placebo.

    Rationale: Clarification.

11. **Change:** Clarified that ventral tongue is included in the list of oral sites in Inclusion Criterion 2.

    Rationale: Clarification.

12. **Change:** Clarified that Exclusion Criterion 2 is intended to exclude M1 disease (distant metastasis).

    Rationale: Clarification.

13. **Change:** Clarified that prior induction chemotherapy in Exclusion Criterion 4 refers to prior induction chemotherapy for current HNC.

    Rationale: Clarification.

14. **Change:** Clarified that history of active hepatitis B/C is exclusionary per Exclusion Criterion 12.

    Rationale: Clarification.

15. **Change:** Clarified that cumulative incidence of severe OM and of WHO Grade 4 OM are defined as the proportion of subjects with any occurrence of WHO Grade 3-4 OM and WHO Grade 4 OM respectively.

    Rationale: Clarification.

16. **Change:** Clarified pre-treatment imaging requirements. Radiographic tumor imaging must occur within 60 days prior to the first day of IMRT (Baseline) for definitively treated subjects. Pre-surgical imaging should be used if available for post-operatively treated subjects, even if the imaging occurs prior to the 60-day window. If pre-surgical imaging was not done, post-surgical imaging can be used.

    Rationale: Clarification.

17. **Change:** Clarified that if local/regional progression or recurrence has already been discovered at a previous post-IMRT visit, a laryngopharyngoscopy is no longer required at future visits. However, if distant progression is discovered at a previous visit, a laryngopharyngoscopy is still required at subsequent visits to assess the subject for local/regional progression/recurrence. A head/neck/oral exam must always be conducted at Months 3, 6, 9, and 12 post-IMRT.

    Rationale: Clarification.

18. **Change:** Added blood urea nitrogen (BUN), creatinine, and weight assessments at 3, 6, 9, and 12 Months post-IMRT.

    Rationale: Additional assessments included to support exploratory analysis.

19. **Change:** Provided clarification and additional detail on secondary and exploratory objectives/endpoints.

    Rationale: Updated to align protocol and final Statistical Analysis Plan.

20. **Change:** Updated the description of the planned multiplicity testing on the secondary endpoints from Hochberg to Holm-Bonferroni.

    Rationale: Updated to align protocol and final Statistical Analysis Plan.

21. **Change:** Updated "glutamine applied topically" to "glutamine in any form" under prohibited medications.

    Rationale: Clarification per protocol clarification memorandum released 03-Jan-2020.

22. **Change:** Added information regarding GC4419 and CYP2D6 drug-drug interaction.

    Rationale: Clarification and update consistent with prior investigator notification.
